# High resolution landscape of ribosomal RNA processing and surveillance

**DOI:** 10.1093/nar/gkae606

**Published:** 2024-07-12

**Authors:** Weidong An, Yunxiao Yan, Keqiong Ye

**Affiliations:** Key Laboratory of RNA Science and Engineering, CAS Center for Excellence in Biomacromolecules, Institute of Biophysics, Chinese Academy of Sciences, Beijing 100101, China; Key Laboratory of RNA Science and Engineering, CAS Center for Excellence in Biomacromolecules, Institute of Biophysics, Chinese Academy of Sciences, Beijing 100101, China; University of Chinese Academy of Sciences, Beijing 100049, China; Key Laboratory of RNA Science and Engineering, CAS Center for Excellence in Biomacromolecules, Institute of Biophysics, Chinese Academy of Sciences, Beijing 100101, China; University of Chinese Academy of Sciences, Beijing 100049, China

## Abstract

Ribosomal RNAs are processed in a complex pathway. We profiled rRNA processing intermediates in yeast at single-molecule and single-nucleotide levels with circularization, targeted amplification and deep sequencing (CircTA-seq), gaining significant mechanistic insights into rRNA processing and surveillance. The long form of the 5′ end of 5.8S rRNA is converted to the short form and represents an intermediate of a unified processing pathway. The initial 3′ end processing of 5.8S rRNA involves trimming by Rex1 and Rex2 and Trf4-mediated polyadenylation. The 3′ end of 25S rRNA is formed by sequential digestion by four Rex proteins. Intermediates with an extended A1 site are generated during 5′ degradation of aberrant 18S rRNA precursors. We determined precise polyadenylation profiles for pre-rRNAs and show that the degradation efficiency of polyadenylated 20S pre-rRNA critically depends on poly(A) lengths and degradation intermediates released from the exosome are often extensively re-polyadenylated.

## Graphical Abstract

**Graphical Abstract:**
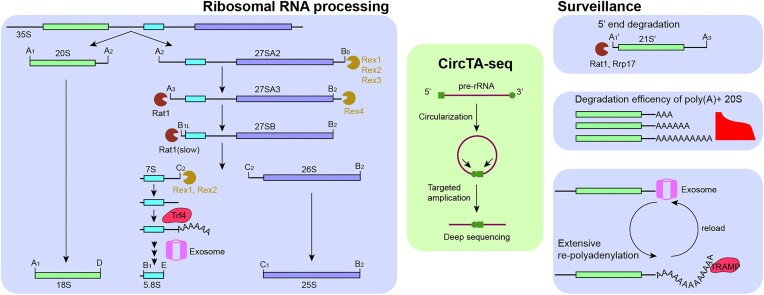

## Introduction

Ribosomal RNAs (rRNAs) make up approximately 80% of total RNA in eukaryotic cells, and their synthesis is a fundamental, complicated and conserved cellular activity (1–6). The primary precursor rRNA (pre-rRNA), which encodes the 18S rRNA in the small subunit ribosome (SSU) and the 5.8S and 25S rRNAs in the large subunit ribosome (LSU), is transcribed by RNA polymerase I (Pol I) from hundreds of rDNA genes. Additionally, the 5S rRNA in the LSU is independently transcribed by Pol III. Pre-rRNAs associate with ribosomal proteins and numerous assembly factors into various preribosomes that mediate modification, processing and folding of rRNA (7,8).

**Figure 1.**
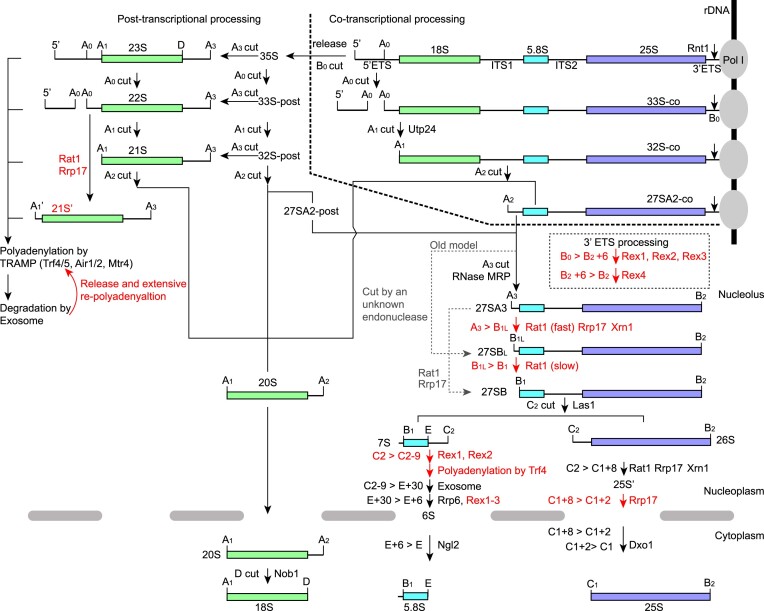
Pathways of rRNA processing and surveillance in *Saccharomyces cerevisiae*. The primary transcript 35S is transcribed by Pol I from rDNA in the nucleolus and released by Rnt1-mediated cleavage at site B0. The 35S is sequentially cleaved at sites A0, A1 and A2, yielding 33S, 32S, 27SA2 and 20S pre-rRNAs. Co- or post-transcriptionally generated 33S, 32S and 27SA2 species are suffixed with -co or -post. The 20S is exported to the cytoplasm and processed into mature 18S rRNA. The 27SA2 is processed into mature 5.8S and 25S rRNAs by endonucleolytic cleavage and exonucleolytic trimming. In a minor pathway, the 35S is cut at the A3 site, releasing the 23S species subjected to surveillance. The newly discovered or revised steps are highlighted in red.

In addition to mature rRNAs, the primary pre-rRNA also contains the 5′ and 3′ external transcribed spacer (5′ ETS and 3′ ETS) and the internal transcribed spacer 1 and 2 (ITS1 and ITS2). These spacers need to be removed through a complex processing pathway, which is best characterized in the yeast *Saccharomyces cerevisiae* (Figure 1) (1,4,5). The longest detectable pre-rRNA, known as 35S, is released by Rnt1-mediated cleavage at the B0 site of 3′ ETS (9,10). Nevertheless, assembly and processing of rRNA already start during transcription (11,12). The 5′ region of 35S is assembled into a 90S preribosome. Within the 90S, the A0 and A1 sites of the 5′ ETS are sequentially cleaved to form the mature 5′ end of 18S rRNA and yield the 33S and 32S intermediates. Endonucleolytic cleavage at site A2 separates the 20S and 27SA2 pre-rRNAs. The 20S, packed in a pre-40S ribosome, is rapidly exported to the cytoplasm, where the D site is processed to form the mature 3′ end of 18S.

The 27SA2 pre-rRNA is further processed into 5.8S and 25S rRNAs within maturating pre-60S ribosomes. The 5′ and 3′ ends of 5.8S and 25S rRNAs are all generated through an initial endonucleolytic cleavage and subsequent trimming by multiple exonucleases. To form the major form of the 5′ end of 5.8S, the ITS1 in 27SA2 is cleaved at site A3 by RNase MRP into the 27SA3 pre-rRNA, which is trimmed to site B1 by the 5′-3′ exonucleases Rat1, Rrp17 and Xrn1, yielding the 27SB pre-rRNA (13,14). Xrn1, a homolog of Rat1, predominantly localizes in the cytoplasm but seems to have some nuclear activities (13,15). The 5′ end of 5.8S has a minor long form located ∼7–8 nucleotides (nt) upstream of site B1, which is thought to be directly cleaved by an unidentified endonuclease (13,16). The ITS2 in 27SB is cleaved at site C2 by Las1, producing the 7S and 26S pre-rRNA (17–19). The 7S is trimmed by a number of 3′-5′ exonucleases the exosome/Rrp44, Rrp6, Rex proteins and Ngl2 to form the 3′ end of 5.8S (20–28). The 26S is digested by 5′-3′ exonucleases Rat1, Rrp17, Xrn1 and Dxo1 to form the 5′ end of 25S (14,17,19,29,30). It is poorly known how the 3′ end of 25S is processed from the B0 site, and only Rex1 is implicated in the process (27,31). Rex1 belongs to the DEDD family 3′-5′ exonuclease, which also includes Rex2, Rex3 and Rex4 in yeast (32).

Pre-rRNA processing is monitored by the nuclear RNA surveillance system (33,34). Aberrant processing intermediates are appended with a short polyadenosine (poly(A)) tail that activates the nuclear exosome for subsequent degradation (35–37). Polyadenylation is catalyzed by the TRAMP complex consisting of the poly(A) polymerase Trf4 or Trf5, the zinc knuckle protein Air1 or Air2, and the RNA helicase Mtr4. The nuclear exosome consists of a 9-subunit barrel-like core structure and two 3′-5′ exonucleases Rrp44 and Rrp6. Rrp44 is bound at the bottom of the barrel and digests substrate RNAs that thread through the central channel or bind via a direct route (38–40). Rrp6 is placed at the top of the barrel and can have distinct substrate specificity from Rrp44. Mtr4 is an essential co-factor of the nuclear exosome and unwinds secondary structures of substrate RNAs. In rRNA processing, the nuclear exosome is involved in 3′ processing of 7S pre-rRNA and degradation of aberrant processing intermediates and the excised 5′ ETS fragment (20–22). The 23S, 22S and 21S pre-rRNAs are commonly detected aberrant intermediates (1,41). The 23S results from cleavage of the primary transcript at site A3 in the absence of cleavage at sites A0, A1 and A2. The 22S and 21S pre-rRNAs could be derived from the 23S through cleavage at sites A0 and A1 or from the 33S and 32S through cleavage at site A3.

The major steps of rRNA processing and surveillance have long been established in yeast, but there are still steps that are not or incompletely characterized, such as 3′ ETS processing. Processing intermediates of rRNA are traditionally analyzed by northern hybridization and primer extension. These methods are limited in resolution and sensitivity, and particularly difficult for analyzing intermediates with continuous ends that are generated during LSU rRNA processing and RNA degradation. In this study, we developed a high-throughput method to profile processing and degradation intermediates of rRNA. By analyzing the complex population of pre-rRNA and its disturbance by 15 mutants with unprecedented precision, sensitivity and resolution, we obtained major mechanistic understanding into rRNA processing and surveillance.

## Materials and methods

### Yeast strains

All yeast strains were derived from BY4741 (*MATa, his3Δ1, leu2Δ0, met15Δ0, ura3Δ0*). The *NOC4-TAP* and *NOP7-TAP* strains were purchased from Open Biosystems. Genes were engineered with homologous recombination. To create a conditional expression strain, a cassette composed of the *natNT2* gene, the *GALL* promoter and a *3xHA*-tag was amplified from plasmid pYM-N28 and inserted upstream of the target gene (42). For gene deletion, the target gene was replaced by the *natNT2* gene. All used strains are listed in Supplementary Table S1.

### Yeast culture and RNA isolation

Yeast cells were commonly grown in YPD medium (1% yeast extract, 2% peptone, 0.0003% adenine and 2% glucose). Conditional expression strains were first cultured in YPG medium (1% yeast extract, 2% peptone, 0.0003% adenine and 2% galactose) and shifted into YPD medium for 14 h to deplete target proteins. Two liters of cells grown to OD_600_ <2.0 were collected for preribosome purification. TAP-tagged proteins were purified via IgG-coated magnetic beads as described (43). RNAs were extracted from the beads with TRIzol (Invitrogen). Total RNA was extracted from 10 ml of wild-type cells with hot phenol.

### CircTA-seq

**Figure 2.**
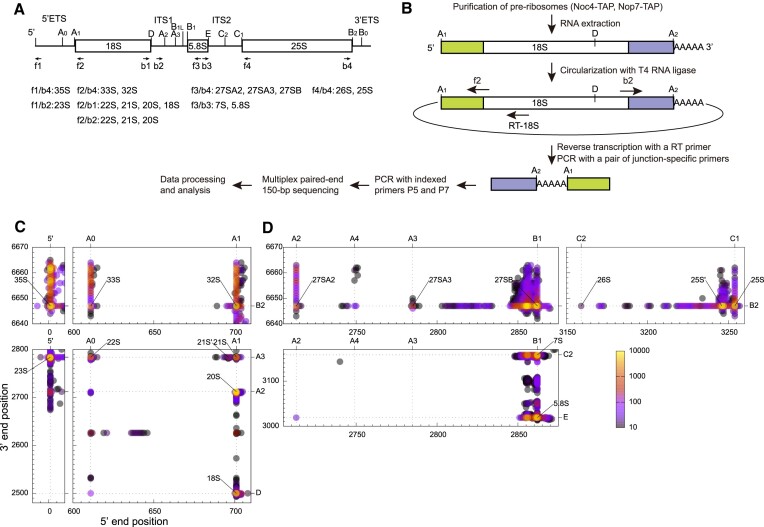
Profiling of rRNA processing intermediates by CircTA-seq. (**A**) Domain diagram of 35S pre-rRNA showing mature rRNA regions, spacers and processing sites. The location of primers is marked. Pre-rRNAs targeted by 8 primer pairs are listed. (**B**) Flowchart of CircTA-seq. A polyadenylated 20S species is illustrated. The detected 5′ and 3′ end sequences of RNA are shown as green and blue boxes, respectively. (C-D) 2D plot of processing intermediates of 18S (**C**) and 5.8S/25S (**D**) rRNAs. Each dot represents an RNA species with its 5′ and 3′ end positions in 35S pre-rRNA as x- and y-coordinates and is color coded by RNA abundance. The 35S, 33S-32S, 27S, 26S-25S, 23S, 22S-18S and 7S-5.8S panels are based on the primer pairs f1/b4, f2/f4, f3/b4, f4/b4, f1/b2, f2/b1 and f3/b3, respectively. A total of 100000 reads are analyzed for each dataset. Species with at least 6 reads are displayed. Intermediates of 18S and 5.8S/25S are based on the *WT/NOC4_1* and *WT/NOP7_1* samples, respectively.

Immunoprecipitated RNA (1μg) or total RNA (5μg) were ligated with T4 RNA ligase (Takara) at 16°C for 16 h. Circularized RNA was reverse transcribed into cDNA using AMV reverse transcriptase (Promega) and one of primers RT18S, RT58S and RT25S (Supplementary Table S2). Junction sequences of a related set of circularized pre-rRNAs were amplified by PCR for 28 cycles using KOD-Plus DNA polymerase (Toyobo) and a specific primer pair composed of a forward primer f1, f2, f3 or f4 and a backward primer b1, b2, b3 or b4 (Figure 2A, Supplementary Table S2). The reverse transcription primer RT18S was used with the junction amplification primer f1 and f2, RT58S with f3 and RT25S with f4. PCR products were separated in agarose gels and purified with the Wizard SV Gel and PCR Clean-Up System (Promega). Sequencing libraries were amplified for 16 cycles using indexed primers P5 and P7 (Supplementary Table S2) and purified as above. Multiple libraries were mixed and sequenced with Illumina HiSeq X10 in the 150-bp paired-end mode by Annoroad Gene Technology. Each dataset generally contained several millions reads.

### Data processing

Reads were demultiplexed according to the index sequences in P5 and P7. Adaptor sequences were removed with Cutadapt using the option ‘-a TGGAATTCTCGGGTGCC -A AGATCGGAAGAGCGTCG -e 0.1 -m 25 –overlap 5 -q 20’ (44). The 35S rDNA (RND37-1, 6858 nt) and its upstream 1243-nt sequence were used as the reference for alignment. Read 1 was aligned by BLASTN with the option ‘-task ‘blastn-short’ -evalue 1e-3 -strand plus -outfmt 6’ (45). Read 2 was aligned similarly with the ‘-strand minus’ option.

Alignment outputs were processed with Python scripts (Supplementary Figure S1). Coordinates of aligned sequences on reads and the reference were extracted from BLASTN outputs. Read 2 was converted to its reverse complementary (RC) sequence. Depending on its size, a junction could be located within one read or across reads 1 and 2. In the case of single-read junctions, read 1 or read 2 would be aligned twice to separated places of the reference. The 5′ and 3′ aligned sequences of read corresponded to the 3′ and 5′ terminal sequences of RNA, respectively. The middle unaligned region of read, if it exists, was defined as the poly(A) region (Supplementary Figure S1A). In the case of cross-read junctions, read 1 and 2 were each aligned once and to different places. Two A-tracks were extracted from the unaligned regions of reads 1 and 2. The longer one was defined as the poly(A) region, which represented a low limit estimation. The exact length of poly(A) region cannot be determined for cross-read junctions. A poly(A) region must contain at least 90% adenine and contain no mutations if it is less than 10 nt.

A pre-rRNA species here also refers to its processing and degrading intermediates that have a 5′ and 3′ end located within a range of nucleotides to a processing site, as defined in Supplementary Table S3. All unassigned species are called UNK.

The 5′ and 3′ end of RNA cannot be unambiguously resolved from a circularized junction if they share a stretch of identical sequence with each other or with the appended poly(A) sequence (Supplementary Figure S1B). A set of rules are sequentially applied to resolve the junction (Supplementary Figure S1C). The overlapped region is first split to keep an intact 5′ end of pre-rRNA or then an intact 3′ end (Rule 1). If both options are not possible, the overlapped region is split at a random position (Rule 2). Additionally, if a 3′-extension contains 1–2 adenines, it is reassigned as a poly(A) region (Rule 3). This rule is needed to correctly analyze the 3′ end of 23S, 22S and 21S (CAA|ACA, sequence around the cleavage site ‘|’), 20S (AAC|ACA) and 18S (TTA|AAG). As these ends are formed by precise endonucleolytic cleavage, the appended 1–2 adenines should result from non-templated addition rather than incomplete exonucleolytic processing.

The 20S, 21S and 22S pre-rRNAs are detected by both primer pairs f2/b1 and f2/b2 (Figure 2A) and show similar profiles in the two datasets. The 20S-22S species detected by f2/b1 are less abundant due to the presence of 18S and used only for illustrating relative abundance of pre-rRNAs (Figure 2C). All other analyses of 20S–22S are based on the f2/b2 dataset.

## Results and discussions

### Profiling of rRNA processing intermediates

To map precise ends of rRNA processing intermediates, we developed a method comprising RNA circularization, targeted amplification, and deep sequencing (CircTA-seq) (Figure 2A, B). Additionally, pre-rRNAs were enriched through purification of preribosomes via a ribosome assembly factor fused with a tandem affinity purification (TAP) tag. Following circularization of pre-rRNAs, the junction sequences were amplified with reverse transcription and PCR (RT-PCR) using 8 pairs of primers targeting specific pre-rRNAs (Figure 2A, Supplementary Table S2) and subjected to paired-end 150-bp sequencing. A pipeline was developed to resolve the 5′ and 3′ end sequences of pre-rRNA and any extra poly(A) sequences (Supplementary Figure S1).

We mainly utilized Noc4 and Nop7 as bait to enrich pre-rRNAs. Noc4 is a component of nucleolar 90S preribosomes and primarily associates with precursors of 18S rRNA (46,47) (Figure 2C). Nop7 is a component of nucleolar and nucleoplasmic pre-60S particles and mainly associates with precursors of 5.8S and 25S rRNAs (48) (Figure 2D). Nevertheless, all types of pre-rRNAs can be detected for each bait, in part because 90S and early pre-60S are briefly linked during their co-transcriptional assembly and in part because contaminating pre-rRNAs can be efficiently amplified. The profile of pre-rRNA contaminants would resemble that of unenriched species from total RNA samples and is still valuable for assessing processing defects.

A number of pre-rRNAs with distinct ends are produced in rRNA processing (Figure 1). Their termini were precisely mapped (Supplementary Figure S2), leading to revision of the A0 and B0 processing sites. The A0 site is located between positions 610 and 611 of the 5′ ETS, differing from the previous assignments (49,50). The B0 site is located between positions 15 and 16 from the 3′ end of 25S, 1 nt downstream of its previous position (9).

To assess the function of 15 factors with known or potential roles in rRNA processing and surveillance, we individually deleted genes of non-essential proteins Xrn1, Rrp6, Nlg2, Rex1, Rex2, Rex3, Rex4, Trf4 and Trf5 and depleted expression of essential factors Rat1, Rrp17, Nme1 (the RNA component of RNase MRP), Rrp44 and Mtr4 and non-essential protein Rnt1 by placing their genes under the control of a *GAL* promoter (Supplementary Table S1). These conditional expression strains show severe growth defects in glucose medium, indicating efficient protein depletion (Supplementary Figure S3A). Most of the gene deletion strains grew normally, although the *xrn1*, *rrp6* and *trf4* deletion mutants display reduced growth particularly at 20°C (Supplementary Figure S3B). The wild-type (*WT*) strains and the *rex1*, *rex2*, *rex3*, *rex4*, *rat1* and *rrp17* mutants with unexpected rRNA processing defects were analyzed twice with CircTA-seq. The end and polyadenylation profiles of intermediates are shown in Supplementary Figures S4-S31 for 28 analyzed samples.

### 5′-Extended A1 ends

**Figure 3.**
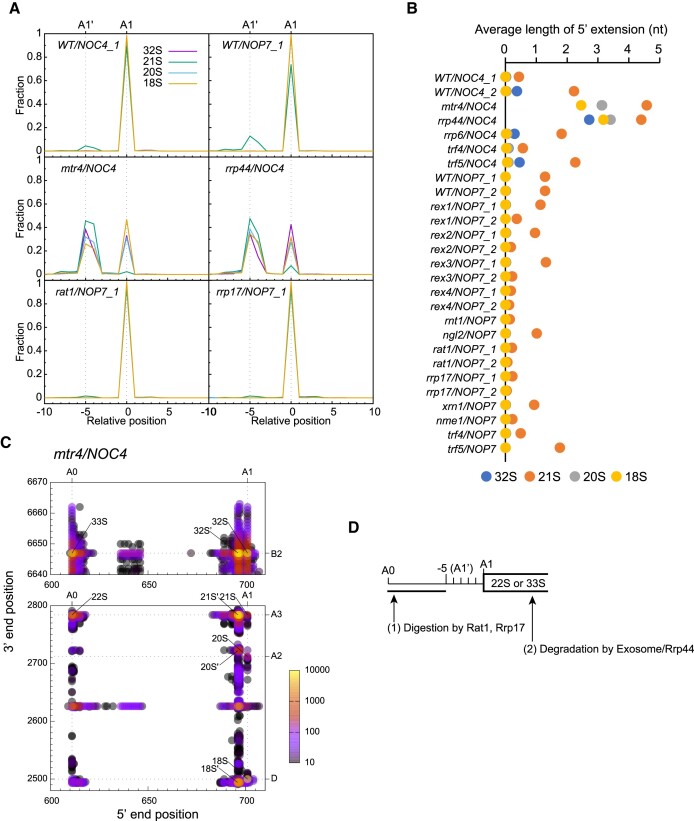
The 5′-extended 21S and 32S species are 5′ degradation intermediates. (**A**) Distribution of 5′ end of 32S, 21S, 20S and 18S species in selected strains. (**B**) Average length of 5′ extension for 32S, 21S, 20S and 18S species in all analyzed stains. RNA species that start 0–10 nt upstream of site A1 are averaged. (**C**) 2D plot of 33S-32S and 22S-18S species in the *mtr4* mutant. Each dot represents an RNA species with its 5′ and 3′ end positions in 35S pre-rRNA as x- and y-coordinates and is color coded by RNA abundance. 5′-extended forms of pre-rRNAs are marked by a prime. (**D**) Model showing formation and elimination of 5′-extended species of 21S and 32S.

The A1 site at the 5′ end of 18S rRNA is endonucleolytically cleaved in the 32S, 21S, 20S and 18S species. Surprisingly, we found that 7–32% of the 21S species are extended by 4–5 nt from site A1 in wild-type yeast (Figure 3A). The 5′-extended end, termed the A1' end, is 10–100 folds less abundant in the 32S and rare in the 20S and 18S. The 21S is an aberrant pre-rRNA targeted for RNA surveillance. To understand the biogenesis of the A1' end, we analyzed its distribution in various mutants (Figure 3A–B). Upon depletion of Mtr4 or Rrp44, 50–90% of all four species that normally start at the A1 site are extended to the A1' site, suggesting that the 5′-extended species are usually purged by the exosome/Rrp44. Deletion of *RRP6* only slightly increases the level of the A1' end (Figure 3B), suggesting that Rrp6 is minorly involved in degrading the 5′-extended species. Notably, the 5′-extended forms of 20S and 18S accumulated in the *mtr4* and *rrp44* strains are not authentic ones. They have a 3′ end close to, but not exactly at, the A2 or D site (Figure 3C) and should be derived from 3′-5′ exonucleolytic digestion of 5′-extended 21S, rather than normal endonucleolytic cleavage.

Loss of the 5′-3′ exonucleases Rat1 and Rrp17, but not Xrn1, reduces the 5′-extended form of 21S (Figure 3A, B). These results suggest that the 5′-extended 21S results from 5′-3′ exonucleolytic digestion of the 22S species. The 5′-extended 32S is likely generated in a similar way from the 33S. Earlier 5′ degradation intermediates of 22S and 33S are also detectable (Figure 2C and 3C).

In summary, we demonstrate that the 5′-extended 21S species represents an intermediate in 5′-3′ digestion of 22S by Rat1 and Rrp17 and is further eliminated by the exosome/Rrp44. In over 10-fold less frequency, the 5′-extended 32S is generated by 5′ degradation of 33S. Rat1 has been previously implicated in digesting polyadenylated pre-rRNAs (51). Rat1 strongly crosslinks to the upstream sequence of site A1 (52), supporting its digestion of 22S and 33S in addition to the previously discussed substrate, the released A0-A1 fragment. The 5′ extended form of 21S is also reduced in the *rex4*, *rnt1* and *nme1* mutants (Figure 3B). These mutants block the processing of 3′ ETS and ITS1 and might indirectly suppress the activities of Rat1 and Rrp17.

### 5′ End processing of 5.8S rRNA

In yeast and other eukaryotes, the 5′ end of 5.8S rRNA has a major short form (∼85% in yeast) that starts at site B1 (or site B1S) and a minor long form (∼15% in yeast) that starts at site B1L located ∼7–8 nt upstream of site B1. The two forms are considered to be generated in different pathways (13,14,16) (Figure 1). To produce the short form, the 27SA2 is cleaved by RNase MRP at site A3, and the resulting 27SA3 is then digested by the 5′-3′ exonucleases Rat1, Rrp17 and Xrn1 to site B1 (13,14). Alternatively, the 27SA2 can be directly cleaved by an unidentified endonuclease at site B1L to generate the long form (13,16). Importantly, the long form is regarded as an end product, but not a precursor of the short form.

**Figure 4.**
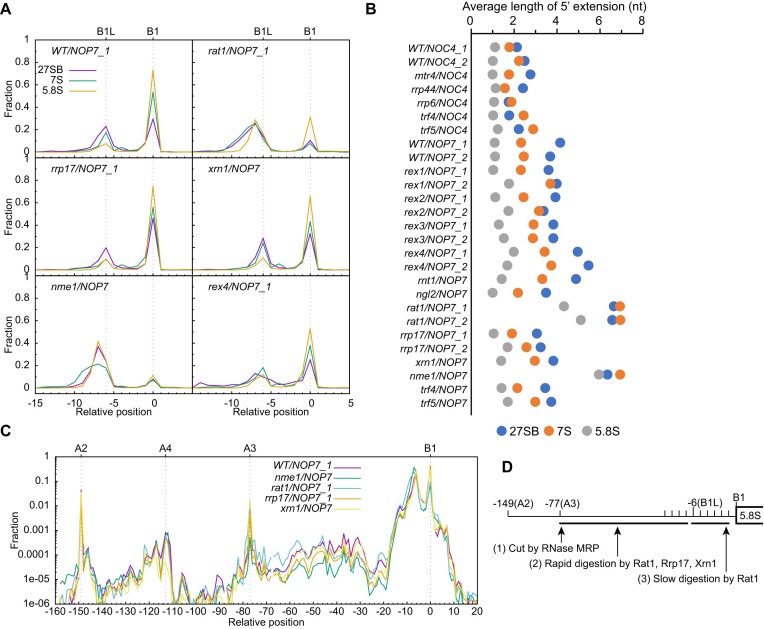
5′ end processing of 5.8S rRNA. (**A**) Distribution of 5′ end of 27SB, 7S and 5.8S species in selected strains. (**B**) Average length of 5′ extension for 27SB, 7S and 5.8S species in all analyzed strains. RNA species that start 0–15 nt upstream of site B1 are averaged. (**C**) Distribution of 5′ end of 27S processing intermediates in log scale. The 27SA2, 27SA3 and 27SB are all detected by the same primer pair f3/b4 and normalized together. (**D**) Model of 5′ end processing of 5.8S rRNA. Processing events are ordered.

By measuring the 5′ extension of all processing intermediates of 5.8S, we found that as 27SB is processed to 7S and 5.8S, the long form is progressively reduced and converted into the short form (Figure 4A, B). The mature B1 end increases from 28.9% to 53.9% and 73.9%. Clearly, the long form is an intermediate to the short form, rather than an end product of an alternative processing pathway. In addition, the long form of the 5′ end has a continuous distribution with major peaks at positions 6 and 7 upstream of site B1. However, endonucleases involved in rRNA processing commonly cut at a specific site.

Depletion of Rat1 leads to a strong increase of the long form of 5.8S (5.8SL) and a mild accumulation of processing intermediates of 27SA3 (Figure 4A–C), as expected (13). Loss of Rrp17 and Xrn1 do not significantly alter the 5′ end profile of 5.8S rRNA. These results show that Rat1 is the primary exonuclease in 5′ end processing of 5.8S, while Rrp17 and Xrn1 play only a minor role. RNase MRP-mediated A3 cleavage is critical for 5′ end formation of 5.8S (13,53,54). Consistently, depletion of the RNA component Nme1 of RNase MRP reduces the 27SA3 species and its processing intermediates and greatly increases 5.8SL (Figure 4A–C).

The 27S species with 5′ end between sites A2 and A3 are detectable (Figure 2D, 4C). The most abundant species start at around position 113 upstream of site B1 and share a similar 5′ end, known as the A4 site, with aberrant 5′-extended 5.8S species accumulated in Rrp5 mutants that block the A3 cleavage and delay ITS1 processing (55). Our data suggest that the A4 site is naturally processed in 27S pre-rRNAs, but the functional significance is unclear. The A4-starting 27S species (27SA4) may represent degradation intermediates or intermediates in a minor processing pathway of 27SA2. The 27SA4 level is not changed in the *rat1* and *rrp17* mutants, but reduced in the *xrn1* mutant, suggesting that the 5′-3′ exonuclease Xrn1 is involved in its formation. Xrn1 has been implicated in processing aberrant 5′-extended 5.8S species to 5.8SL (56,57).

Based on this and previous studies, we propose a unified model for 5′ end formation of 5.8S (Figure 4D). The A3 cleavage by RNase MRP creates an entry site that facilitates subsequent 5′-3′ exonucleolytic digestion. Rat1, as the primary exonuclease, rapidly digests ITS1 to positions 6–7 upstream of site B1. The remaining nucleotides are removed by Rat1 in a greatly reduced speed, probably due to structural barriers in pre-60S ribosomes. Rrp17 and Xrn1 play a minor role in these processing steps. In the determined nucleolar pre-60S structures where ITS1 processing takes place (58–60), the ITS1 is not visible and the 5′ end of 5.8S appears to be already processed. We expect that structural features that slow 5′ processing of 5.8S may be present in earlier, not-yet-resolved states.

### 3′ End processing of 5.8S rRNA

The 27SB pre-rRNA is cleaved by Las1 at the C2 site in ITS2 (17–19), separating the 7S and 26S species. Las1 forms a complex with Grc1, Rat1 and its cofactor Rai1 to coordinate the C2 cleavage and processing of 26S (18,19). Grc1 is a polynucleotide kinase that phosphorates the 5′-OH of 26S and facilitates subsequent digestion by Rat1-Rai1. The 3′ end processing of 7S occurs in three major steps: trimming by the exosome/Rrp44 to position ∼30 downstream of site E, yielding the 5.8S + 30 species (20–25), Rrp6-mediated digestion to position ∼6, yielding the 6S species (26), and removal of the remaining nucleotides by Rex1-3 proteins and Ngl2 (27,28).

**Figure 5.**
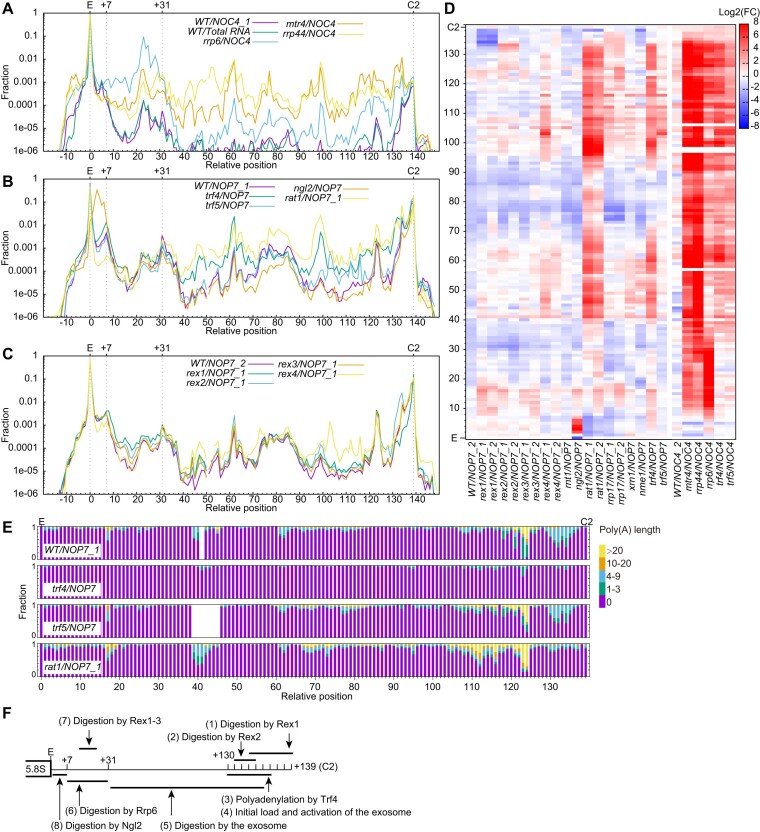
3′ end processing of 5.8S rRNA **(A–C)** Distribution of 3′ end of 7S processing intermediates in log scale. These species are detected by the primer pair f3/b3 and normalized together. (**D**) Heatmap showing log2 fold changes (FC) in fraction of 7S processing intermediates relative to *WT* samples. Samples purified via Noc4 and Nop7 are compared to the *WT/NOP7_1* (left part) and *WT/NOC4_1* samples (right part), respectively. (**E**) Stacked bar plot showing the distribution of poly(A) lengths for invidiual 7S processing intermediates. Poly(A) lengths are binned into five groups: 0, 1–3, 4–9, 10–20 and >20 nt. (**F**) Model of 3′ end processing of 5.8S rRNA. Processing events are ordered.

CircTA-seq analysis of *WT NOP7* samples reveals all 3′ processing intermediates of 5.8S, including the 6S and 5.8S + 30 species previously observed in exosome mutants (Figure 5B, C). One abundant intermediate with a 62-nt 3′ extension may be produced when nucleotides 1–59 of ITS2 are sequestered in the foot structure of pre-60S (61).

We show that depletion of Mtr4 or Rrp44 leads to a strong accumulation of most intermediates except those ending near site E and C2 (Figure 5A,D). Deletion of *RRP6* causes a strong accumulation of intermediates extended by 8–30 nt (5.8S + 30 species), as well as a mild accumulation of longer intermediates (Figure 5A, D). Our data confirm the specific roles of Mtr4, Rrp44 and Rrp6 in 7S processing and also suggest that they share some substates. As expected (28), deletion of *NGL2* causes a specific accumulation of 6S (Figure 5B, D).

The 7S and its slightly shortened intermediates are not significantly altered in the *mtr4*, *rrp44* and *rrp6* mutants, suggesting that 3′ terminal residues of 7S are processed by other enzymes (Figure 5A–D). Initial digestion of 7S appears to be a kinetically slow process, since the intermediate with one less residue is nearly as abundant as the unprocessed 7S and intermediates shortened by 2–6 nt are also fairly abundant (Supplementary Figure S2).

The 5′-3′ exonucleases Rex1, Rex2 and Rex3 were previously shown to process the 6S in a redundant manner (27). We detected a mild accumulation of intermediates 3′-extended by ∼10–20 nt upon deletion of each of their genes (Figure 5C–D). These results suggest that substrates of Rex1-3 are longer than the 6S and overlap with those of Rrp6. Moreover, we found additional roles of Rex exonucleases in 5.8S processing. Deletion of *REX1* increases the level of intact 7S by 1.5-fold and reduces 1–7 nt shortened intermediates by > 9 folds (Figure 5C–D), suggesting that Rex1 is involved in removing seven 3′ terminal residues of 7S. Deletion of *REX2* increases 5–8 nt shortened intermediates, suggesting that Rex2 acts after Rex1. Deletion of *REX4* causes a mild accumulation of intermediates that are processed by the exosome, similar with the *rat1* and *trf4* mutants. It is unclear whether these intermediates accumulate as direct substrates of Rex4 or as secondary effects of *REX4* deletion. Loss of Rex4 might suppress Rat1, which indirectly modulates initial processing of 7S and activation of the exosome (see below).

Polyadenylation by the TRAMP complex is important for exosome-mediated RNA degradation, but not known to participate exosome-mediated processing of 7S (36). Surprisingly, we found that deletion of *TRF4* results in the accumulation of intermediates extended by 40–70 nt and 90–133 nt (Figure 5B, D). No similar defect was observed in the *TRF5* deletion. These accumulated intermediates are substrates of the exosome, suggesting that Trf4 activates the exosome by polyadenylating processing intermediates of 7S. We analyzed the poly(A) distribution for all processing intermediates of 7S and found that two regions near the 3′ end of 7S (positions 118–124 and 130–136 from site E) are highly polyadenylated (Figure 5E). Positions 118–124 tend to have a longer poly(A) tail than positions 130–136. Deletion of *TRF4*, but not *TRF5*, eliminates polyadenylation of 7S (Figure 5E). Hence, the 7S processing defect in *trf4* is related to the loss of polyadenylation. Trf4 and Trf5 can have different substrate specificities in various RNA processing events (62–65). We conclude that Trf4 polyadenylates early processing intermediates of 7S, which facilitates loading and activation of the exosome. 7S substrates may be released during exosome processing and re-polyadenylated in a more extensive manner than initial intermediates, like in RNA degradation described below. This accounts for positions 118–124 having a longer poly(A) tail than the initial polyadenylated sites at positions 130–136.

Our findings have uncovered new steps in the 3′ end processing of 7S (Figure 5F). Following the C2 cleavage, the 3′ end of 7S is sequentially digested by Rex1 and Rex2 for about 9 nt. As no severe defect in 5.8S processing is present upon deletion of individual gene of Rex1 and Rex2, they may still play partially redundant roles in initial digestion of 7S, as seen in other processes (27). The briefly trimmed 7S species are polyadenylated by Trf4, which facilitates loading and activation of the exosome. The exosome is recruited to pre-60S ribosomes through a direct interaction between Mtr4 and the ribosome assembly factor Nop53 (66), but this does not obviate the requirement of substrate polyadenylation for activation.

Depletion of the 5′-3′ exonuclease Rat1 causes a strong accumulation of 3′-extended intermediates of 5.8S (Figure 5B, D), a phenotype previously observed but not mechanistically understood (17,51). The 3′ end profile of intermediates in *rat1* resembles that in *trf4* (Figure 5B and D), suggesting defects in polyadenylation and exosome activation. Polyadenylation is slightly increased at most positions of 7S in *rat1*, likely due to enhanced surveillance, but specifically reduced at positions 130–136 (Figure 5E). We interpret that polyadenylation at positions 130–136 is related to initial loading of the exosome, while polyadenylation at more upstream positions is associated with reloading of the exosome and RNA surveillance. In addition, the intermediate with one less nucleotide of 7S is reduced by 10-fold in *rat1* (Figure 5B, D), suggesting that digestion of the 3′ first residue of 7S is suppressed. Taking together, our results show that Rat1 is important for initial processing and polyadenylation of 7S and activation of the exosome. Rat1 is a component of the Las1 complex (19), which couples the C2 cleavage and 26S processing. In light of our data, a complete Las1 complex is also important for initializing 7S processing.

### 5′ End processing of 25S rRNA

Following the C2 cleavage, the ITS2 region of 26S is trimmed partially redundantly by the 5′-3′ exonucleases Rat1, Rrp17 and Xrn1 (14,17,19,29). The processing is so rapid that 26S and its processing intermediates cannot be detected by northern blot in wild-type yeast, except for the 25S' intermediate extended by 7–8 nt from site C1. The accumulation of 25S' is likely related to sequestering of the 3′ six nucleotides of ITS2 in the foot structure of pre-60S (61). The cytoplasmic 5′-3′ exonuclease Dxo1 is involved in processing 25S' to 25S and solely responsible for digesting the last 2 nt from site C1 (30).

**Figure 6.**
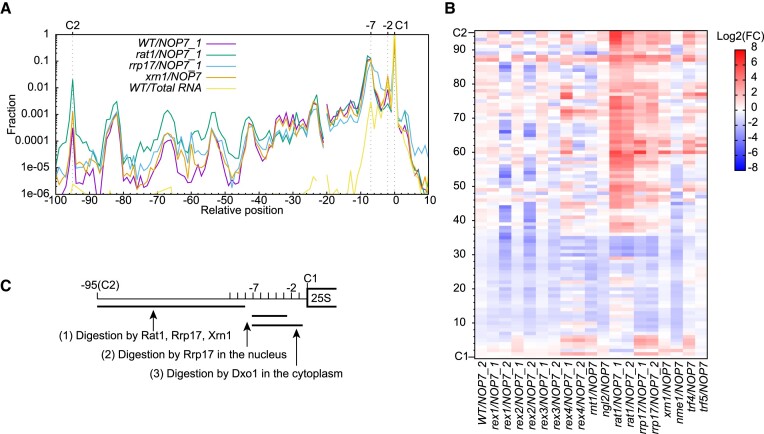
5′ end processing of 25S rRNA. (**A**) Distribution of 5′ end of 26S processing intermediates in log scale in selected strains. (**B**) Heatmap showing log2 fold changes in fraction of 26S processing intermediates relative to the *WT/NOP7_1* sample. All shown samples are purified via Nop7, which is required for enriching 26S species. (**C**) Model of 5′ end processing of 25S rRNA. Processing events are ordered.

We could detect the full set of processing intermediates of 26S in Nop7-bound pre-60S particles (Figure 6A). Without Nop7 enrichment, only 25S' and its processing intermediates are detected in total RNA and Noc4-bound particles. The 26S and its processing intermediates are > 2 orders of magnitude less abundant than 25S', attesting rapid digestion to position 8 upstream site C1.

Depletion of Rat1 results in a strong accumulation of 26S and its processing intermediates extended by >35 nt from site C1 (Figure 6A, B). These intermediates accumulate mildly in *rrp17* and little in *xrn1*. These results show that Rat1, Rrp17 and Xrn1 are increasingly less involved in 5′ end processing of 25S (14,17,19,29). Notably, intermediates extended by 3–6 nt from site C1 specifically accumulate in *rrp17*, but not in *rat1* and *xrn1*. This suggests that a fraction of 25S' is digested by Rrp17 in the nucleus to position 2 upstream of site C1 (Figure 6C). The unprocessed and partially processed 25S' are exported to the cytoplasm and further trimmed by Dxo1 to form the mature 5′ end of 25S (30). Loss of Rex4, Rnt1 and Trf4 also disturb distribution of 26S processing intermediates (Figure 6B), probably by indirectly suppressing Rat1 and Rrp17.

### 3′ End processing of 25S rRNA

**Figure 7.**
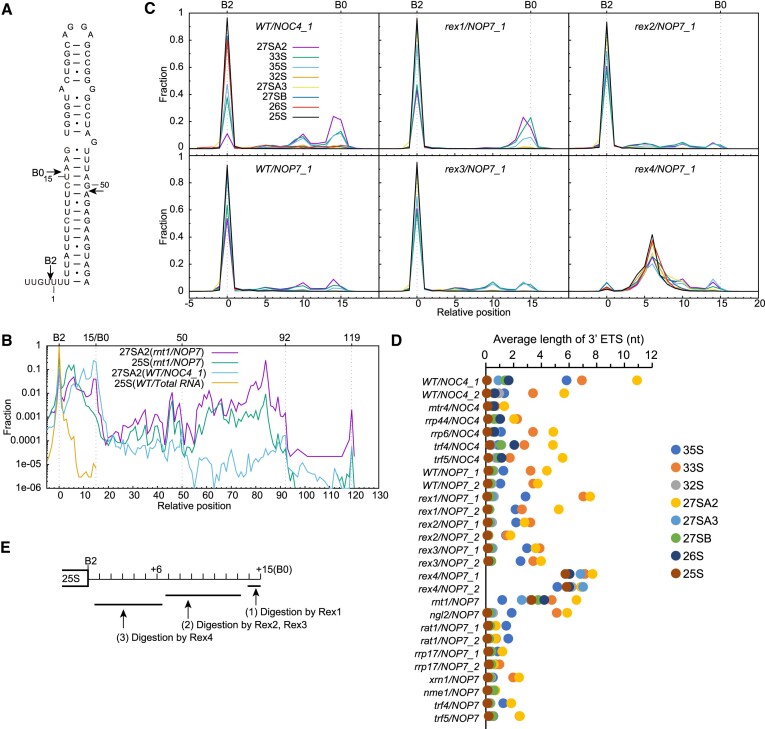
3′ end processing of 25S rRNA. (**A**) Secondary structure of the 3′ ETS hairpin. Processing sites are marked by arrows. (**B**) Distribution of 3′ end of 25S processing intermediates in log scale in *WT* and *rnt1* strains. (**C**) Distribution of 3′ end of 25S processing intermediates in *WT* and *rex1-4* deletion strains. (**D**) Average length of 3′ ETS for 25S precursors in all analyzed strains. Species terminating 0–20 nt downstream of site B2 are averaged. (**E**) Model of 3′ ETS processing. Processing events are ordered.

Processing of the 3′ ETS initiates with cleavage of a hairpin structure by the RNase III Rnt1 (9,10) (Figure 7A). The exposed B0 site is anticipated to be trimmed by 3′-5′ exonucleases to site B2, thereby forming the mature 3′ end of 25S. Through analysis of the 27SA2 species with a prominent 3′ tail, we observed a strong stop at position 15 from the B2 site, with additional terminations detected at positions 50, 92 and 119 (Figure 7B–C). These findings indicate that Rnt1 cleaves the hairpin after positions 15 and 50 (Figure 7A). These two cleavage sites align with the digestion pattern of RNase III, which generates RNA duplex products with a characteristic 2-nt 3′-overhang, but deviate from the previously identified cleavage sites after positions 14 and 49 (9). The species terminating at position 92 corresponds to the primary terminator of Pol I (67). Moreover, the species ending at position 119 likely represents a novel terminator of Pol I, located upstream of the ‘fail-safe’ terminator at around position 250 (67). As expected (9,10), depletion of Rnt1 causes marked accumulation of 3′-extended forms of 25S precursors, particularly those extending beyond site B0 (Figure 7B, D).

Amongst all 25S precursors, the 3′ extension is longest in 27SA2, followed by 33S and 35S, and largely absent in other species (Figure 7C, D). It is seemingly puzzling that 27SA2 and 33S have a longer, less processed 3′ tail compared to their precursor 35S. The nascent pre-rRNA transcript can undergo cleavage at the A0, A1 and A2 sites during transcription (11,12). When co-transcriptional cleavage does not occur, a 35S pre-rRNA is released after B0 cleavage and then processed to 33S, 32S and 27SA2. The length of 3′ ETS extension in a pre-rRNA molecule would be inversely proportional to the time elapsed since B0 cleavage. Compared to 35S, these post-transcriptionally produced species have more time for processing and hence a shorter 3′ tail. When the A0, A1 or A2 site are co-transcriptionally cleaved, subsequent B0 cleavage would release a 33S, 32S or 27SA2 species instead, which would have a ‘fresh’ 3′ tail similar to 35S. The population of 33S, 32S and 27SA2 consists of both co- and post-transcriptionally produced species. The average length of 3′ tail of these species is determined by fractions of co- and post-transcriptional species and their lifetimes. The tail would be more processed if a species has a longer lifetime.

Our data provide biochemical evidence that majority of 27SA2 is derived from co-transcriptional processing. In addition, the 33S, which is technically difficult to examine in gel separation, is also mainly produced through co-transcriptional cleavage. The 32S has a short 3′-tail and should be produced minorly through co-transcriptional cleavage. Co-transcriptionally produced 32S species should result from cleavage at sites A1 and B0 in the absence of A2 cleavage, an event that is probably rare due to the short time duration between A1 and A2 cleavage. Hence, B0 cleavage occurs primarily after A2 and A0 cleavage.

The 35S is purely co-transcriptionally produced, but has a shorter 3′ tail than 27SA2 and 33S. This is probably because 35S has a longer lifetime than the latter species. We noticed a significant difference in the length of 3′ ETS between two independent wild-type Noc4 samples (Figure 7D). Ratios of co- and post-transcriptional processing are sensitive to cell growth conditions (11), likely accounting for the variation. The 3′ ETS tail is shortened in the *rat1*, *rrp17*, *nme1*, *mtr4* and *rrp44* mutants (Figure 7D), which may generally delay rRNA processing, leading to increased lifetime of pre-rRNAs and prolonged digestion of 3′ ETS.

How the exposed 3′ ETS after B0 cleavage is trimmed to site B2 is poorly understood, and only Rex1 has been implicated in the process (27,31). To analyze the roles of Rex1-4 in 3′ ETS processing, we individually deleted their genes (Figure 7C–D). Deletion of *REX2* or *REX3* does not alter the 3′ end processing of 27SA2 and 33S but slightly delays the 3′ end processing of 35S, suggesting they have a redundant role in 3′ ETS processing. The 35S may have a longer lifetime than 27SA2 and 33S and hence be more sensitive to the absence of a functionally redundant enzyme. Deletion of *REX1* specifically increases long processing intermediates of 27SA2, 33S and 35S ending near the B0 site. Remarkably, deletion of *REX4* causes mature 25S and all its precursors to be 3′ extended by ∼6-nt. These results support a model for 3′ ETS processing after B0 cleavage (Figure 7E). The exposed 3′ ETS is digested by Rex1 and then by Rex2 and Rex3 to position 6. As no severe defects of 25S processing are present in *rex1*, Rex1 may be partially substituted by Rex2 and Rex3. The last six nucleotides of 3′ ETS are exclusively digested by Rex4.

Processing of the 3′ ETS in wild-type yeast yields two major intermediates ending at positions 10 and 14 (Figure 7C). The two sites are occupied by only cytosines in the region (Figure 7A), suggesting that Rex1-3 distinguish against cytosine. Such substrate specificity is similar to RNase T, which also belongs to the DEDD family and plays an equivalent function in processing the 3′ end of 23S rRNA in *E. coli* (68).

Rex4 has been shown to have genetic interactions with ribosome assembly factors involved in ITS1 processing (55,69), but its exact function in pre-rRNA processing remains unclear. We demonstrate that Rex4 plays a specific role in digesting the last six nucleotides of 3′ ETS. The 3′-extended 25S rRNA appears to be fully functional since no growth defect is apparent in the *rex4* mutant (Supplementary Figure S3). Furthermore, deletion of *REX4* mildly affects multiple processing steps, resulting in a slightly increased level of 5.8SL (Figure 4A, B), accumulation of ITS2 processing intermediates of 5.8S (Figure 5C, D) and 25S (Figure 6B), and decreased 5′-extended forms of 21S (Figure 3B). Most of these defects, except for those in 3′ processing of 5.8S, are also observed in the *rnt1* mutant. These defects, linked to both LSU and SSU rRNA processing, could be potentially attributed to partial suppression of Rat1 and Rrp17. Blocked 3′ ETS processing probably delays other rRNA processing events occurring in pre-60S ribosomes and subsequently sequesters Rat1 and Rrp17.

### Polyadenylation of pre-rRNA

**Figure 8.**
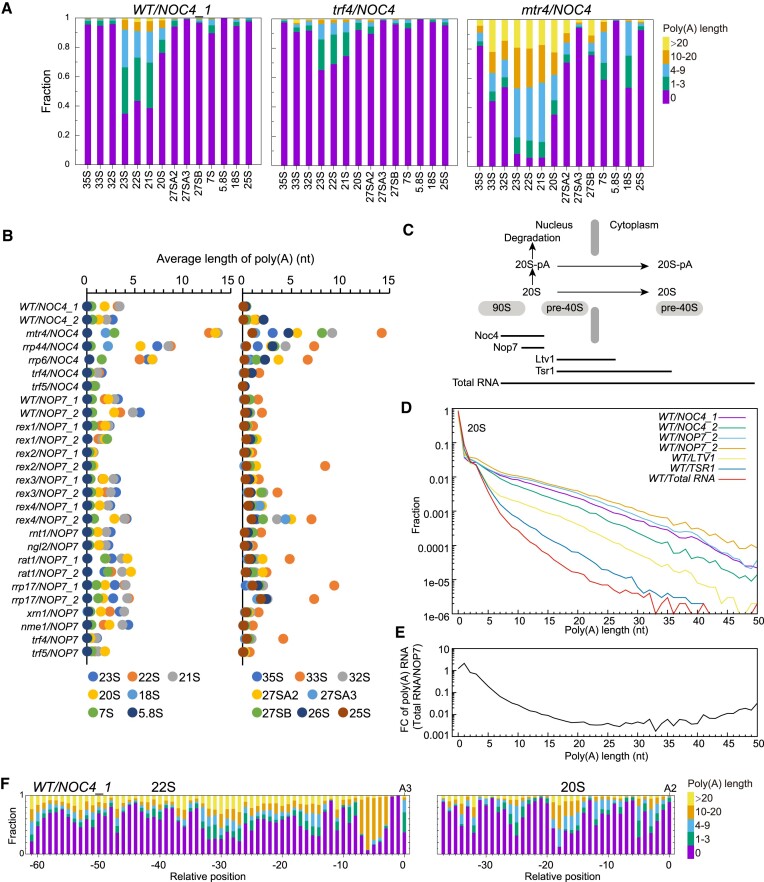
Polyadenylation of rRNA processing intermediates. (**A**) Stacked bar plots showing distribution of poly(A) length for major pre-rRNAs in the *WT*, *trf4* and *mtr4* strains. (**B**) Average length of poly(**A**) for pre-rRNAs in all analyzed strains are shown in two panels. (**C**) Fate of polyadenylated 20S pre-rRNA. Association duration of a bait protein with nuclear and cytoplasmic 20S is shown as a line. (**D**) Distribution of poly(A) length for 20S species enriched via different baits. (**E**) Fold changes of polyadenylated 20S ratios between total RNA sample and average of two *WT/NOP7* samples. (**F**) Stacked bar plots showing distribution of poly(A) length for degradation intermediates of 22S and 20S. Poly(A) length are binned into five groups: 0, 1–3, 4–9, 10–20 and >20 nt.

Aberrant processing intermediates of rRNA are polyadenylated by the TRAMP complex and degraded by the exosome (33,34). The 23S, 22S and 21S pre-rRNAs that terminate at site A3 are typical aberrant products targeted for degradation (1,41). These pre-rRNAs are indeed extensively polyadenylated, reaching 60–70% levels in wild-type yeast (Figure 8A). In contrast, the polyadenylation level is ∼20% for 20S and commonly less than 10% for other pre-rRNAs. The 33S pre-rRNA consistently shows a higher polyadenylation level than other 25S processing intermediates and is sometimes extensively polyadenylated (Figure 8B), suggesting that it is often subjected to surveillance. In addition, polyadenylation levels of pre-rRNAs are globally reduced in the *REX2* deletion for unknown reason (Figure 8B).

Majority of poly(A) sequences in pre-rRNAs are less than 10 nt in length, indicative of distributive addition of poly(A) (36). It is worth mentioning that RNAs with such short poly(A) tails would evade detection by commonly used methods that rely on oligo-dT beads for polyadenylated RNA enrichment, leading to a significant underestimation of polyadenylation level in pre-rRNAs (65).

Polyadenylation levels significantly increase for most pre-rRNAs in the absence of Mtr4, Rrp44 and Rrp6 (Figure 8A–B), consistent with previous reports (36,37,65,70). Compared with *mtr4*, there is a lesser increase of polyadenylation in *rrp44* and *rrp6*, indicating that Rrp44 and Rrp6 are partially redundant in degrading polyadenylated pre-rRNAs. On the other hand, deletion of *TRF4* or *TRF5* globally reduces polyadenylation of pre-rRNAs (Figure 8A, B), as expected (36,65).

The 20S pre-rRNA is generated in 90S preribosomes in the nucleus and exported in pre-40S ribosomes to the cytoplasm for final maturation (Figure 8C). As the pre-rRNA surveillance system operates exclusively in the nucleus, polyadenylated 20S species present in the cytoplasm are those that escape nuclear degradation. Subsequently, cytoplasmic 20S would be less polyadenylated compared to nuclear 20S. By comparing the polyadenylation level of nuclear and cytoplasmic 20S, we could estimate the degradation efficiency of polyadenylated 20S in the nucleus.

Nuclear and cytoplasmic 20S in varying ratios can be purified with different bait proteins (Figure 8C, D). The 20S species associated with Noc4 and Nop7 are entirely nuclear and therefore possess the highest levels of polyadenylation. Additionally, Nop7-associated 20S consistently exhibits higher polyadenylation levels compared to Noc4-associated 20S, suggesting that Nop7, a component of pre-60S, tends to associate with late stage 90S preribosomes where the 20S has been more extensively polyadenylated. Both Ltv1 and Tsr1 assemble into pre-40S ribosomes in the nucleus, but Ltv1 is released in the cytoplasm earlier than Tsr1 (71,72). Accordingly, Ltv1-bound 20S has a polyadenylation level intermediate between that of nuclear and Tsr1-bound 20S. The 20S from total RNA displays the lowest level of polyadenylation, reflecting that the 20S is predominantly located in the cytoplasm.

To estimate the nuclear degradation efficiency of polyadenylated 20S, we calculated the degree to which polyadenylated 20S is depleted in total RNA (as an approximation of the cytoplasmic form) compared to Nop7-associated RNA (as the nuclear form) as a function of poly(A) length (Figure 8E). The 20S species with a 3-A, 6-A and 14-A tail is reduced by 31%, 89% and 99%, respectively. The degree of reduction is nearly exponentially proportional to the poly(A) length up to ∼20 nt and fluctuates at > 99% levels for longer tails. Hence, by monitoring the fate of polyadenylated 20S, we determined the quantitative relationship between degradation-stimulating activity of the poly(A) tail and its length.

The 20S, 21S, 22S and 23S pre-rRNAs contain an exact 3′ end that does not undergo further exonucleolytic processing. Any intermediates of these pre-rRNAs with a shortened 3′ end are resulted from partial degradation. Interestingly, degradation intermediates of 20–23S often carry a longer poly(A) tail compared to pre-rRNAs with an intact 3′ end (Figure 8F). Similar polyadenylation patterns are also present for pre-rRNAs ending at site B2 (Supplementary Figure S4-S31I). This suggests that degradation intermediates of pre-rRNA are resilient molecules that resist complete digestion by the exosome and are subsequently released and re-polyadenylated over an extended period.

In summary, our study reveals the quantitative profile of polyadenylation in pre-rRNAs by measuring the poly(A) tail for individual RNA molecules. We have determined the dependence of degradation efficiency of polyadenylated 20S on the poly(A) length. Additionally, we show that degradation intermediates of pre-rRNA, once released from the exosome, are often re-polyadenylated more extensively than initial substrates. Although these findings were observed for specific pre-rRNAs, they likely reflect intrinsic characteristics of the nuclear RNA surveillance system.

## Conclusion

We have enriched pre-rRNAs with preribosome purification and profiled them with CircTA-seq. This sensitive and quantitative approach enabled us to determine precise processing sites of rRNA, capture low-abundance intermediates produced during exonucleolytic processing and identify substrates for even functionally redundant nucleases. Through extensive mutational analysis, we proposed a unified pathway for 5′ end processing of 5.8S, uncovered the initial steps in 7S processing and determined the pathway for 3′ end processing of 25S since B0 cleavage (Figure 1). Our study reveals that the length of 3′ ETS can serve as a sensitive monitor of pre-rRNA age, distinguishing co- and post-transcriptionally produced species. We identified novel intermediates during 5′ degradation of 22S and 33S pre-rRNAs. We delineated specific functions of four Rex proteins in rRNA processing. With accurate measurement of polyadenylation levels, we show that the degradation efficiency of 20S pre-rRNA is critically dependent on its poly(A) length. Moreover, we found that degradation intermediates can be released from the exosome and extensively re-polyadenylated. These findings significantly enhance our mechanistic understanding of 5.8S and 25S rRNA processing and pre-rRNA surveillance.

## Supplementary Material

gkae606_Supplemental_File

## Data Availability

The data underlying this article are available in the article and in its online supplementary material. The raw data of CircTA-seq are available in the National Genomics Data Center [bigd.big.ac.cn] under the GSA accession code CRA017081.
