# Supplementary material for: High resolution landscape of ribosomal RNA processing and surveillance
Source: Nucleic Acids Res. 2024 Jul 12;52(17):10630–44. doi: 10.1093/nar/gkae606 (PMC11417381; doi:10.1093/nar/gkae606)

High resolution landscape of ribosomal RNA processing and surveillance

Weidong An[1], Yunxiao Yan[1,2] and Keqiong Ye[1,2]*

[1]Key Laboratory of RNA Science and Engineering, CAS Center for Excellence in Biomacromolecules,

Institute of Biophysics, Chinese Academy of Sciences, Beijing 100101, China.

[2]University of Chinese Academy of Sciences, Beijing 100049, China.

Supplementary Materials

Table S1-S3
Figure S1-S31

Table S1. Yeast strains

Strain	Genotype	Origin
BY4741	*MATa, his3Δ1, leu2Δ0, met15Δ0, ura3Δ0*	Euroscarf
NOC4-TAP	BY4741, *NOC4-TAP::HIS3MX6*	Open Biosystems
NOP7-TAP	BY4741, *NOP7-TAP::HIS3MX6*	Open Biosystems
LTV1-TAP	BY4741, *LTV1-TAP::HIS3MX6*	Open Biosystems
TSR1-TAP	BY4741, *TSR1-TAP::HIS3MX6*	Open Biosystems
rrp6Δ/NOC4-TAP	BY4741, *NOC4-TAP::HIS3MX6, rrp6Δ::natNT2*	This study
GAL::rrp44/NOC4-TAP	BY4741, *NOC4-TAP::HIS3MX6, natNT2-pGALL::3HA-rrp44*	This study
GAL::mtr4/NOC4-TAP	BY4741, *NOC4-TAP::HIS3MX6, natNT2-pGALL::3HA-mtr4*	Sun et al, 2019 Elife
trf4Δ/NOC4-TAP	BY4741, *NOC4-TAP::HIS3MX6, trf4Δ::natNT2*	This study
trf5Δ/NOC4-TAP	BY4741, *NOC4-TAP::HIS3MX6, trf5Δ::natNT2*	This study
GAL::nme1/NOP7-TAP	BY4741, *NOP7-TAP::HIS3MX6, natNT2-pGALL:: nme1*	This study
GAL::rnt1/NOP7-TAP	BY4741, *NOP7-TAP::HIS3MX6, natNT2-pGALL:: rnt1*	This study
trf4Δ/NOP7-TAP	BY4741, *NOP7-TAP::HIS3MX6, trf4Δ::natNT2*	This study
trf5Δ/NOP7-TAP	BY4741, *NOP7-TAP::HIS3MX6, trf5Δ::natNT2*	This study
GAL::rat1/NOP7-TAP	BY4741, *NOP7-TAP::HIS3MX6, natNT2-pGALL::3HA-rat1*	This study
GAL::rrp17/NOP7-TAP	BY4741, *NOP7-TAP::HIS3MX6, natNT2-pGALL::3HA-rrp17*	This study
xrn1Δ/NOP7-TAP	BY4741, *NOP7-TAP::HIS3MX6, xrn1Δ::natNT2*	This study
rex1Δ/NOP7-TAP	BY4741, *NOP7-TAP::HIS3MX6, rex1Δ::natNT2*	This study
rex2Δ/NOP7-TAP	BY4741, *NOP7-TAP::HIS3MX6, rex2Δ::natNT2*	This study
rex3Δ/NOP7-TAP	BY4741, *NOP7-TAP::HIS3MX6, rex3Δ::natNT2*	This study
rex4Δ/NOP7-TAP	BY4741, *NOP7-TAP::HIS3MX6, rex4Δ::natNT2*	This study
ngl2Δ/NOP7-TAP	BY4741, *NOP7-TAP::HIS3MX6, ngl2Δ::natNT2*	This study

Table S2. Primers for CircTA-seq

Primer	Sequence (5' to 3')
f1	CCTTGGCACCCGAGAATTCCA TCGAACTTGTCTTCAACTGCT
f2	CCTTGGCACCCGAGAATTCCA GACTACTGGCAGGATCAACC
f3	CCTTGGCACCCGAGAATTCCA GAACCAAGAGATCCGTTGTTG
f4	CCTTGGCACCCGAGAATTCCA GGTACTCCTACCTGATTTGAGG
b1	ACACGACGCTCTTCCGATCT GGTGAACCTGCGGAAGG
b2	ACACGACGCTCTTCCGATCT CGGTGAGAGATTTCTGTGCT
b3	ACACGACGCTCTTCCGATCT GGGGCATGCCTGTTTGAGC
b4	ACACGACGCTCTTCCGATCT GCTGAGATTAAGCCTTTGTTGTC
RT18S	GACTACTGGCAGGATCAACCAG
RT25S	CGGGTACTCCTACCTGATTTGAGG
RT58S	GAGAACCAAGAGATCCGTTGTT
P5	CAAGCAGAAGACGGCATACGAGAT[6-nt index]GTGACTGGAGTTCCTTGGCACCCGAGAATTCCA
P7	AATGATACGGCGACCACCGAGATCTACAC[5-nt index]ACACTCTTTCCCTACACGACGCTCTTCCGATCT

Table S3. Definition of pre-rRNA species in CircTA-seq analysis.

pre-rRNA	Exact 5' end	Range of 5' end	Exact 3' end	Range of 3' end
35S	1	[1, A0-10)	B2	[B2-50, end+1)
33S	A0	[A0-10, A1-10)	B2	[B2-50, end+1)
32S	A1	[A1-10, A1+50)	B2	[B2-50, end+1)
23S	1	[1, A0-10)	A3	[A2+10, A3+10)
22S	A0	[A0-10, A1-10)	A3	[A2+10, A3+10)
21S	A1	[A1-10, A1+50)	A3	[A2+10, A3+10)
20S	A1	[A1-10, A1+50)	A2	[D-50, A2+10)
18S	A1	[A1-10, A1+50)	D	[D-50, D+10)
27SA2	A2	[A2-10, A3-10)	B2	[B2-50, end+1)
27SA3	A3	[A3-10, A3+10)	B2	[B2-50, end+1)
27SB	B1	[A3+10, B1+50)	B2	[B2-50, end+1)
26S	C2	[C2-10, C1-20)	B2	[B2-50, end+1)
25S	C1	[C1-20, C1+50)	B2	[B2-50, end+1)
7S	B1	[A3+10, B1+50)	C2	[E+50, C2+10)
5.8S	B1	[A3+10, B1+50)	E	[E-50, E+50)
UNK	All other species			

The coordinate system ranges from 1 for the first residue to 6858 for the last residue (end). A processing site refers to the coordinate of its 3' nucleotide. Each range is shown in the half-open format, with the start position inclusive and the end position not inclusive.

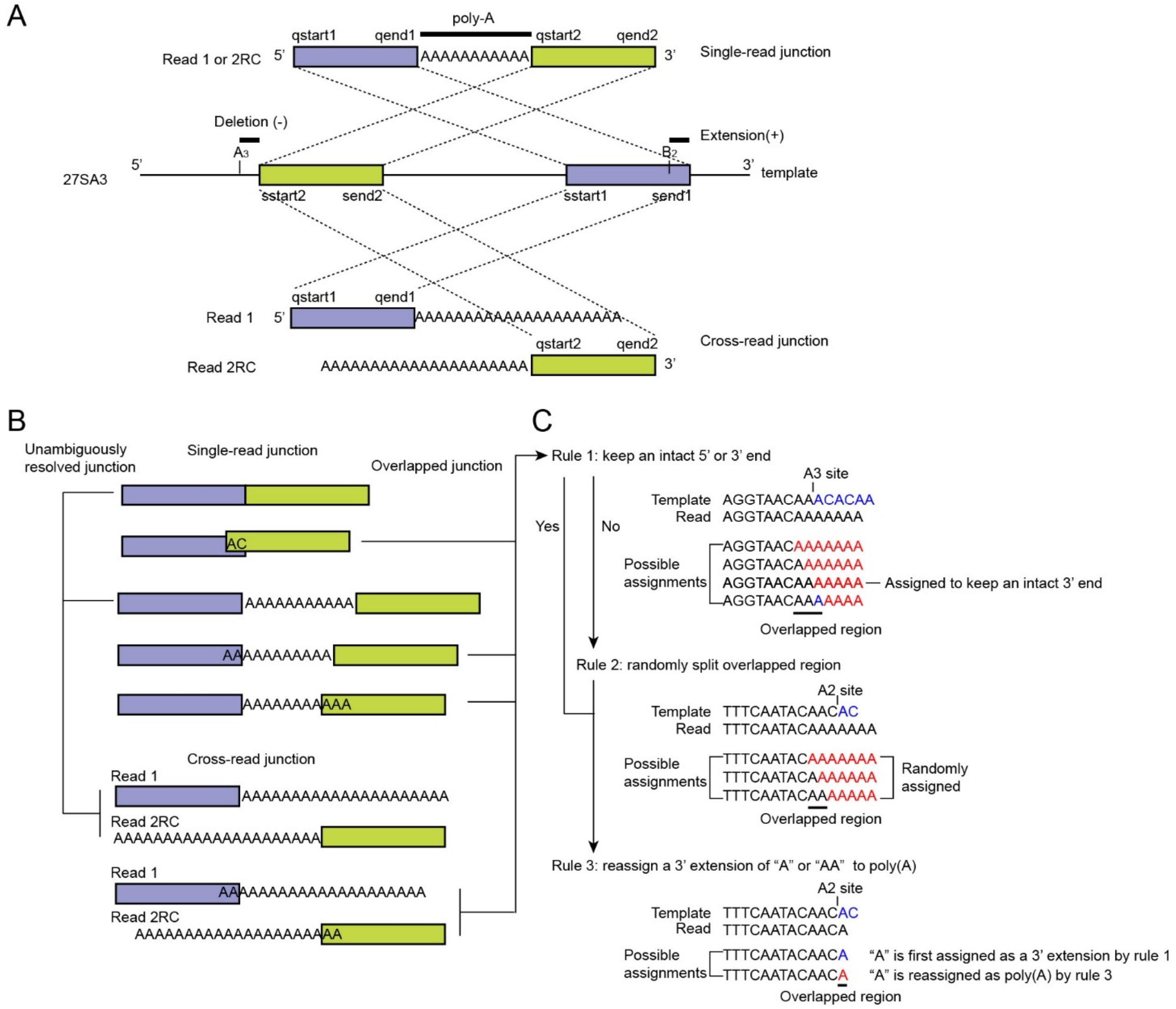

Figure S1. Processing of CircTA-seq data.

(A) Mapping of junction sequences of circularized RNA to the rDNA sequence. A single-read junction and a cross-read junction are shown on the top and bottom of the reference sequence. Illustrated is a 27SA3 species. For a short single-read junction, the read consists of the 3' end region (blue box) of RNA, an optional poly(A) region and the 5' end region (green box) of RNA. For a cross-read junction, the 5' and 3' ends of RNA are located in read 1 and 2. Read 2 is converted to its reverse complementary sequence (2RC). Deletion and extension in an end are determined relative to a known processing site (A3 and B2 in the example). (B) Types of overlapped junctions. (C) Overlapped regions in circularized junctions are resolved by sequentially applying rules 1 to 3. An example is illustrated for each rule. Sequences at the 5' and 3' side of a processing site are colored black and blue, respectively. Appended poly(A) sequences are colored in red. Overlapped regions are underlined.

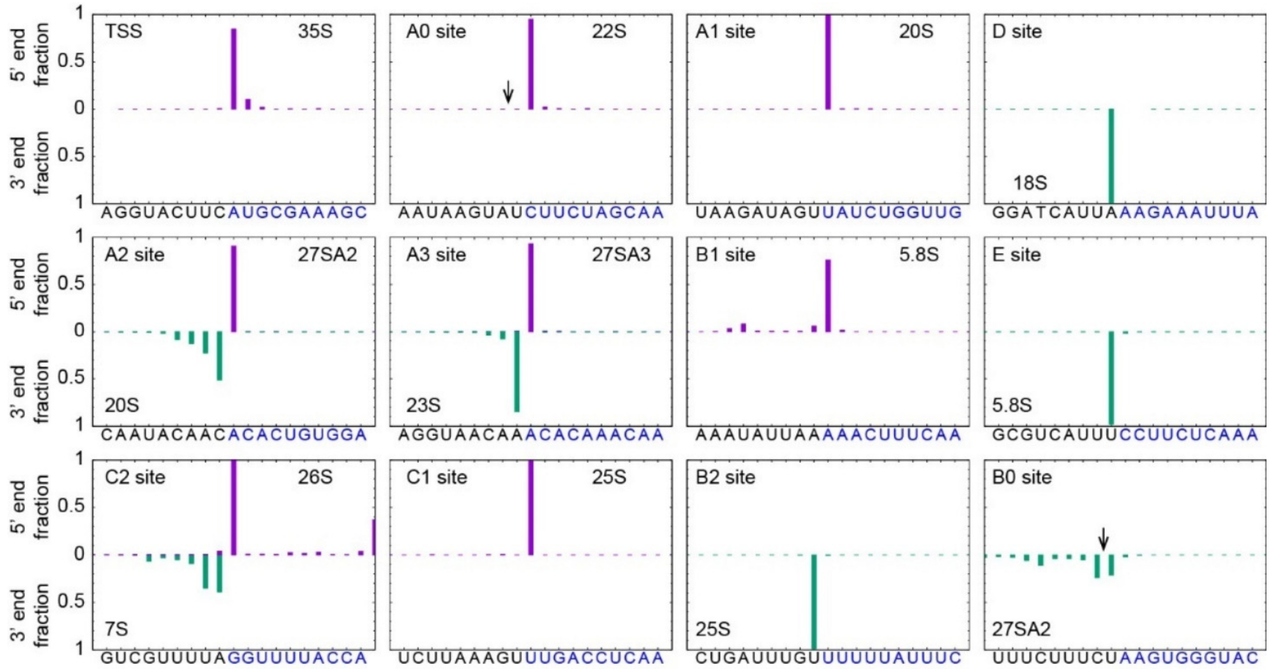

Figure S2. Histograms showing distribution of pre-rRNA ends around major processing sites. Distribution of 5' and 3' ends are displayed above and below the x-axis, respectively. Both 5' and 3' products resulting from endonucleolytic cleavage at sites A2, A3 and C2 are illustrated. RNA sequences at the 5' and 3' side of a processing site are colored in black and blue, respectively. End counts at each position are normalized to total reads of a specific pre-rRNA, but 5' end counts of 26S processing intermediates are normalized to the count at the C2 end. The 5.8S, 18S and 25S rRNAs are derived from the total RNA sample, the 7S and 26S from the *WT/NOP7_1* sample and all other species from the *WT/NOC4_1* sample. Arrows indicate the previously defined processing sites that differ from our data.

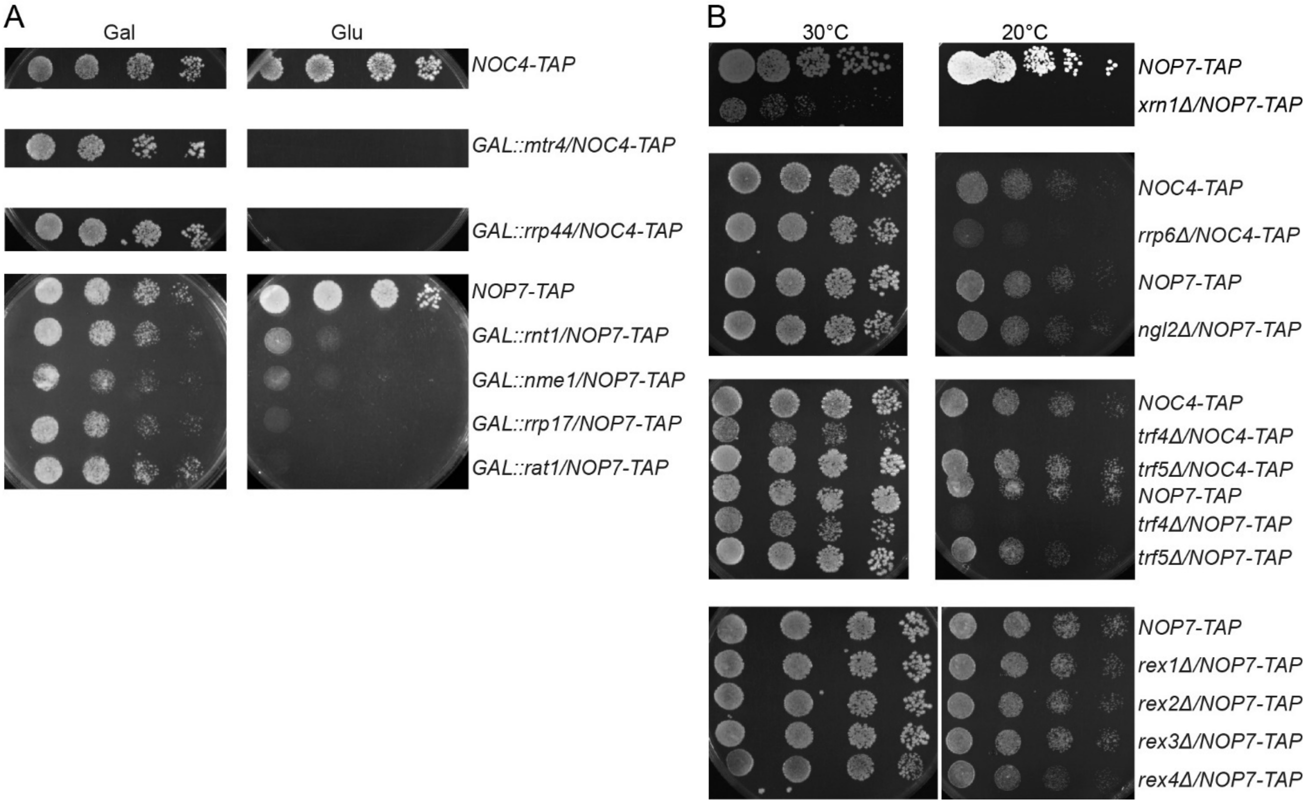

Figure S3. Growth assays.

(A) Growth assays of *GAL* conditional expression strains. Five-fold serial dilutions of cells (OD_{600}=0.2) were grown on YPG (galactose) or YPD (glucose) medium at 30 °C.

(B) Growth assays of gene deletion strains. Five-fold serial dilutions of cells (OD_{600}=0.2) were grown on YPD medium at 30 °C or 20 °C.

Figures S4-S31. Summary of CircTA-seq analysis for 28 samples.
Figure S4. CircTA-seq analysis of *WT* total RNA.
Figure S5. CircTA-seq analysis of *WT NOC4* rep1.
Figure S6. CircTA-seq analysis of *WT NOC4* rep2.
Figure S7. CircTA-seq analysis of *mtr4 NOC4* .
Figure S8. CircTA-seq analysis of *rrp44 NOC4* .
Figure S9. CircTA-seq analysis of *rrp6 NOC4* .
Figure S10. CircTA-seq analysis of *trf4 NOC4* .
Figure S11. CircTA-seq analysis of *trf5 NOC4*.
Figure S12. CircTA-seq analysis of *WT NOP7* rep1.
Figure S13. CircTA-seq analysis of *WT NOP7* rep2.
Figure S14. CircTA-seq analysis of *rex1 NOP7* rep1.
Figure S15. CircTA-seq analysis of *rex1 NOP7* rep2.
Figure S16. CircTA-seq analysis of *rex2 NOP7* rep1.
Figure S17. CircTA-seq analysis of *rex2 NOP7* rep2.
Figure S18. CircTA-seq analysis of *rex3 NOP7* rep1.
Figure S19. CircTA-seq analysis of *rex3 NOP7* rep2.
Figure S20. CircTA-seq analysis of *rex4 NOP7* rep1.
Figure S21. CircTA-seq analysis of *rex4 NOP7* rep2.
Figure S22. CircTA-seq analysis of *rnt1 NOP7*.
Figure S23. CircTA-seq analysis of *ngl2 NOP7*.
Figure S24. CircTA-seq analysis of *rat1 NOP7* rep1.
Figure S25. CircTA-seq analysis of *rat1 NOP7* rep2.
Figure S26. CircTA-seq analysis of *rrp17 NOP7* rep1.
Figure S27. CircTA-seq analysis of *rrp17 NOP7* rep2.
Figure S28. CircTA-seq analysis of *xrn1 NOP7*.
Figure S29. CircTA-seq analysis of *nme1 NOP7*.
Figure S30. CircTA-seq analysis of *trf4 NOP7*.
Figure S31. CircTA-seq analysis of *trf5 NOP7*.

(A) 2D plot of rRNA processing intermediates. Each dot represents an RNA species with its 5' and 3' end positions in 35S pre-rRNA as x- and y-coordinates and is color coded by RNA abundance. Panels for 35S, 33S-32S, 27S, 26S-25S, 23S, 22S-18S and 7S-5.8S are based on primer pairs f1/b4, f2/f4, f3/b4, f4/b4, f1/b2, f2/b1 and f3/b3, respectively. A total of 100,000 reads are analyzed for each dataset. Species with at least 6 reads are displayed.
(B) Distribution of 5' end of 32S, 21S, 20S and 18S species.
(C) Distribution of 5' end of 27S processing intermediates in log scale. The 27SA2, 27SA3 and 27SB are all detected by the same primer pair f3/b4 and normalized together.
(D) Distribution of 5' end of 27SB, 7S and 5.8S species.
(E) Distribution of 3' end of 7S processing intermediates in log scale.
(F) Distribution of 5' end of 26S processing intermediates in log scale.
(G) Distribution of 3' end of 25S processing intermediates.
(H) Stacked bar plot showing the distribution of poly(A) length for major rRNA processing intermediates. Poly(A) length is divided into 5 classes of 0, 1-3, 4-9, 10-20 and >21 nt, which are colored in purple, green,

cyan, orange and yellow, respectively.

(I) Stacked bar plots showing the distribution of poly(A) length of pre-rRNAs with a specific 3' end. The relative position to a processing site (at position 0), base and number of reads are labeled for each position. Bars are shown for 3' ends supported by at least 20 reads. The 5.8S and 7S panels together constitute the entire 3' tail of 7S.

Fig S4. WT Total RNA

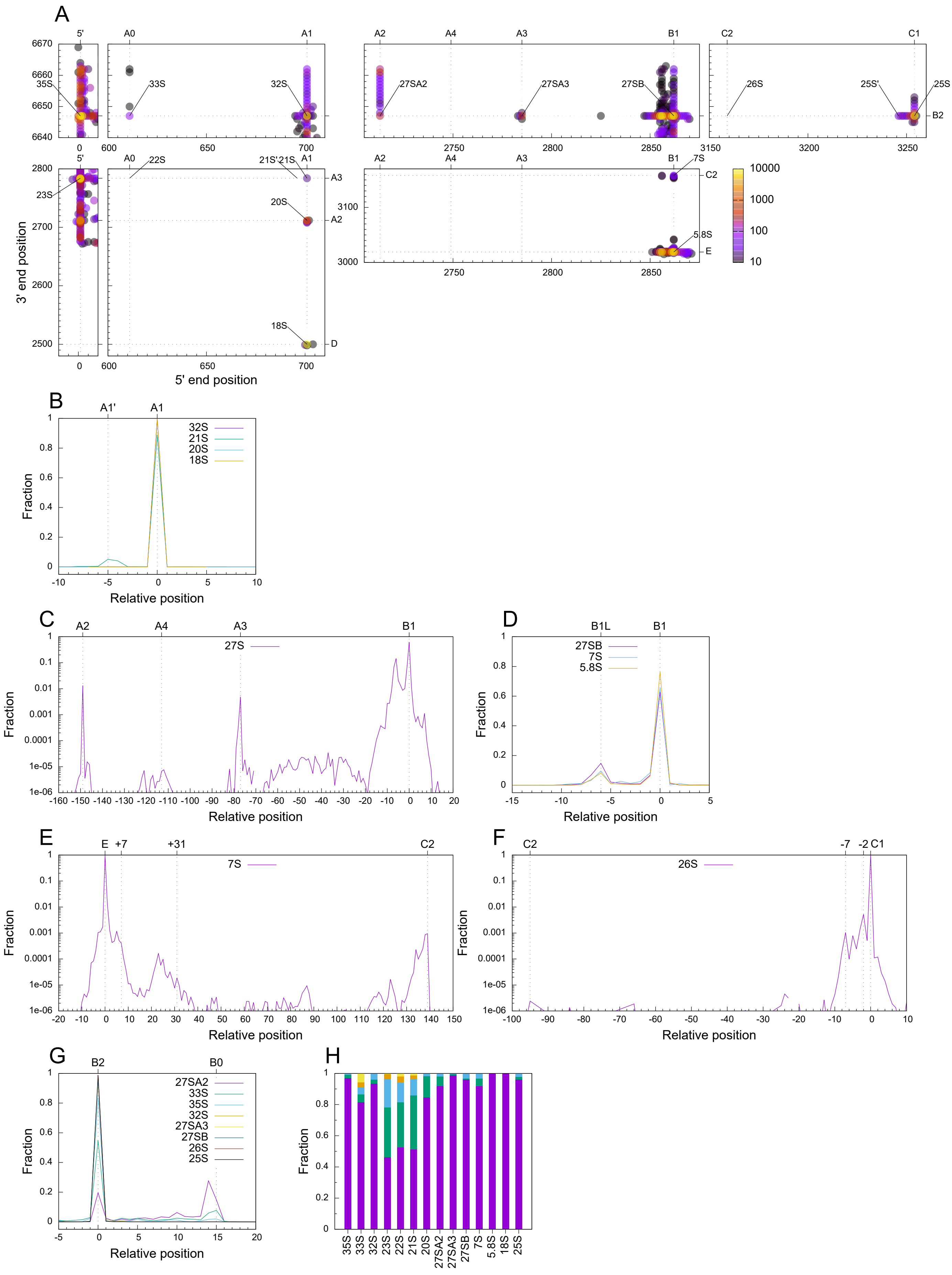

Fig S4. WT Total RNA

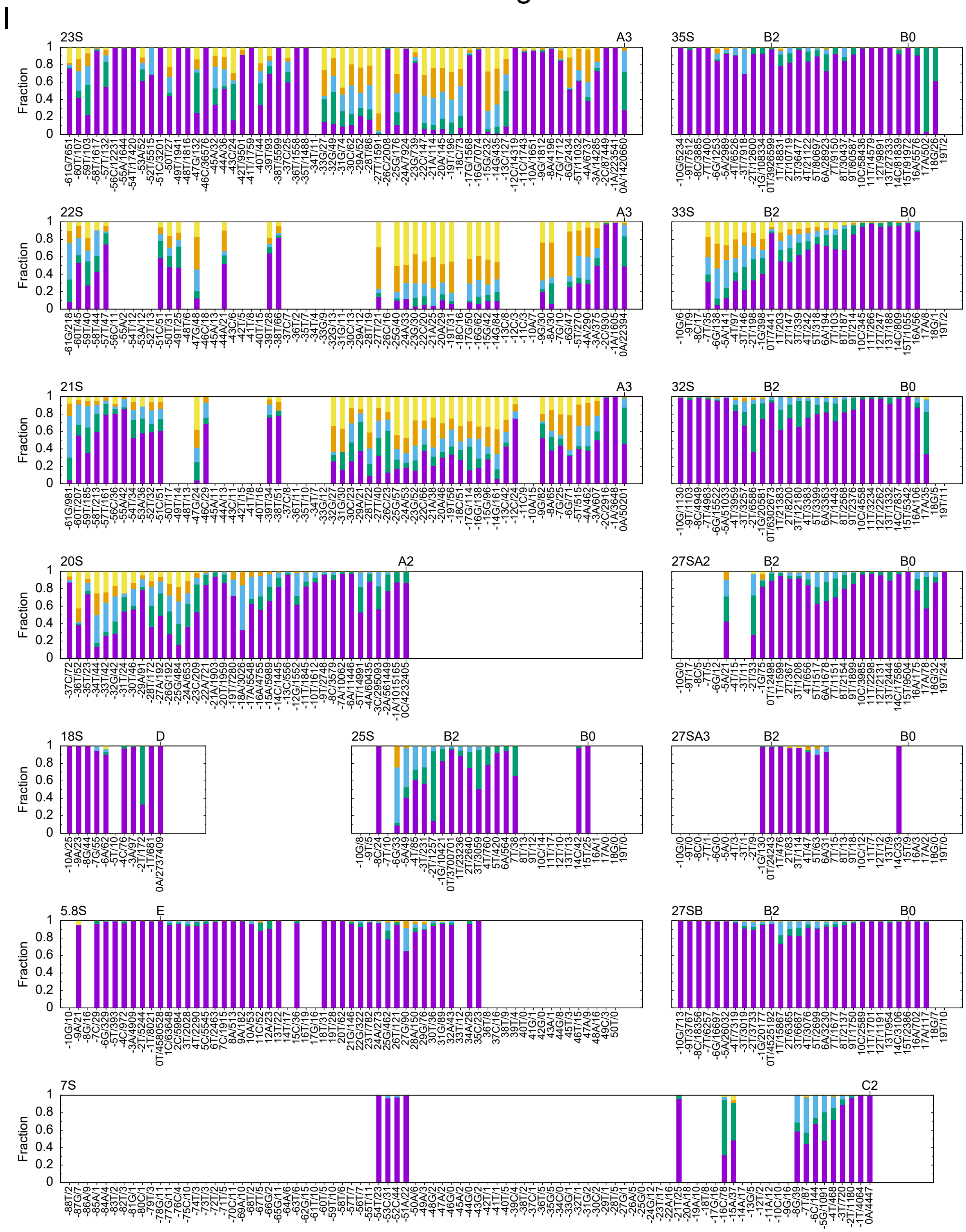

Fig S5. WT NOC4 rep1

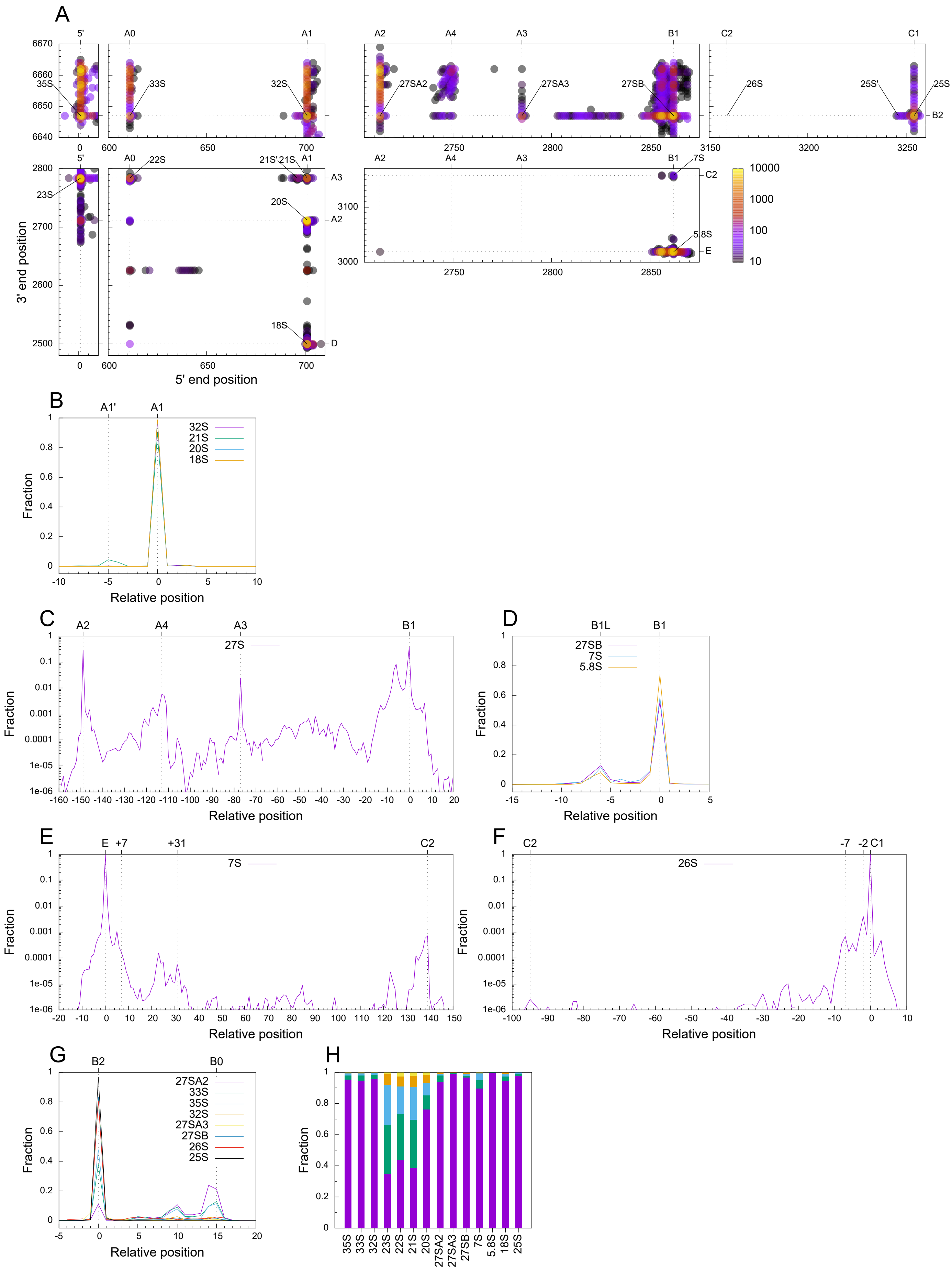

Fig S5. WT NOC4 rep1

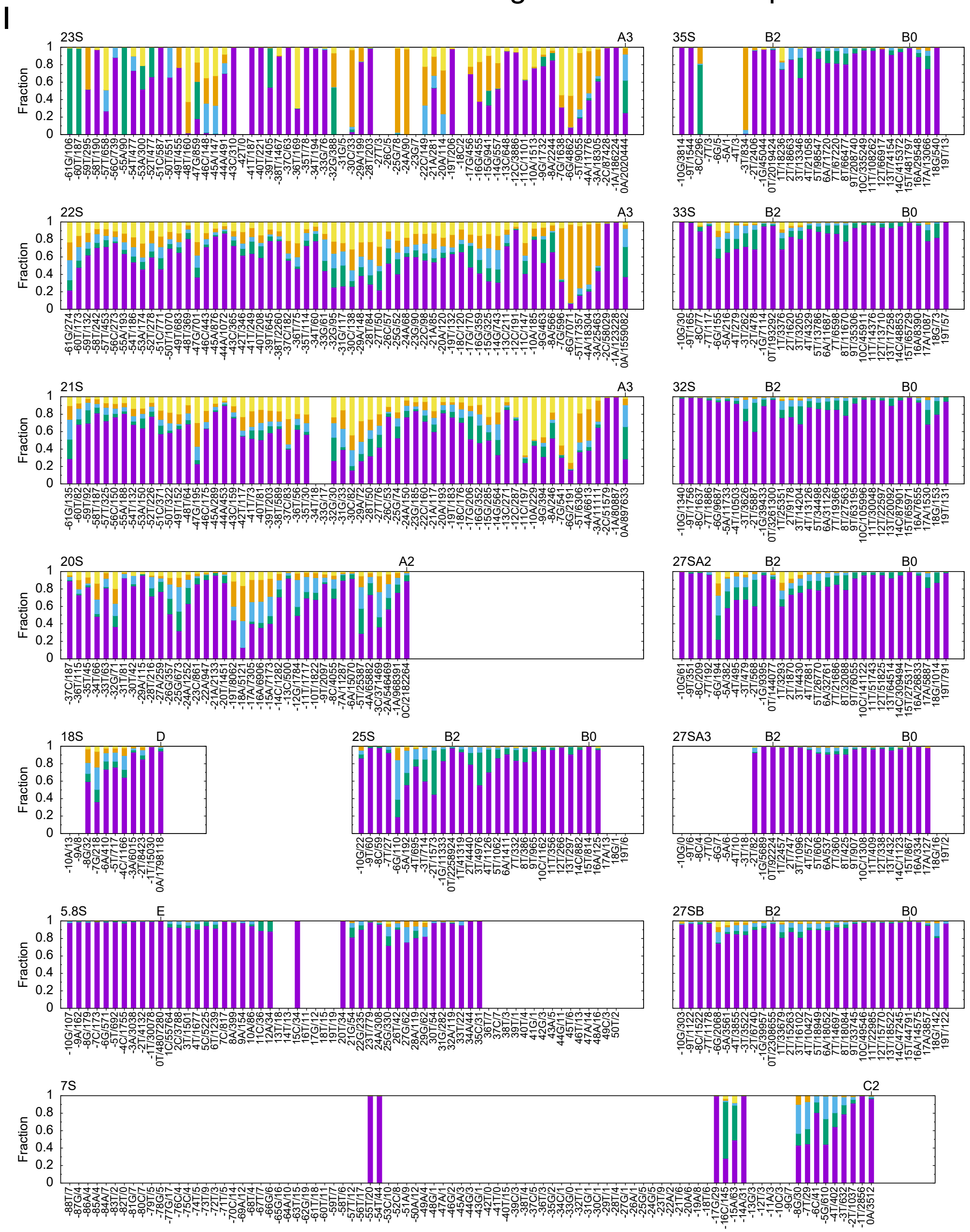

Fig S6. WT NOC4 rep2

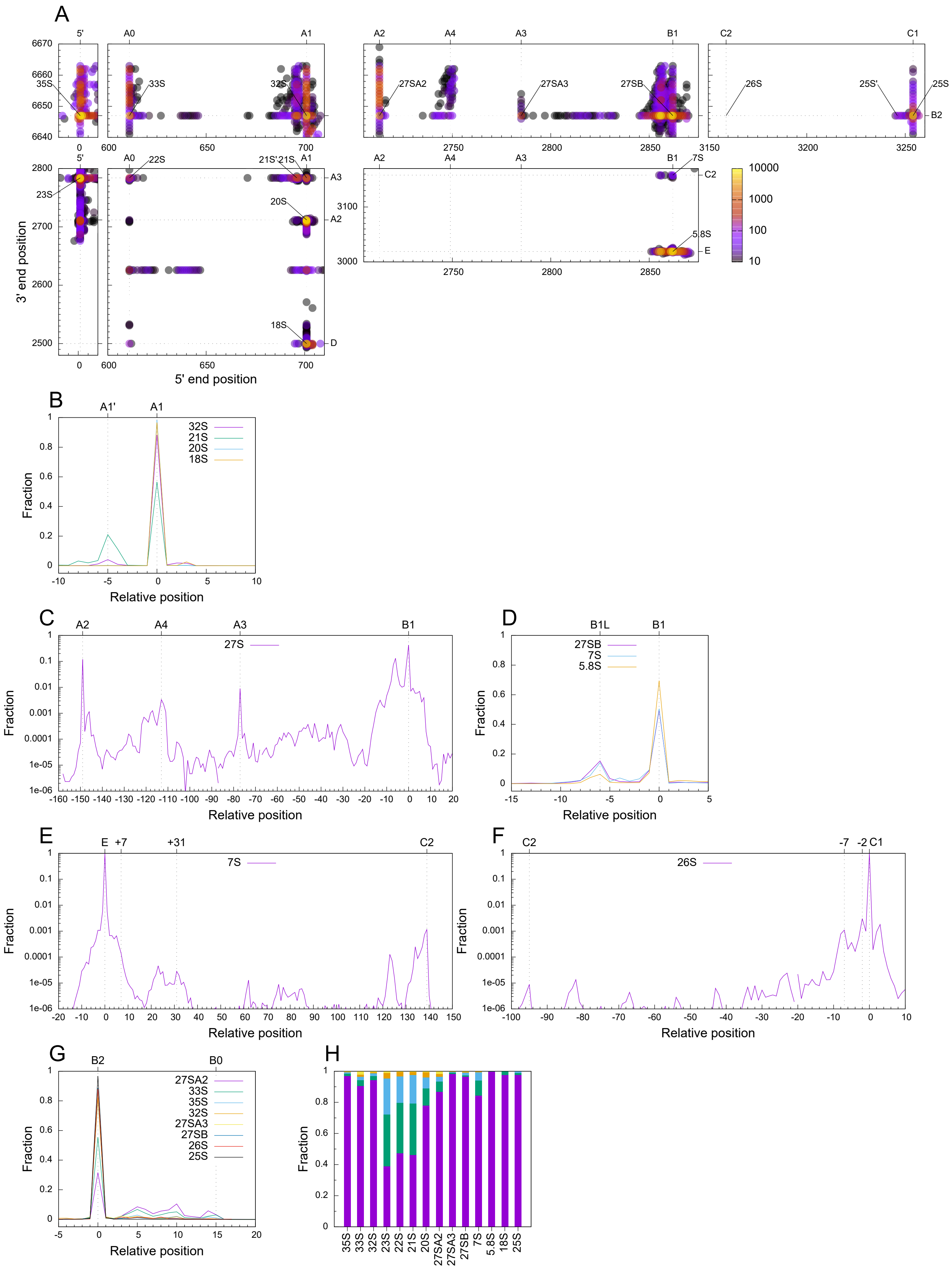

Fig S6. WT NOC4 rep2

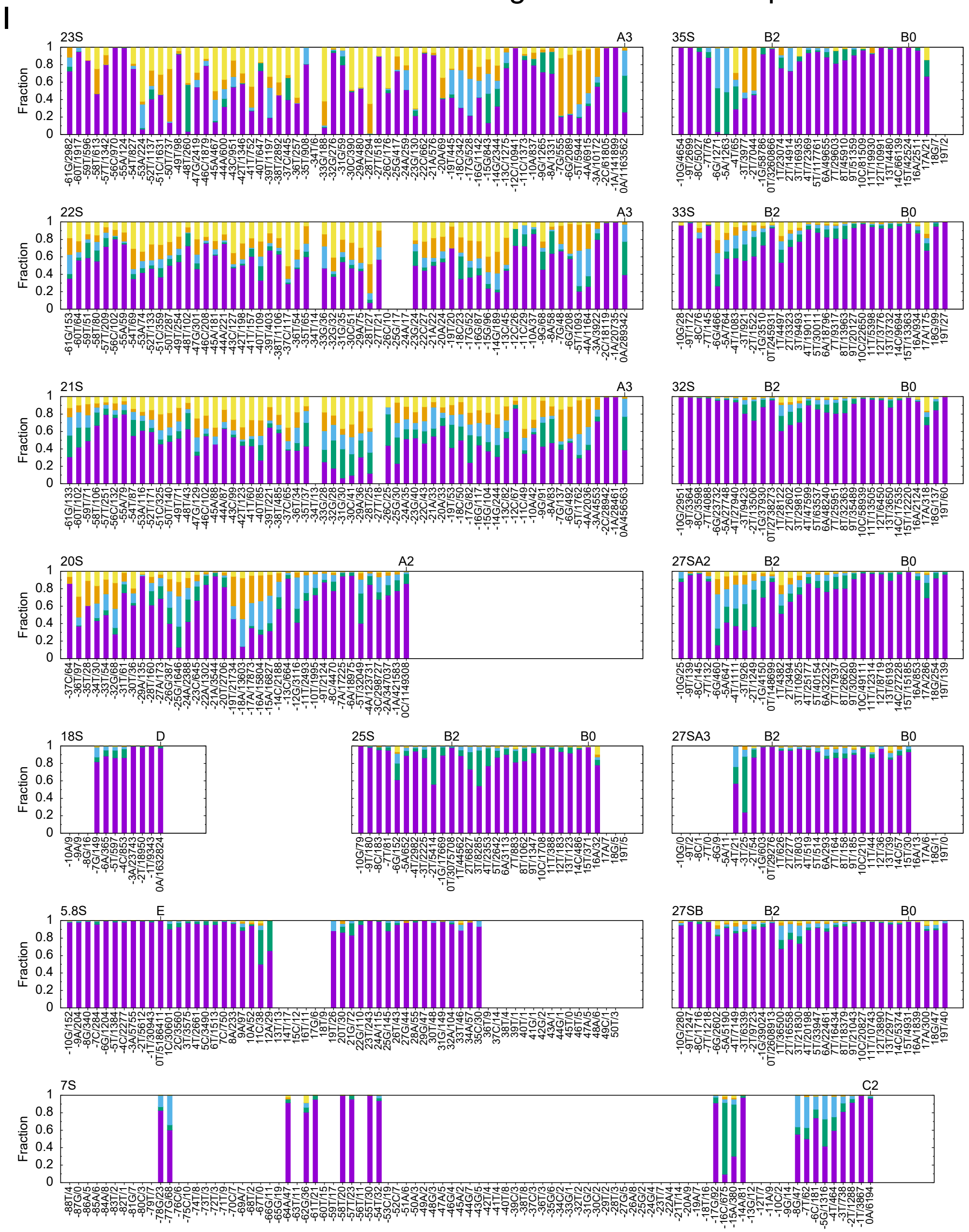

Fig S7. mtr4 NOC4

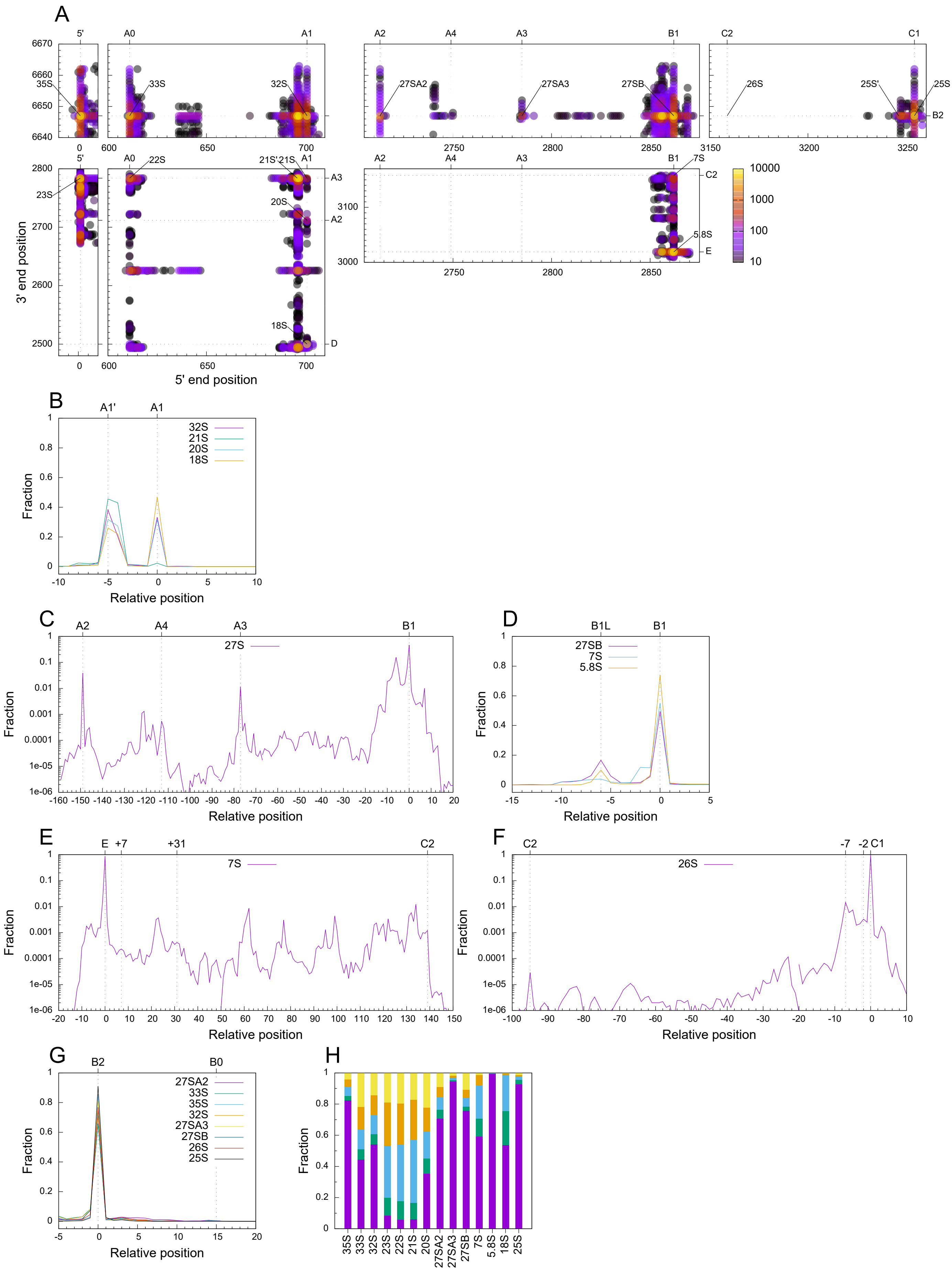

Fig S7. mtr4 NOC4

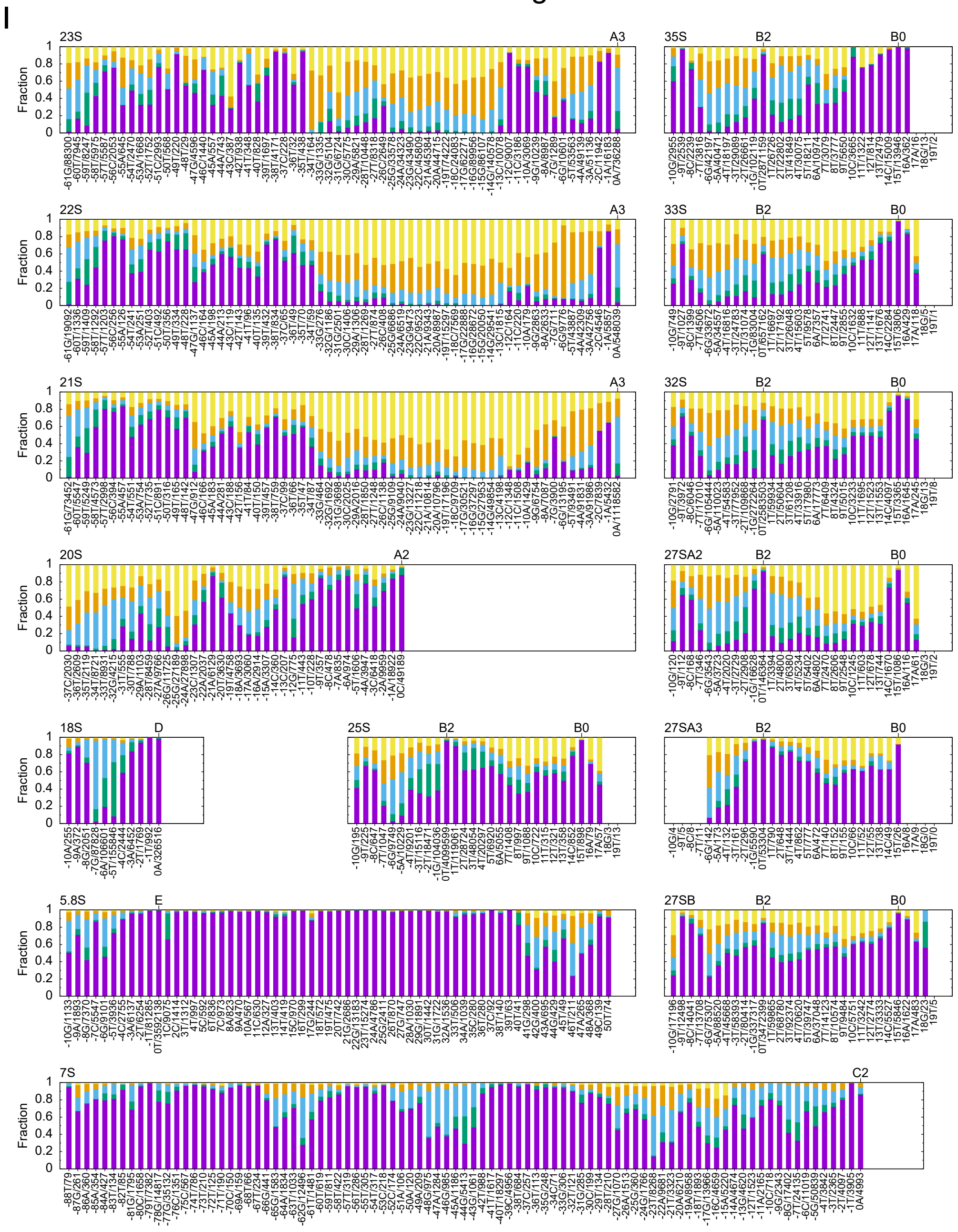

Fig S8. rrp44 NOC4

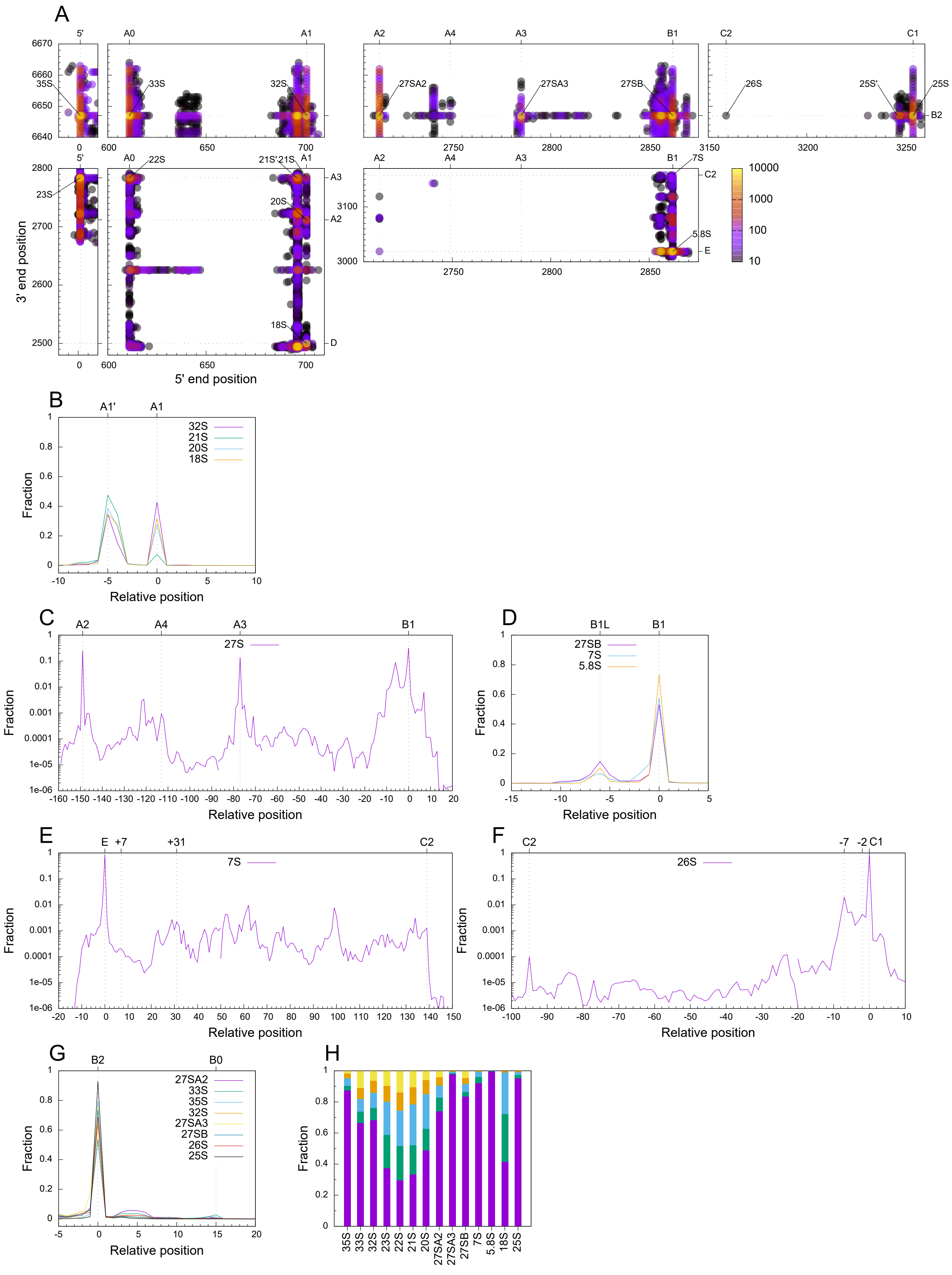

Fig S8. rrp44 NOC4

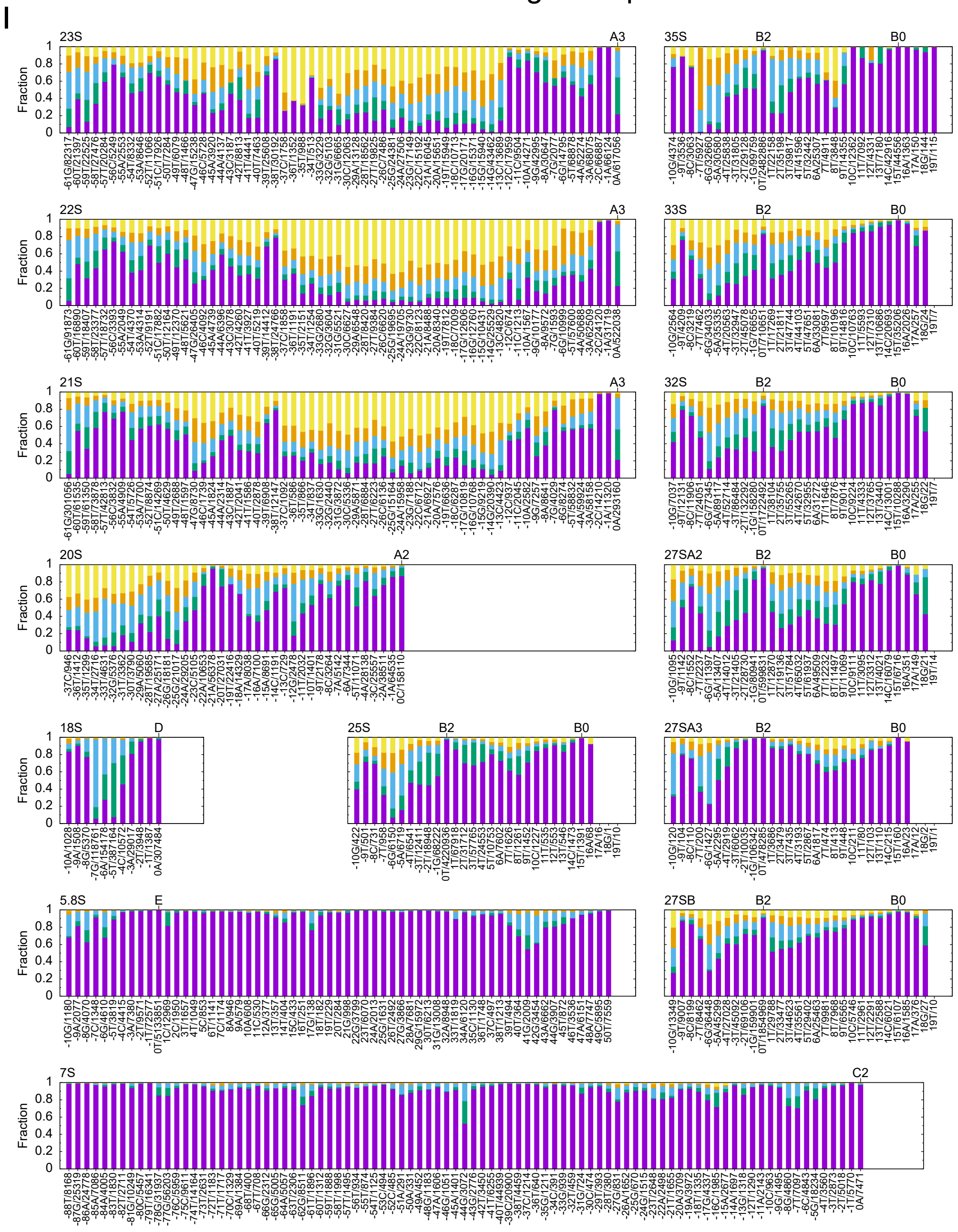

Fig S9. rrp6 NOC4

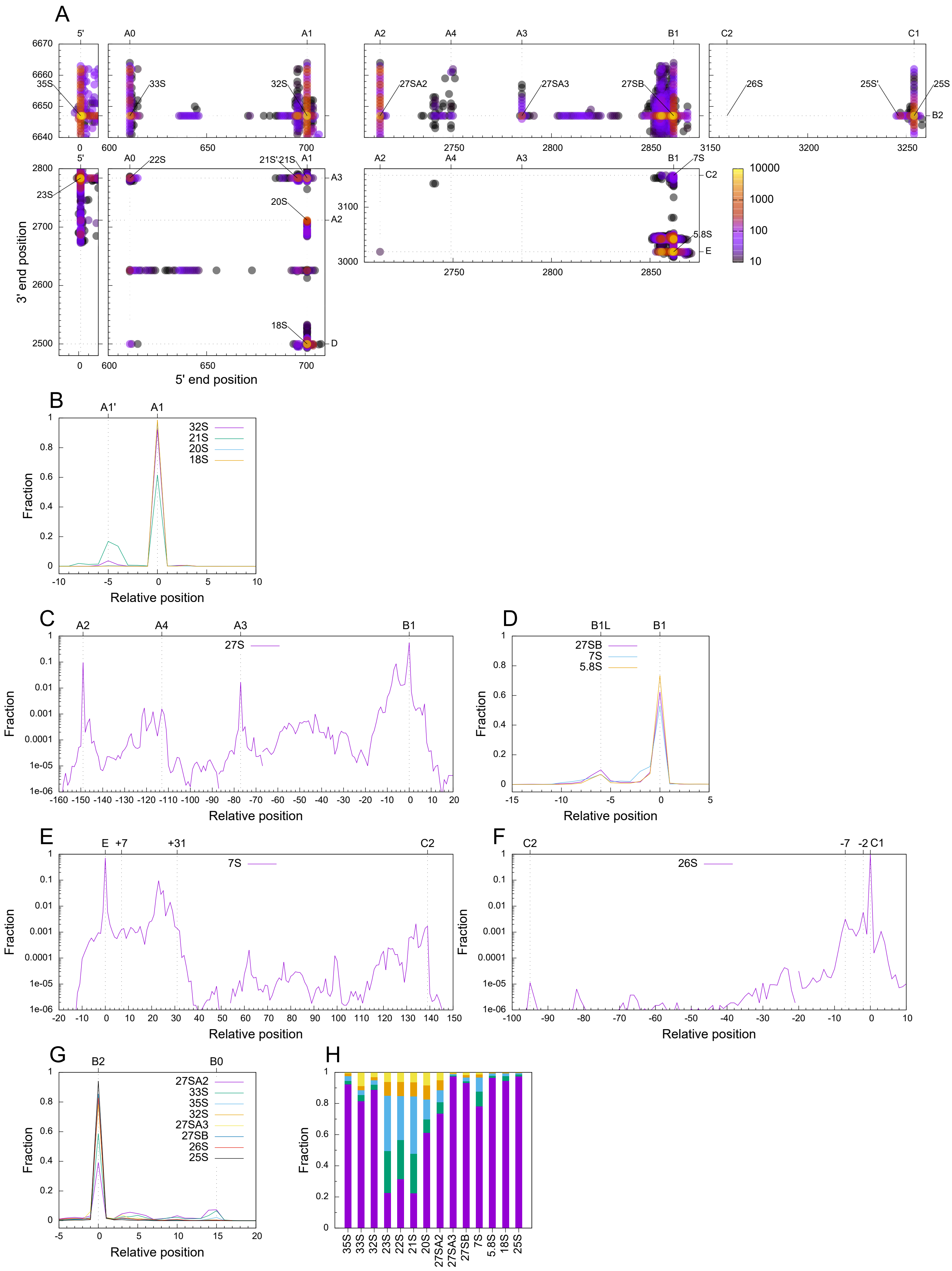

Fig S9. rrp6 NOC4

I

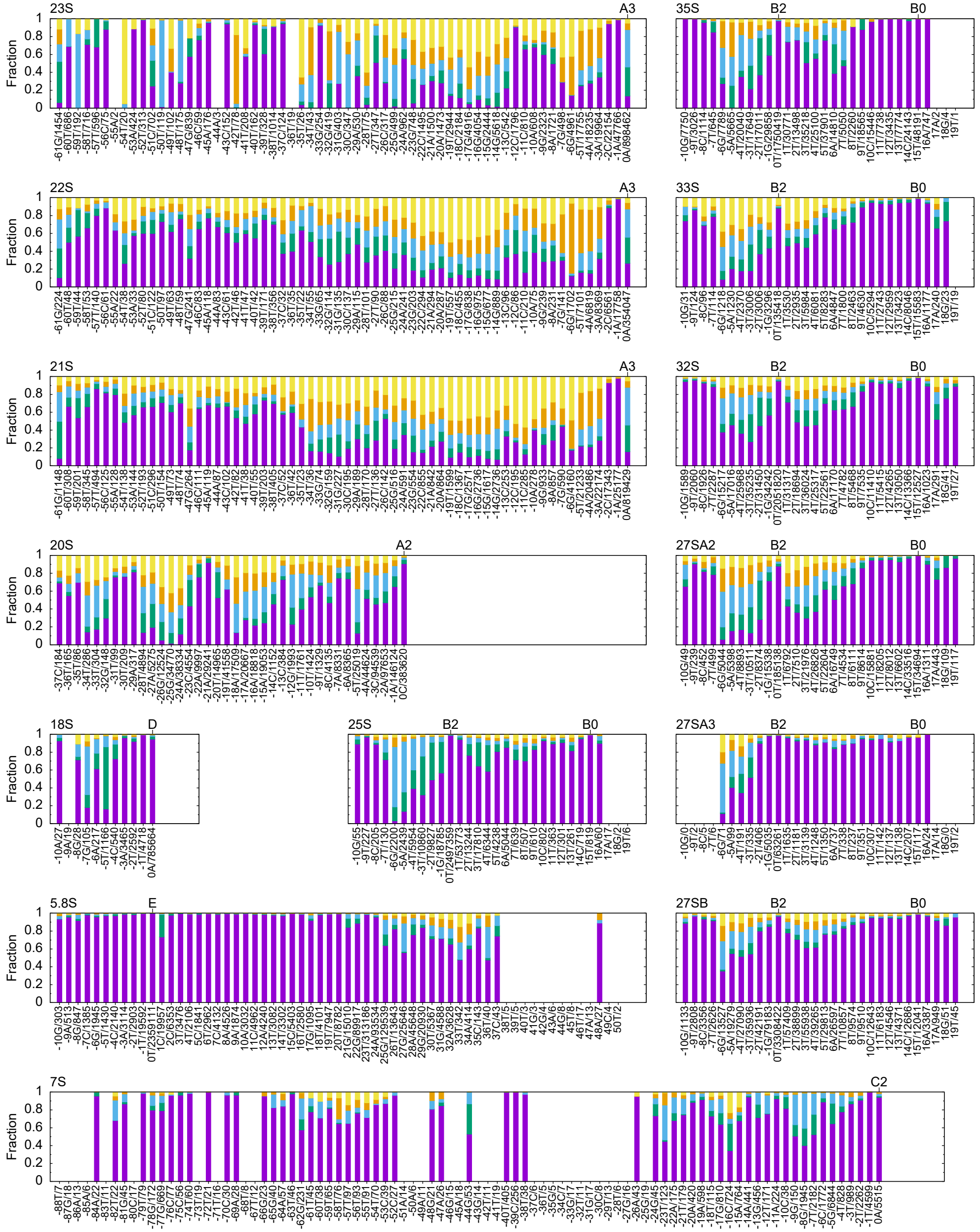

Fig S10. trf4 NOC4

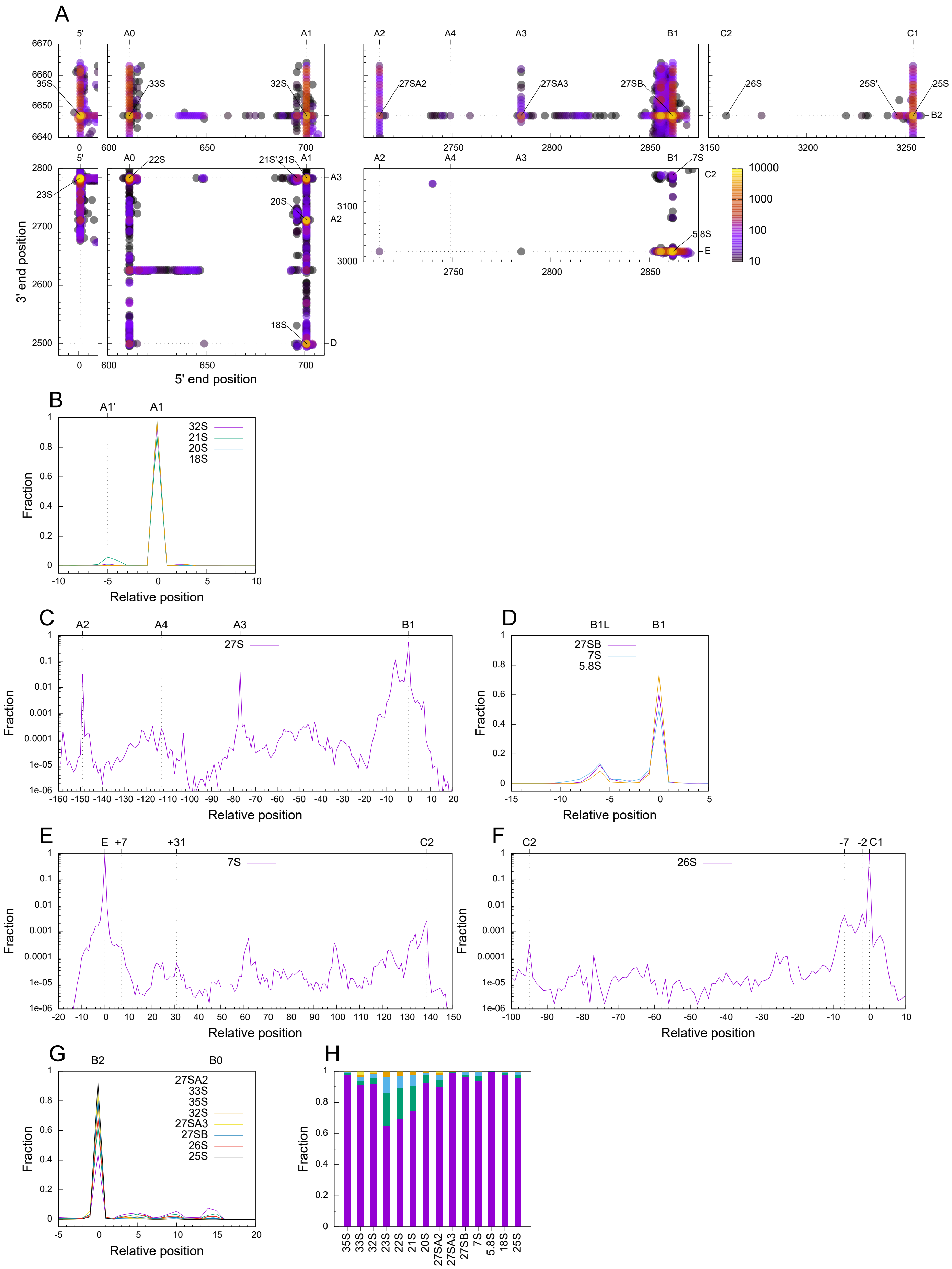

Fig S10. trf4 NOC4

I

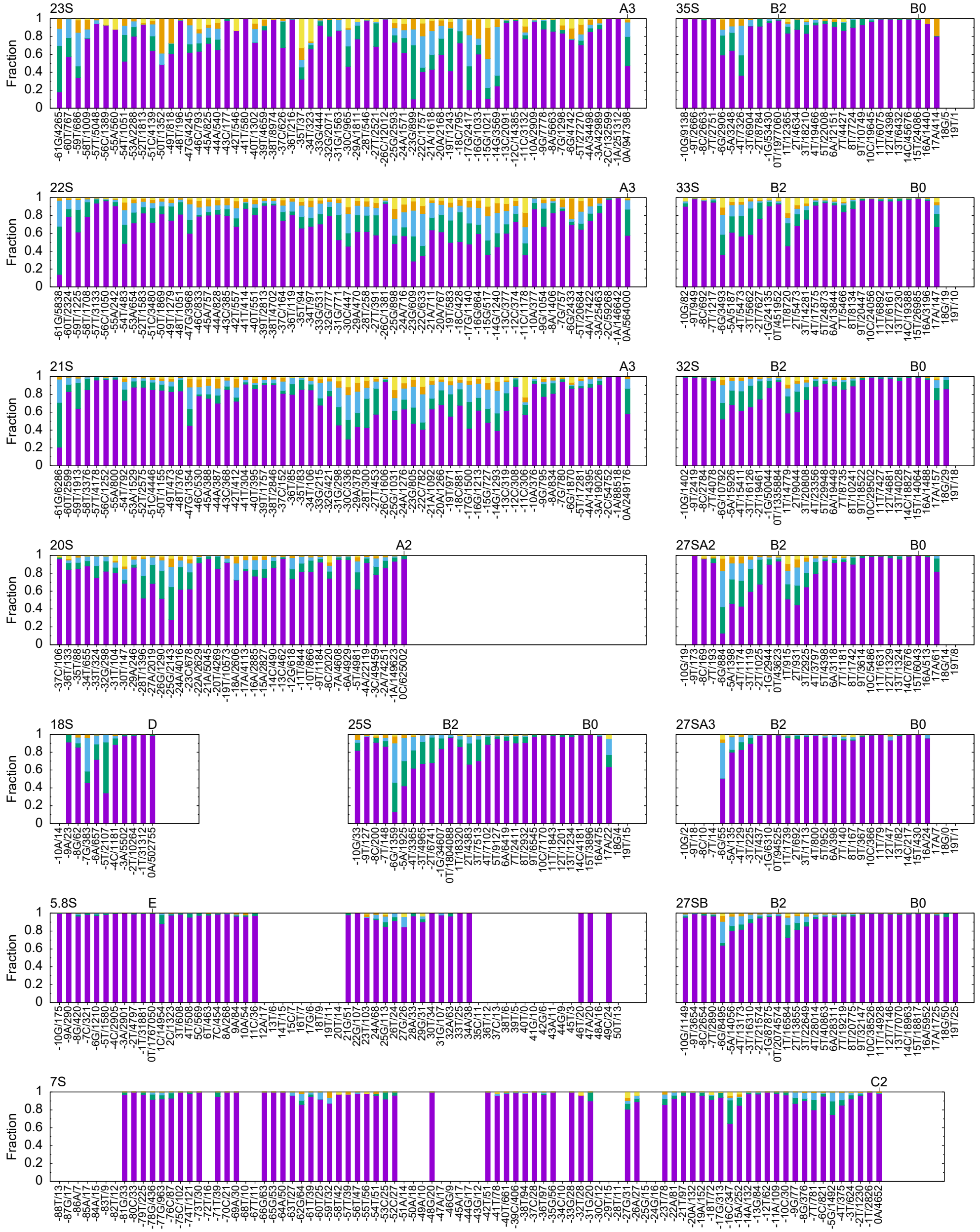

Fig S11. trf5 NOC4

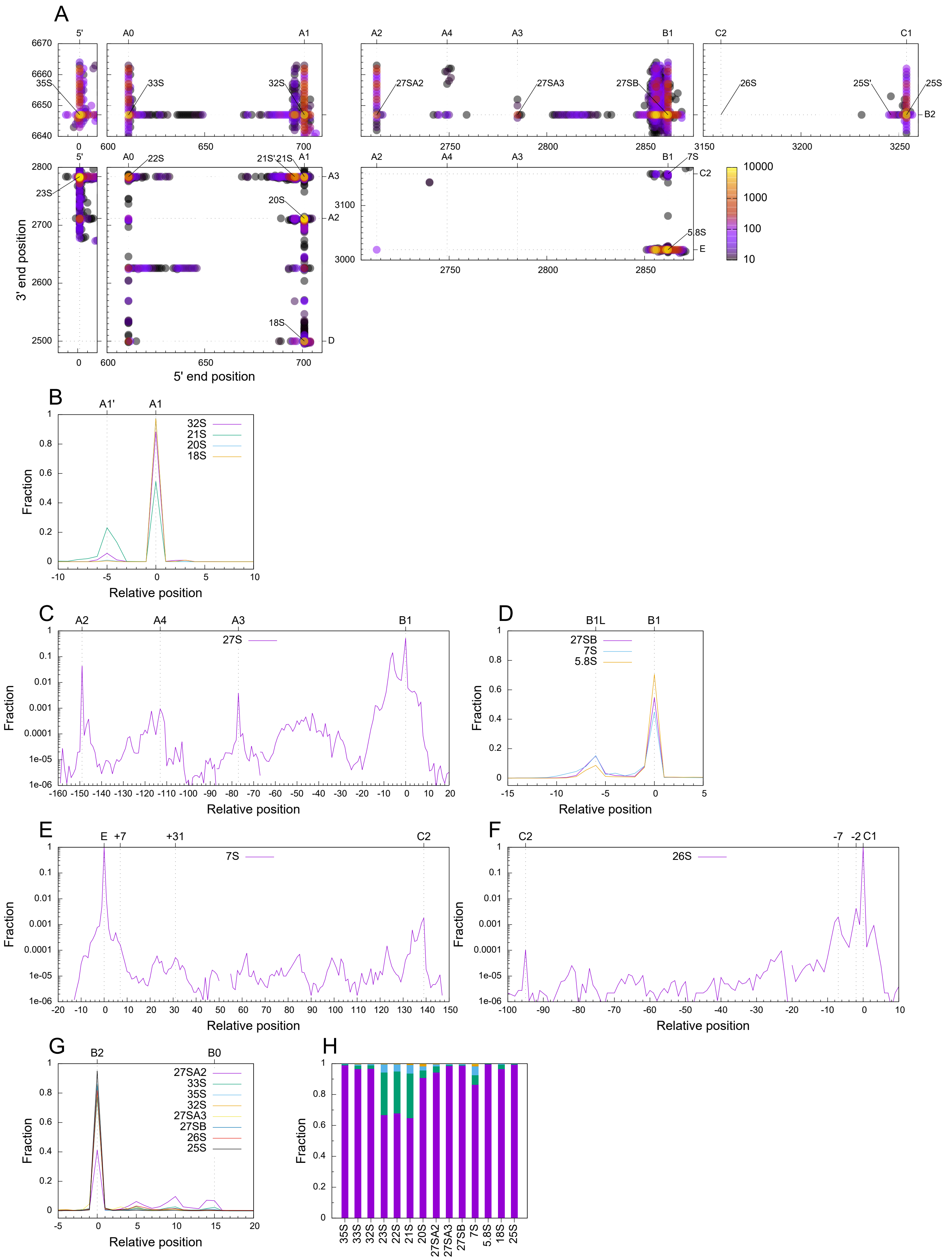

Fig S11. trf5 NOC4

I

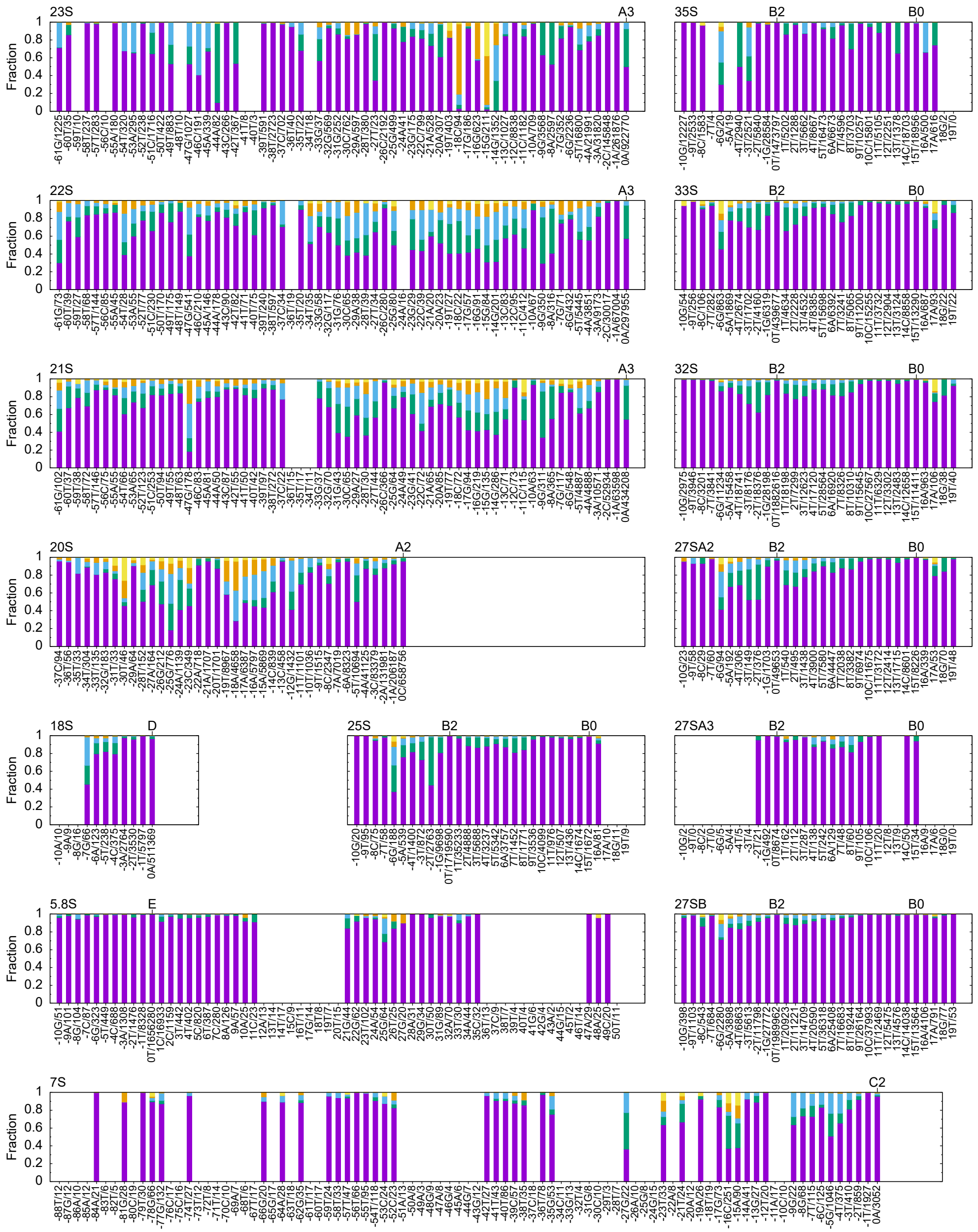

Fig S12. WT NOP7 rep1

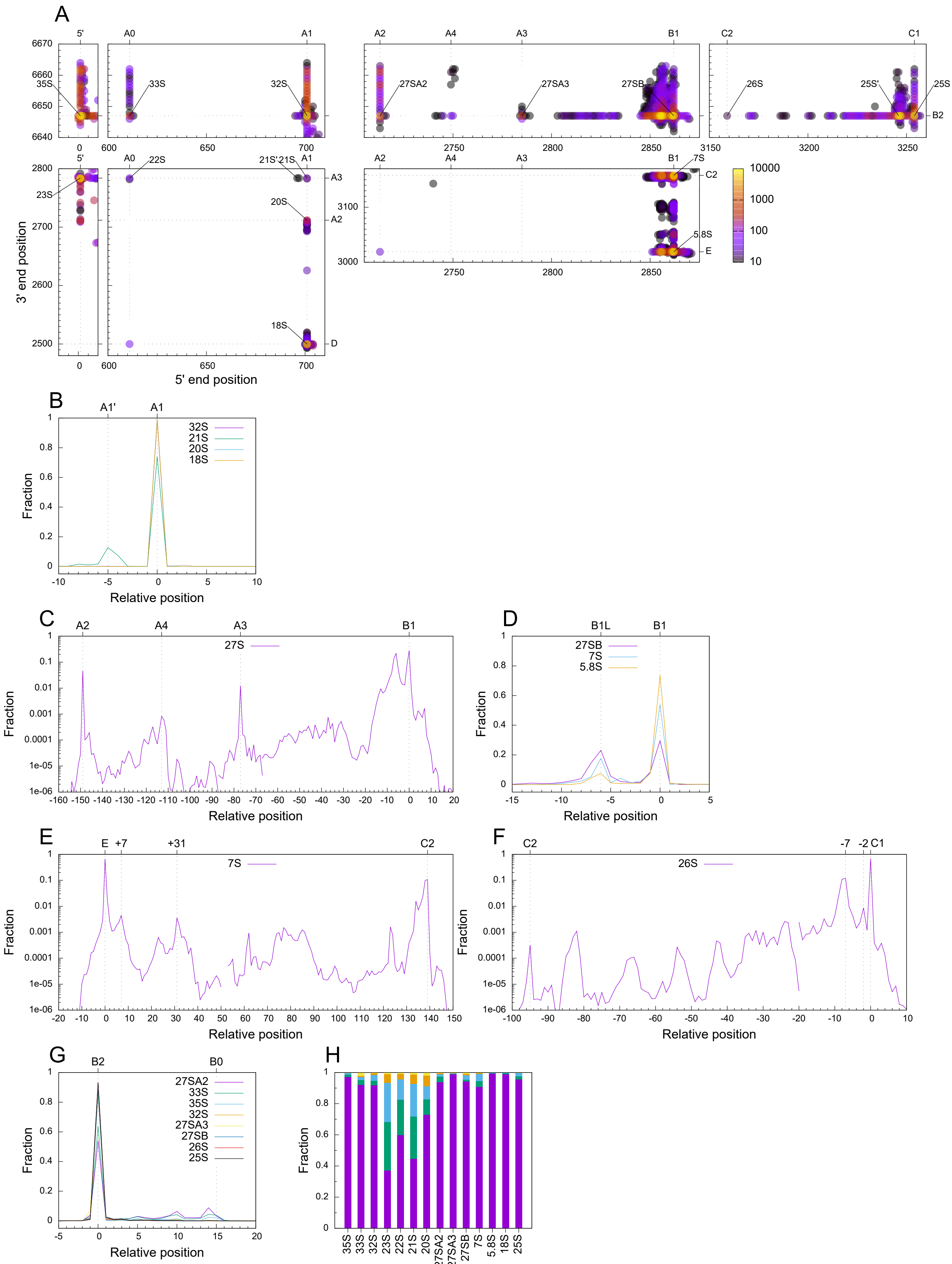

Fig S12. WT NOP7 rep1

I

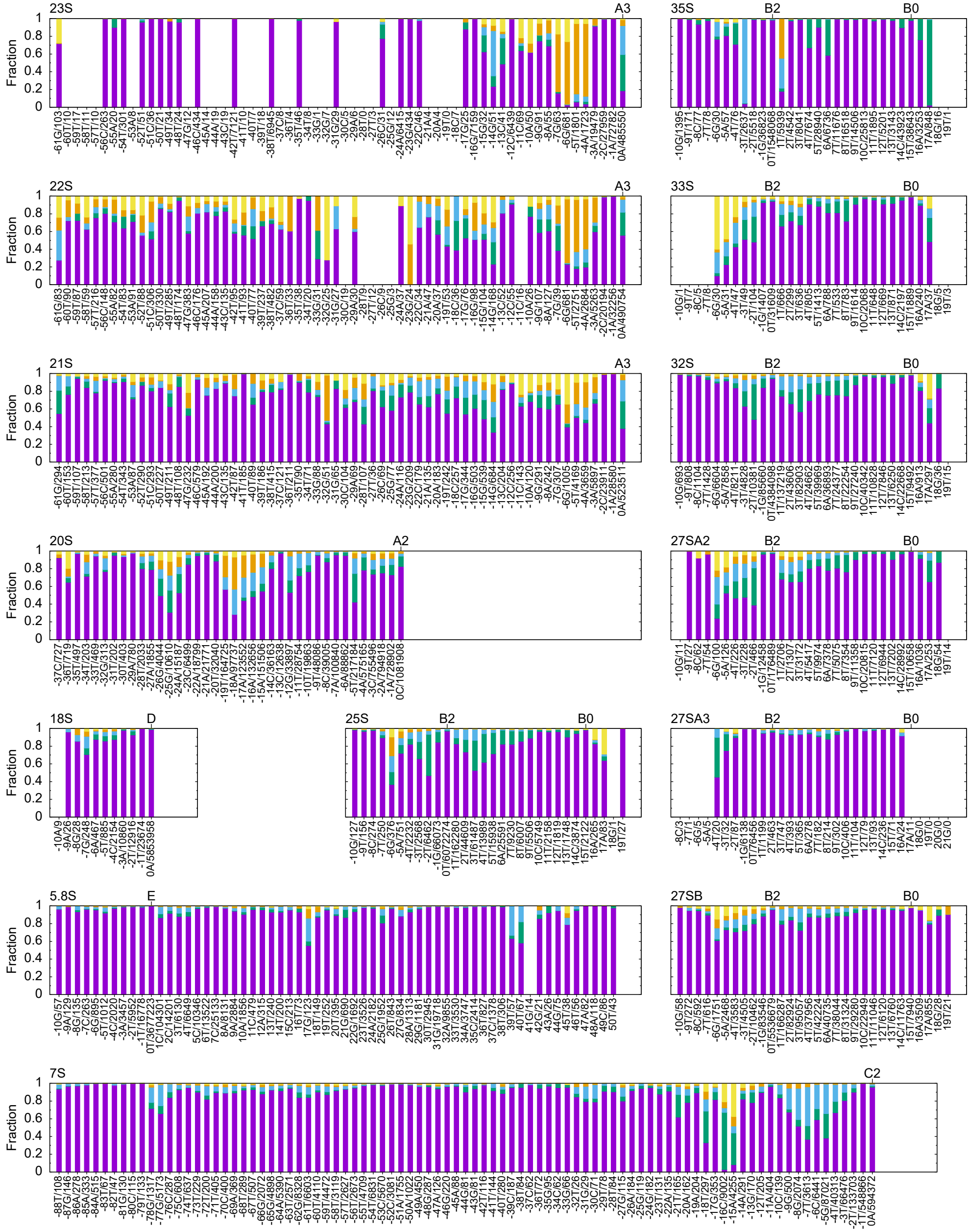

Fig S13. WT NOP7 rep2

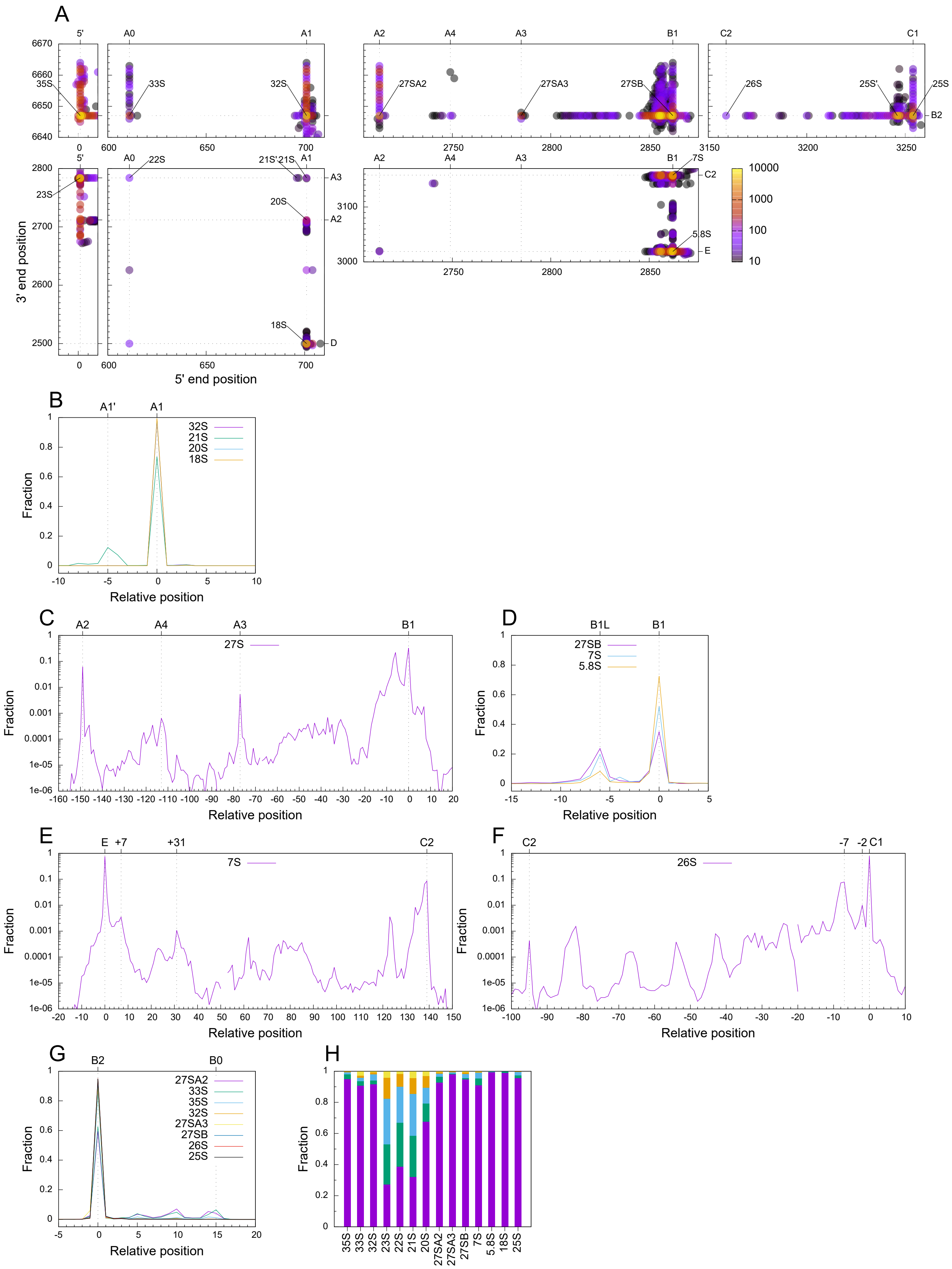

Fig S13. WT NOP7 rep2

I

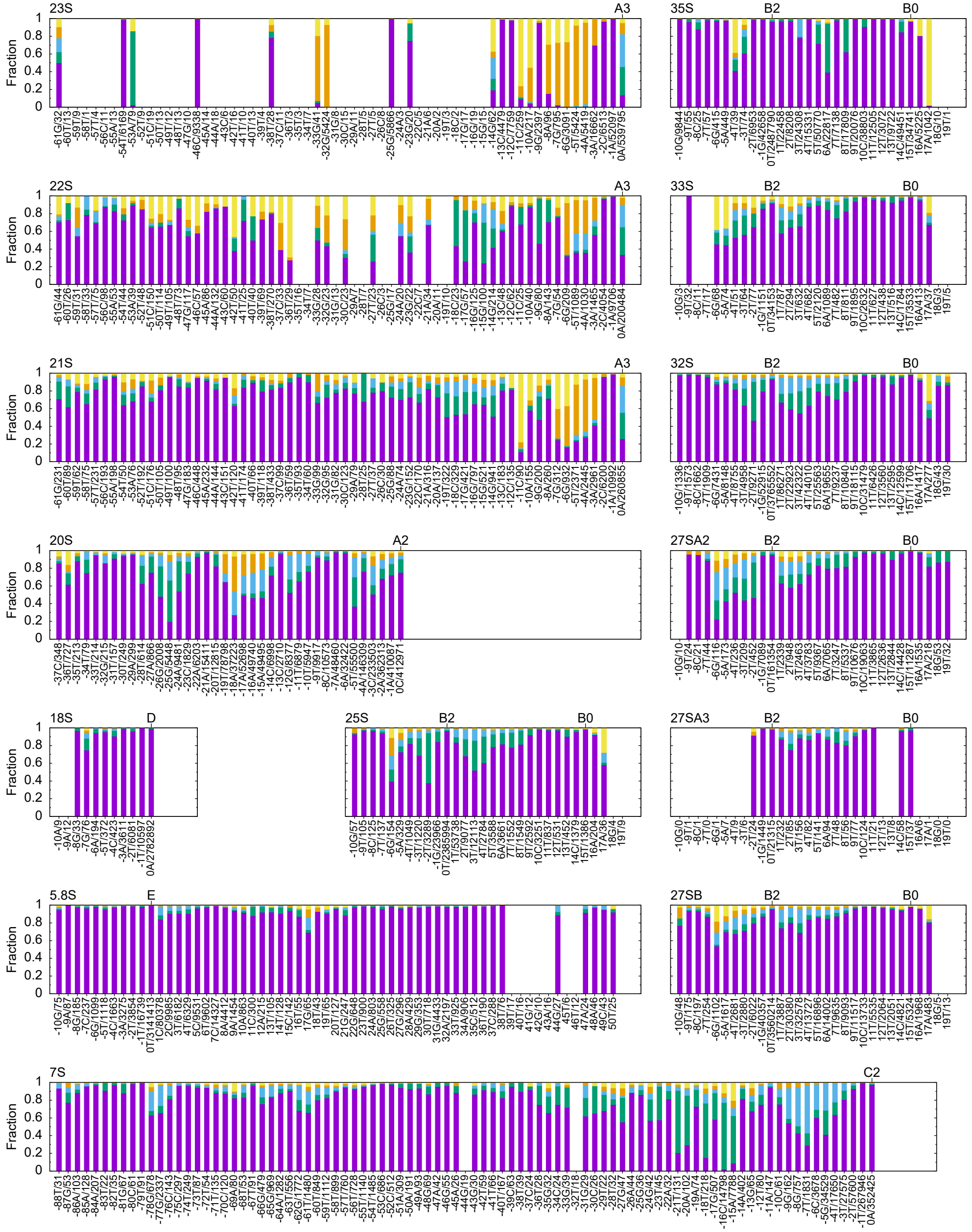

Fig S14. rex1 NOP7 rep1

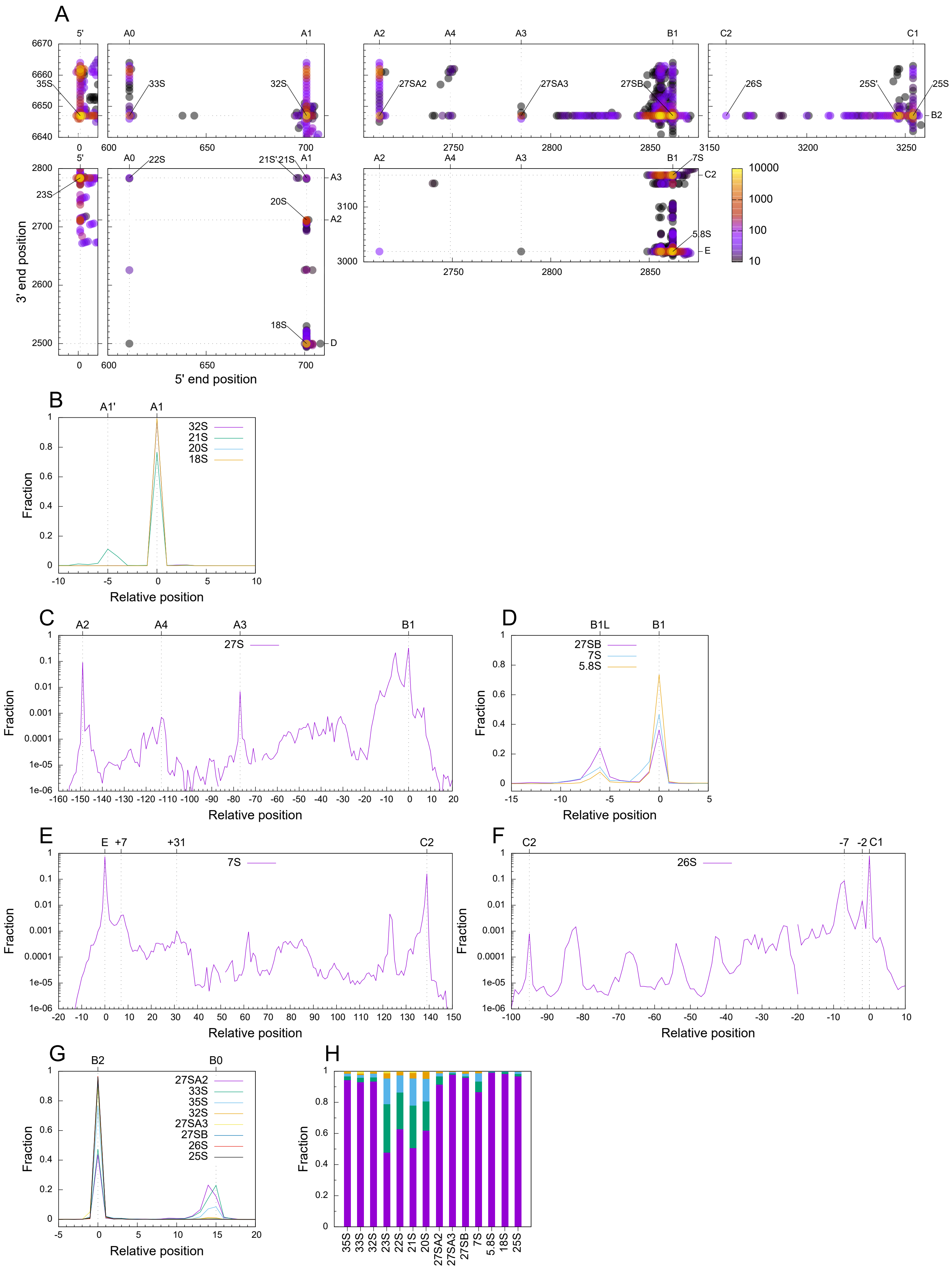

Fig S14. rex1 NOP7 rep1

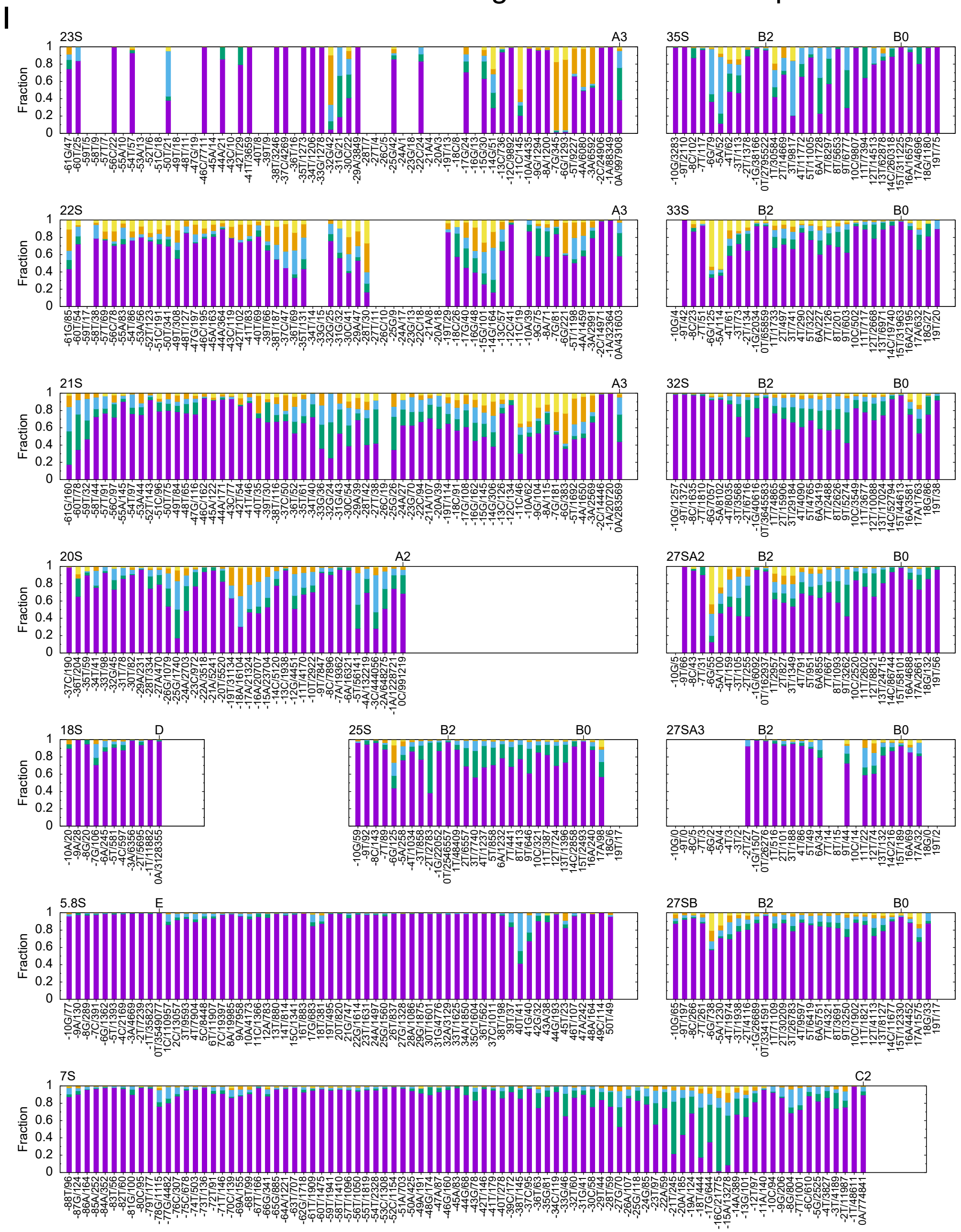

Fig S15. rex1 NOP7 rep2

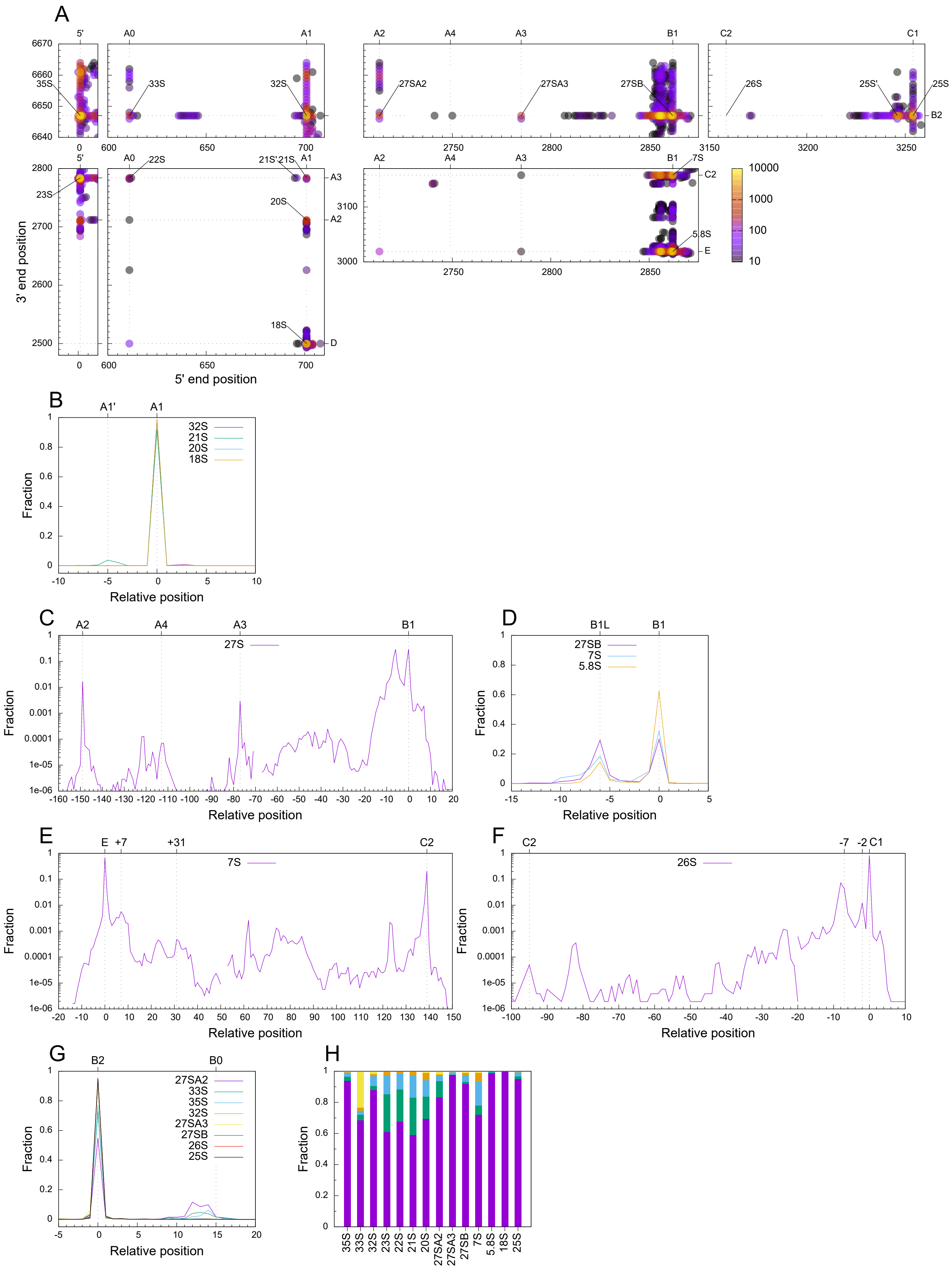

Fig S15. rex1 NOP7 rep2

I

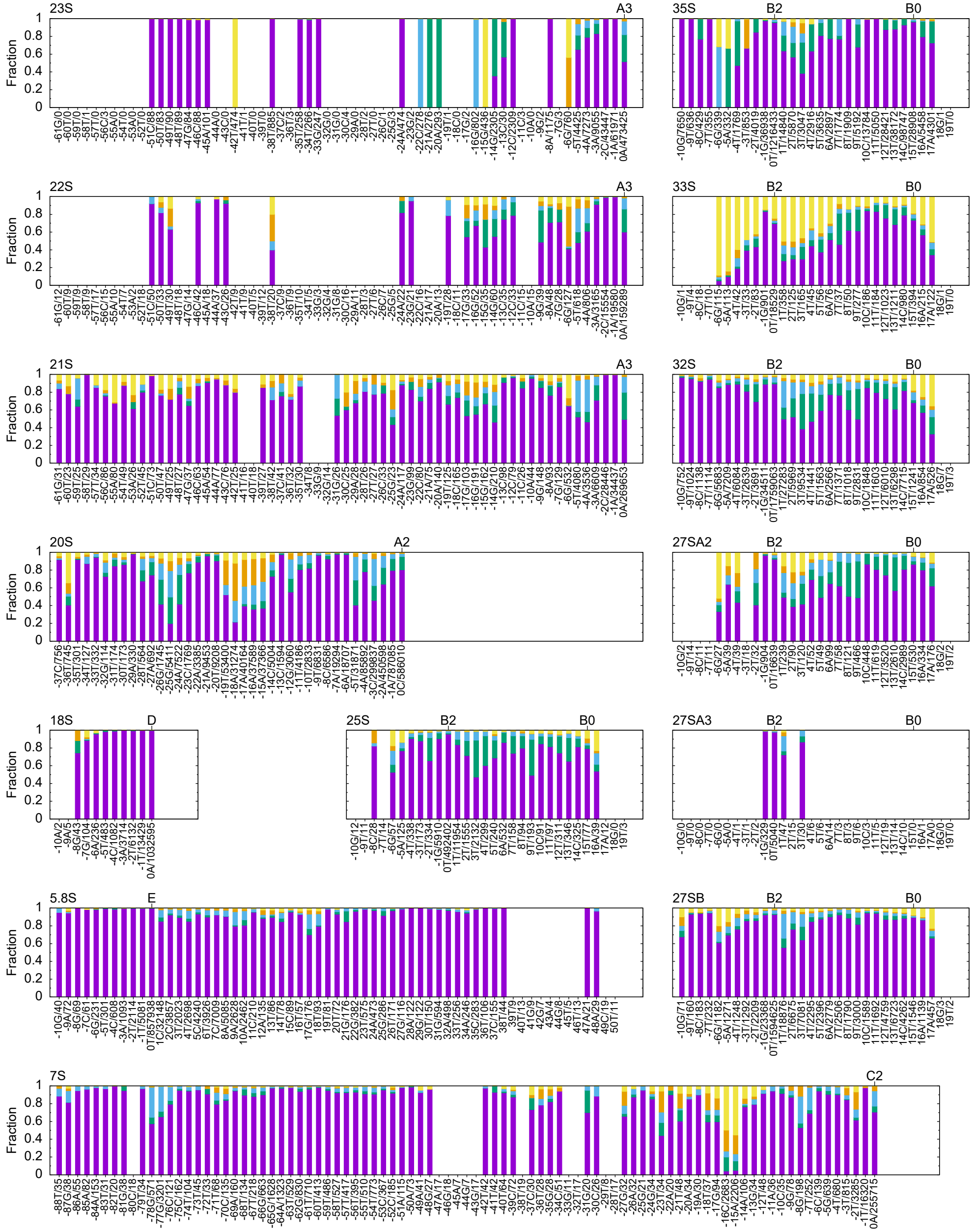

Fig S16. rex2 NOP7 rep1

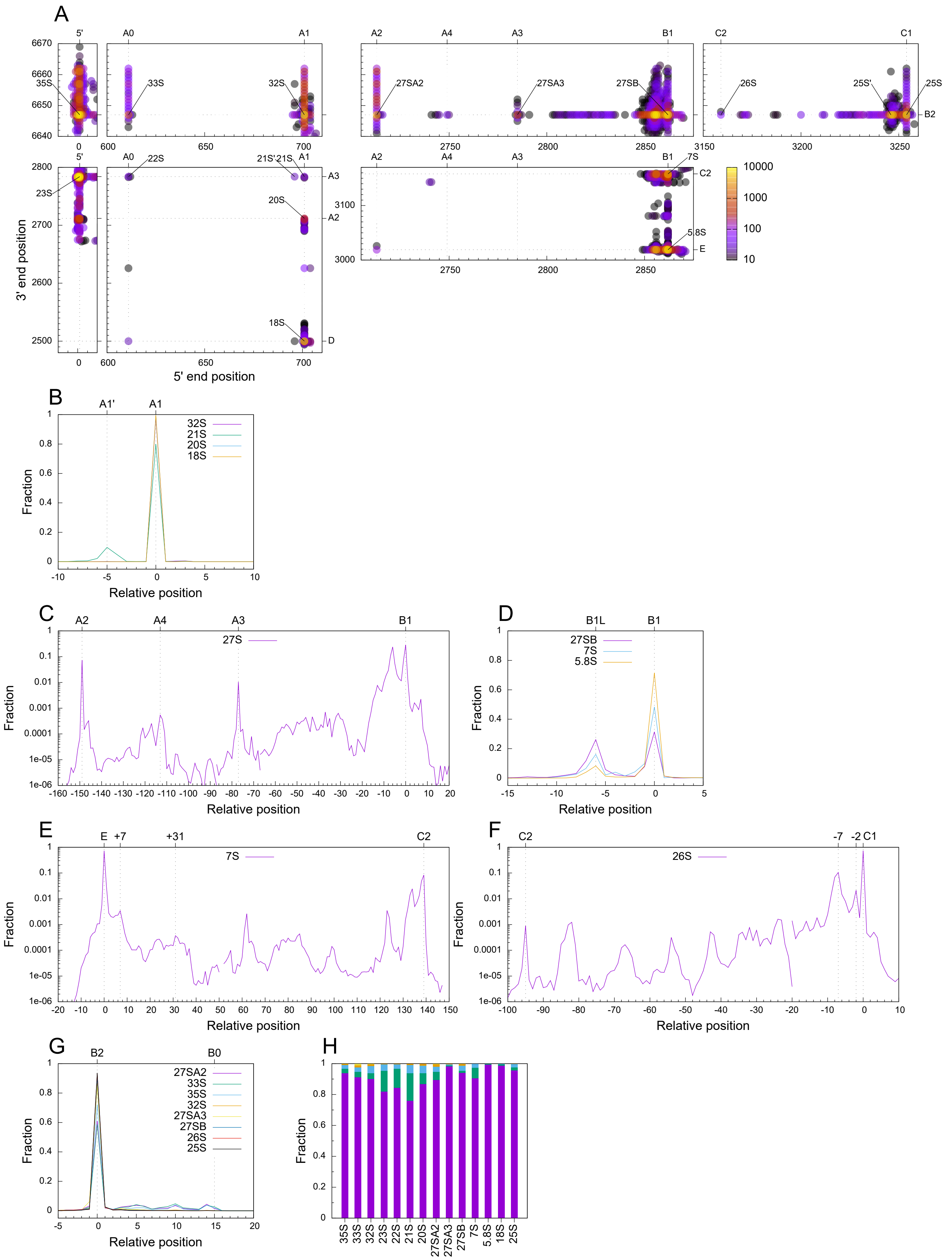

Fig S16. rex2 NOP7 rep1

I

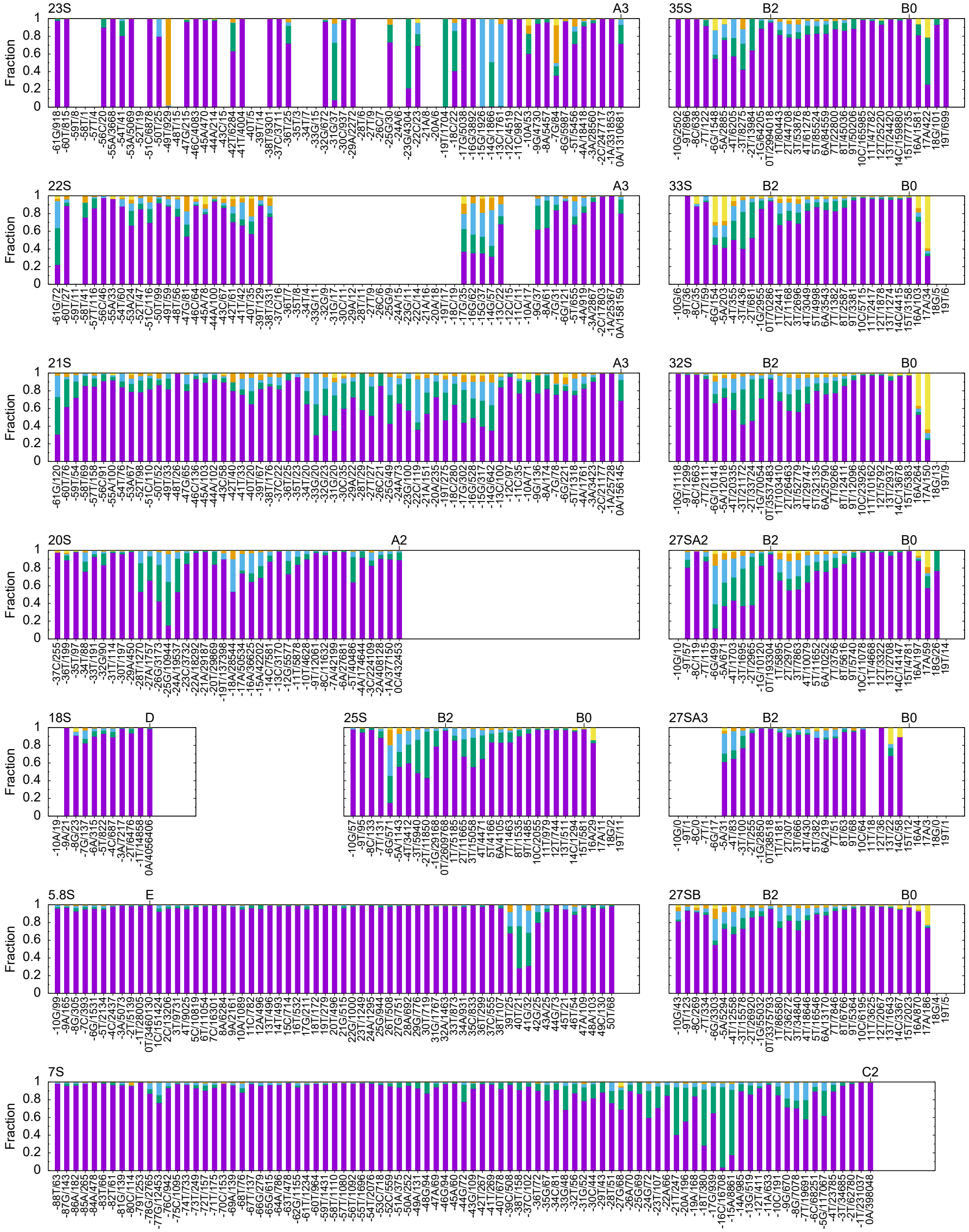

Fig S17. rex2 NOP7 rep2

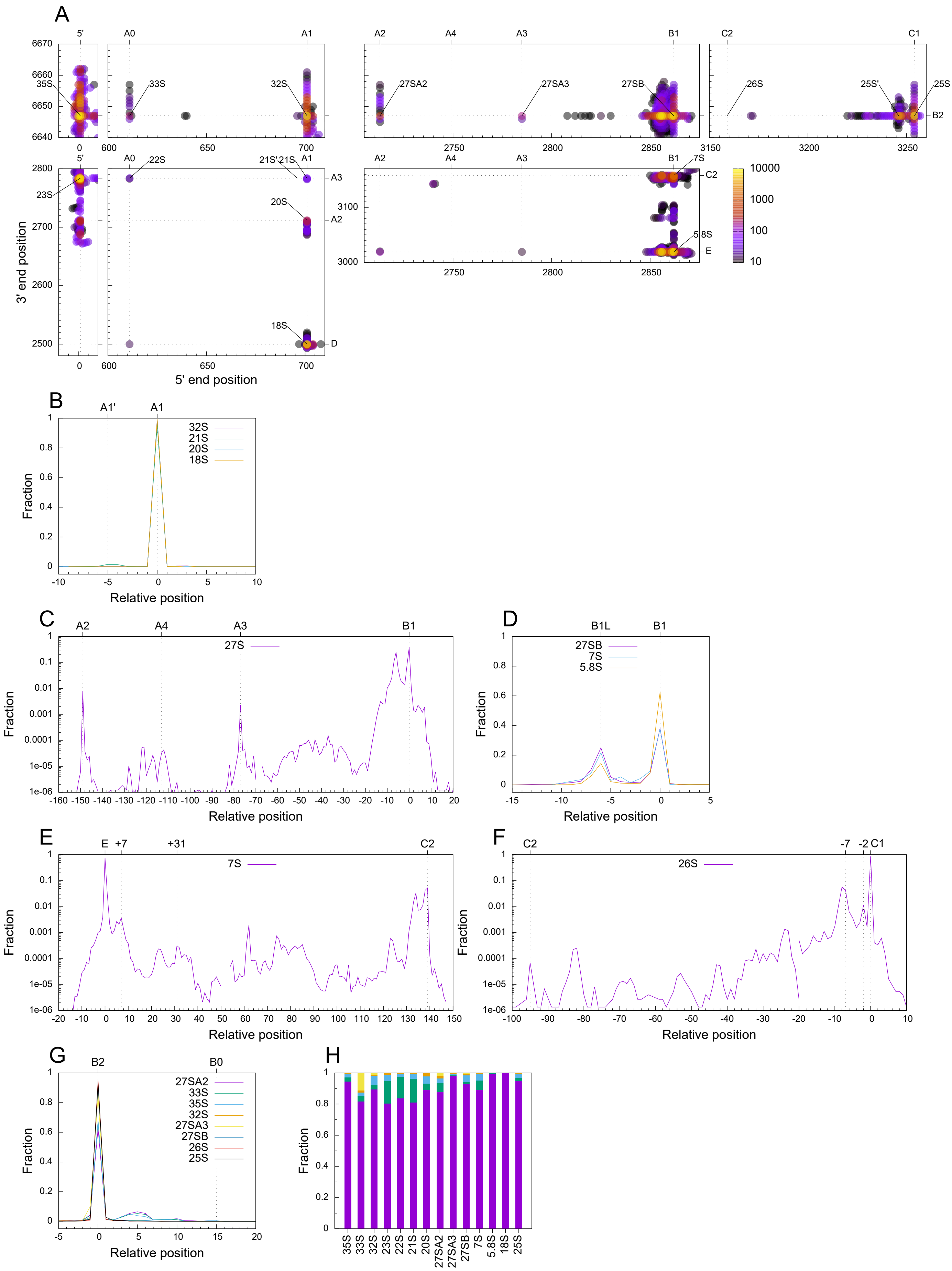

Fig S17. rex2 NOP7 rep2

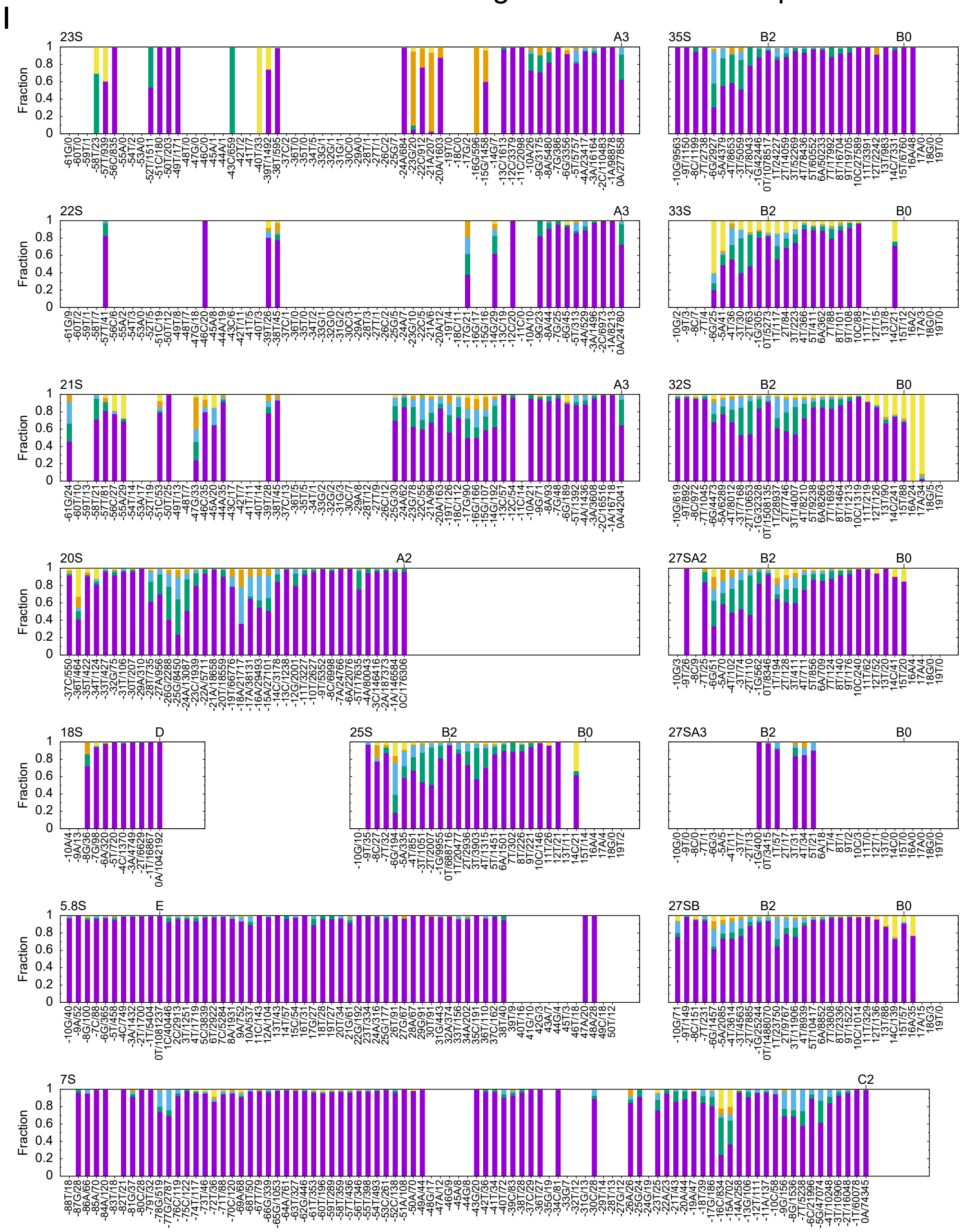

Fig S18. rex3 NOP7 rep1

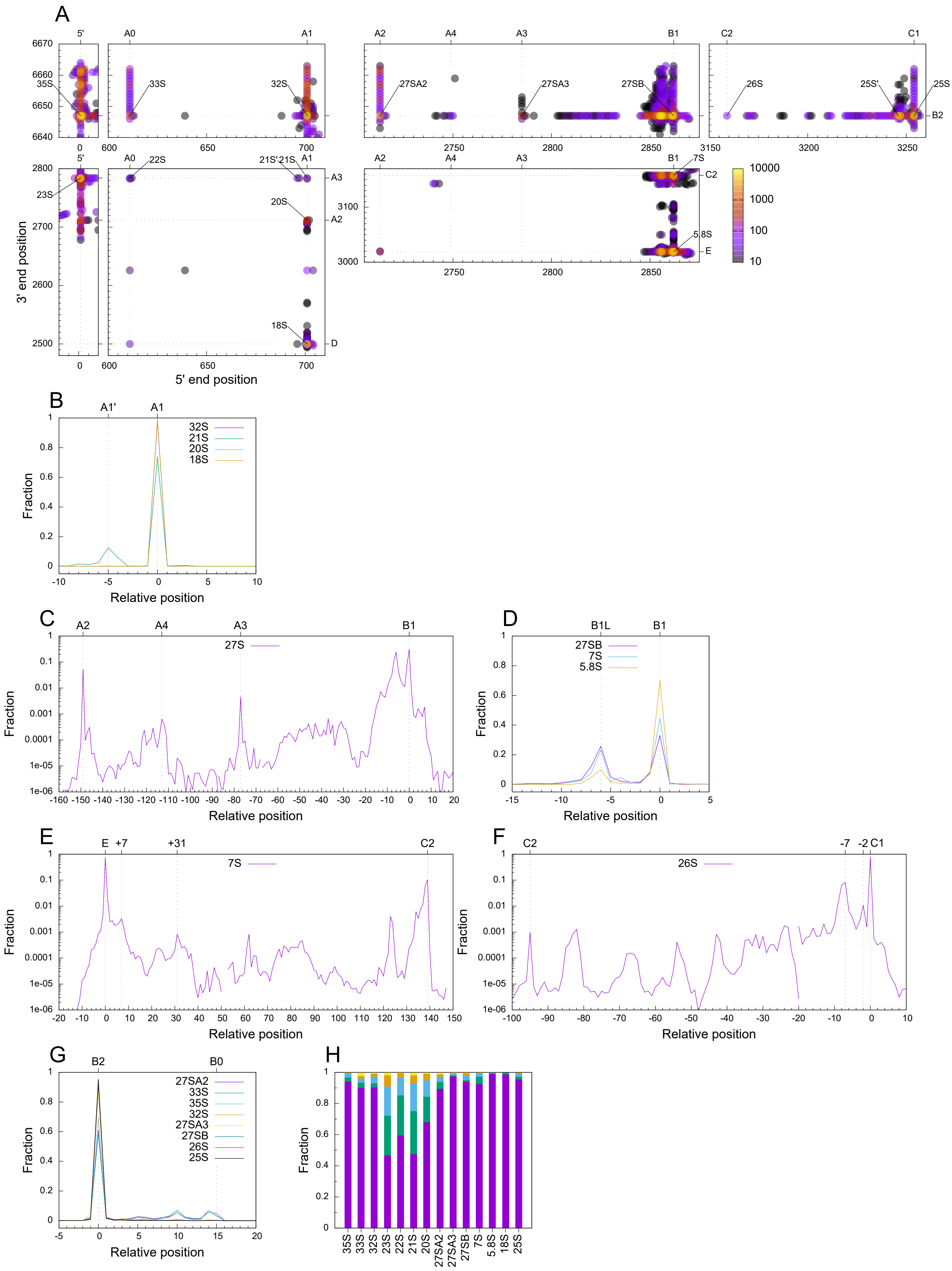

Fig S18. rex3 NOP7 rep1

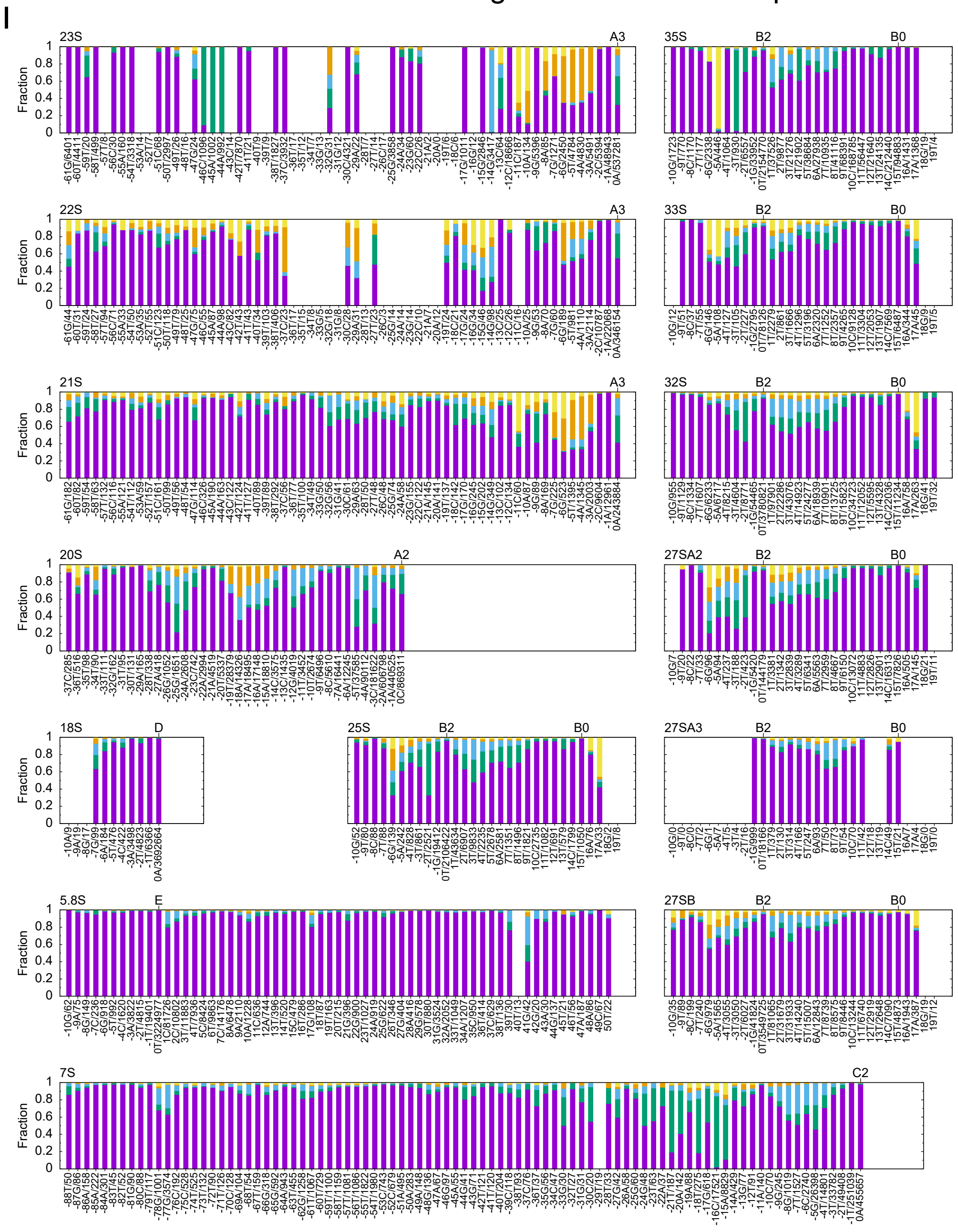

Fig S19. rex3 NOP7 rep2

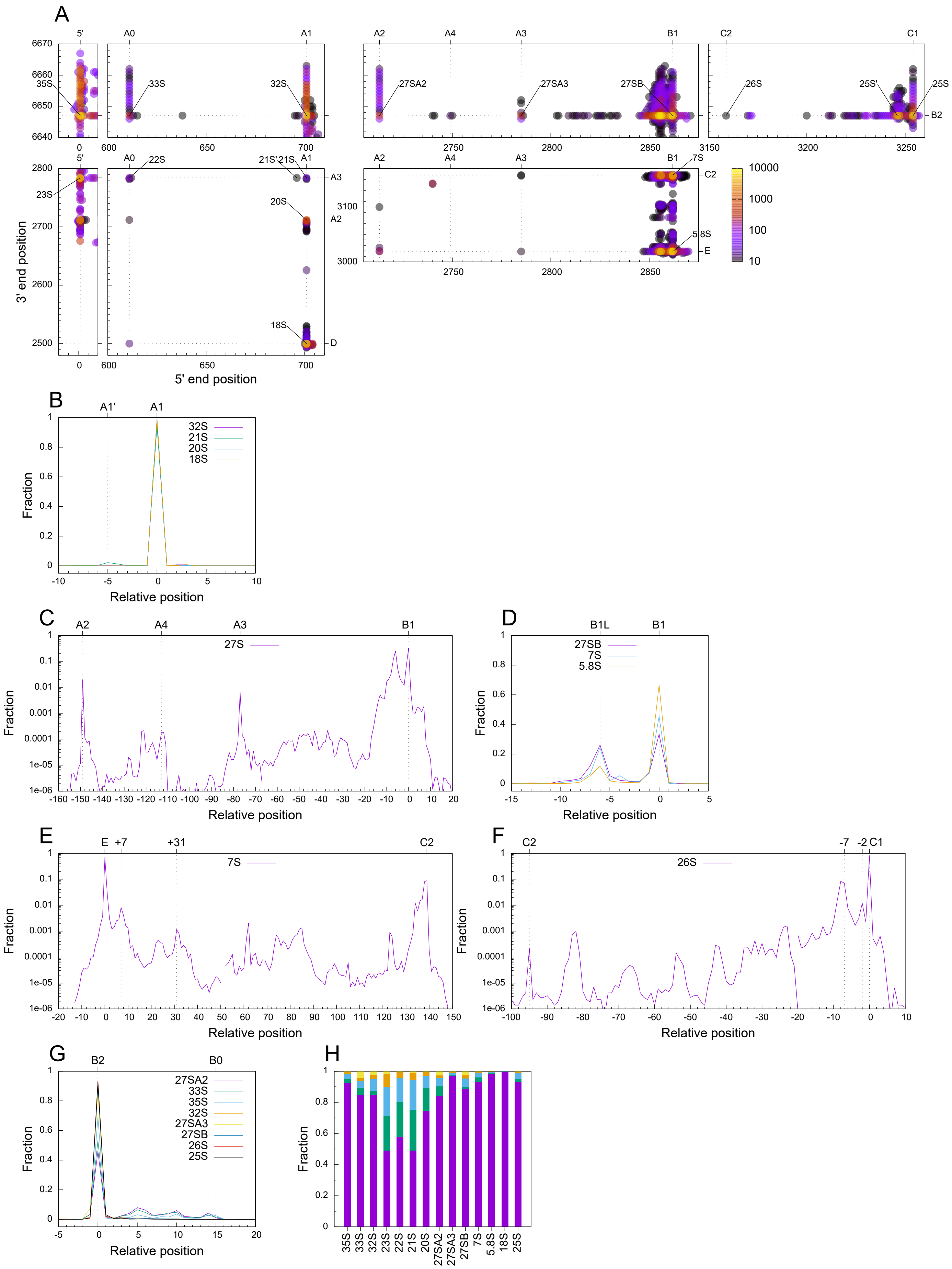

Fig S19. rex3 NOP7 rep2

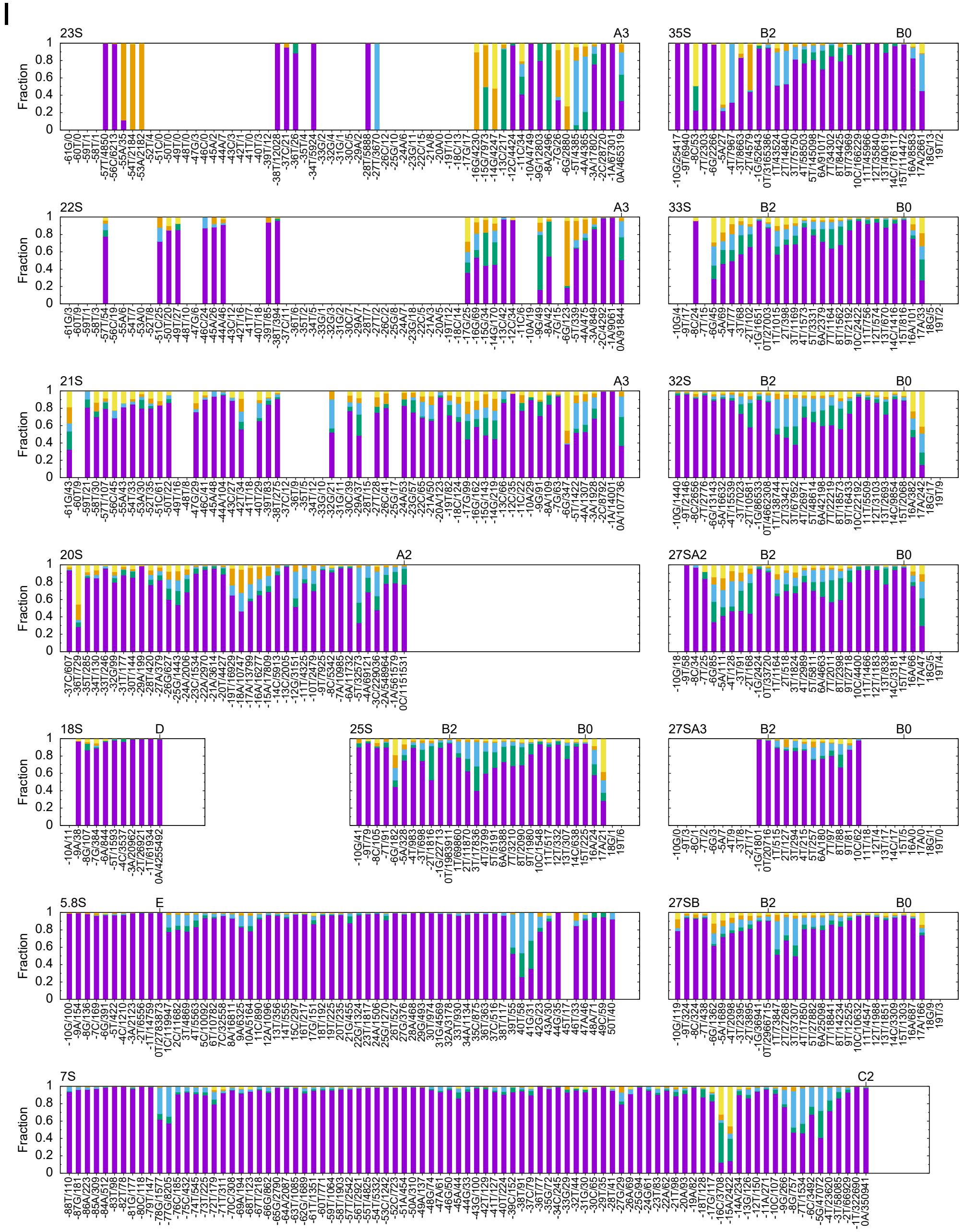

Fig S20. rex4 NOP7 rep1

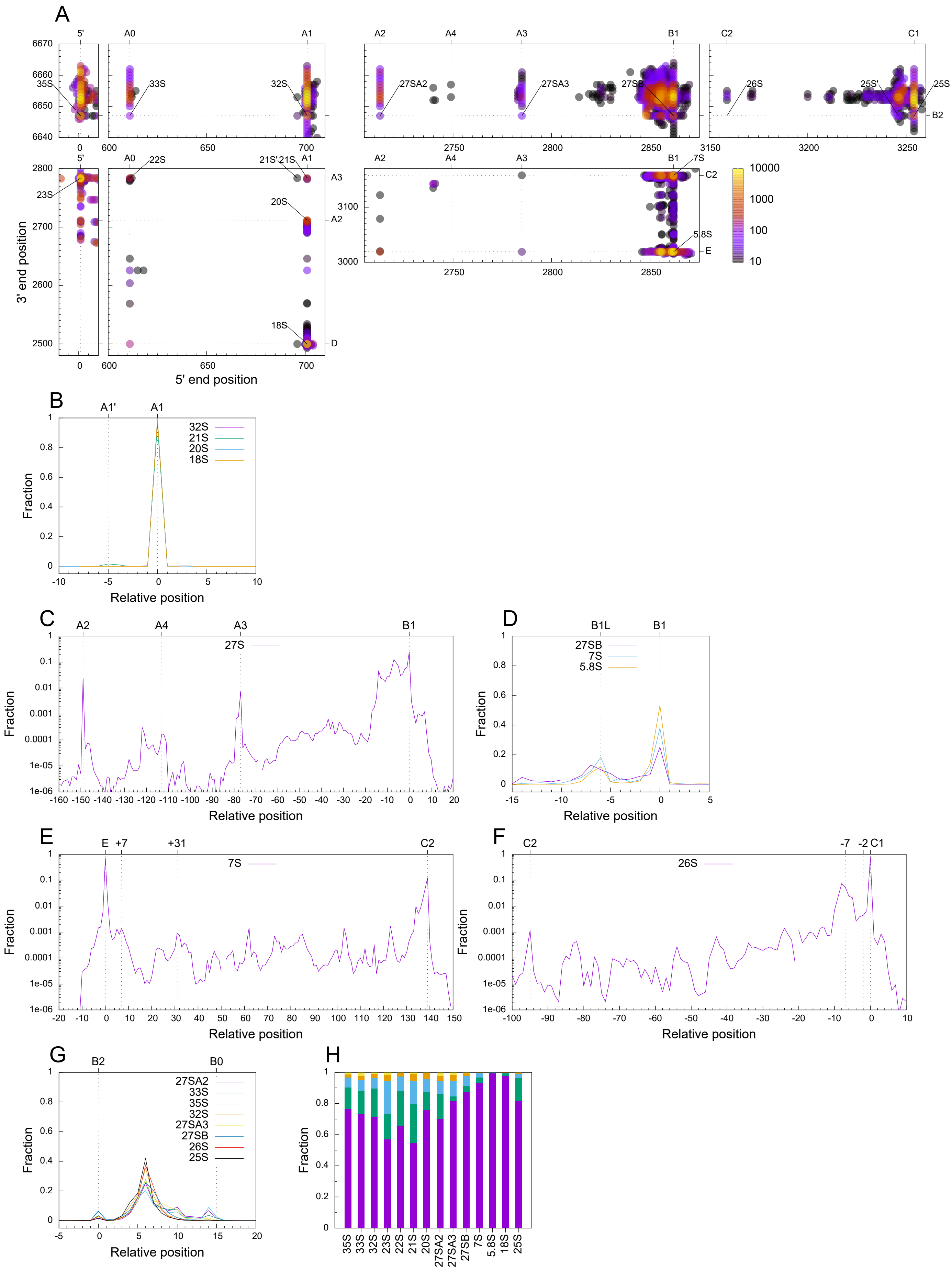

Fig S20. rex4 NOP7 rep1

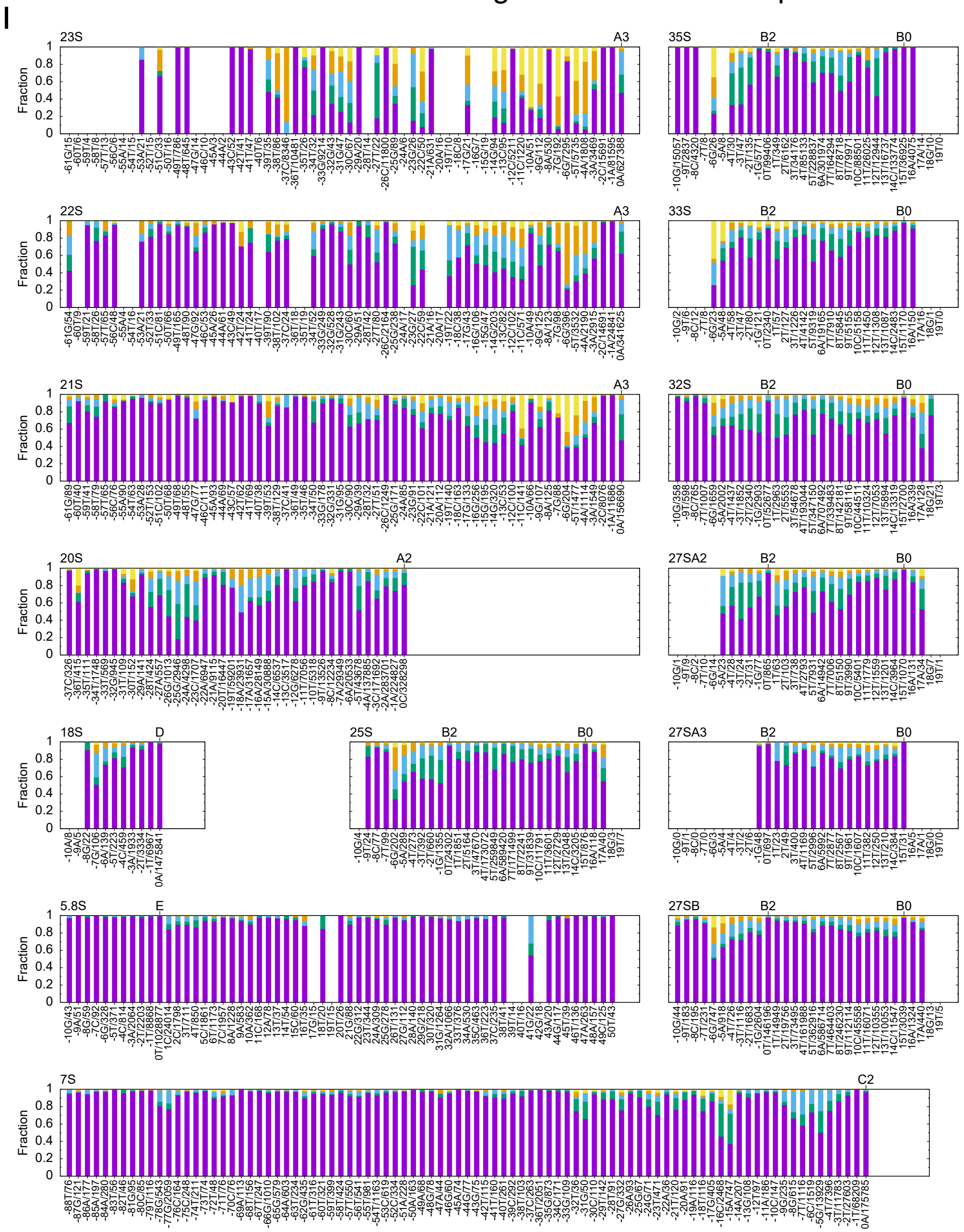

Fig S21. rex4 NOP7 rep2

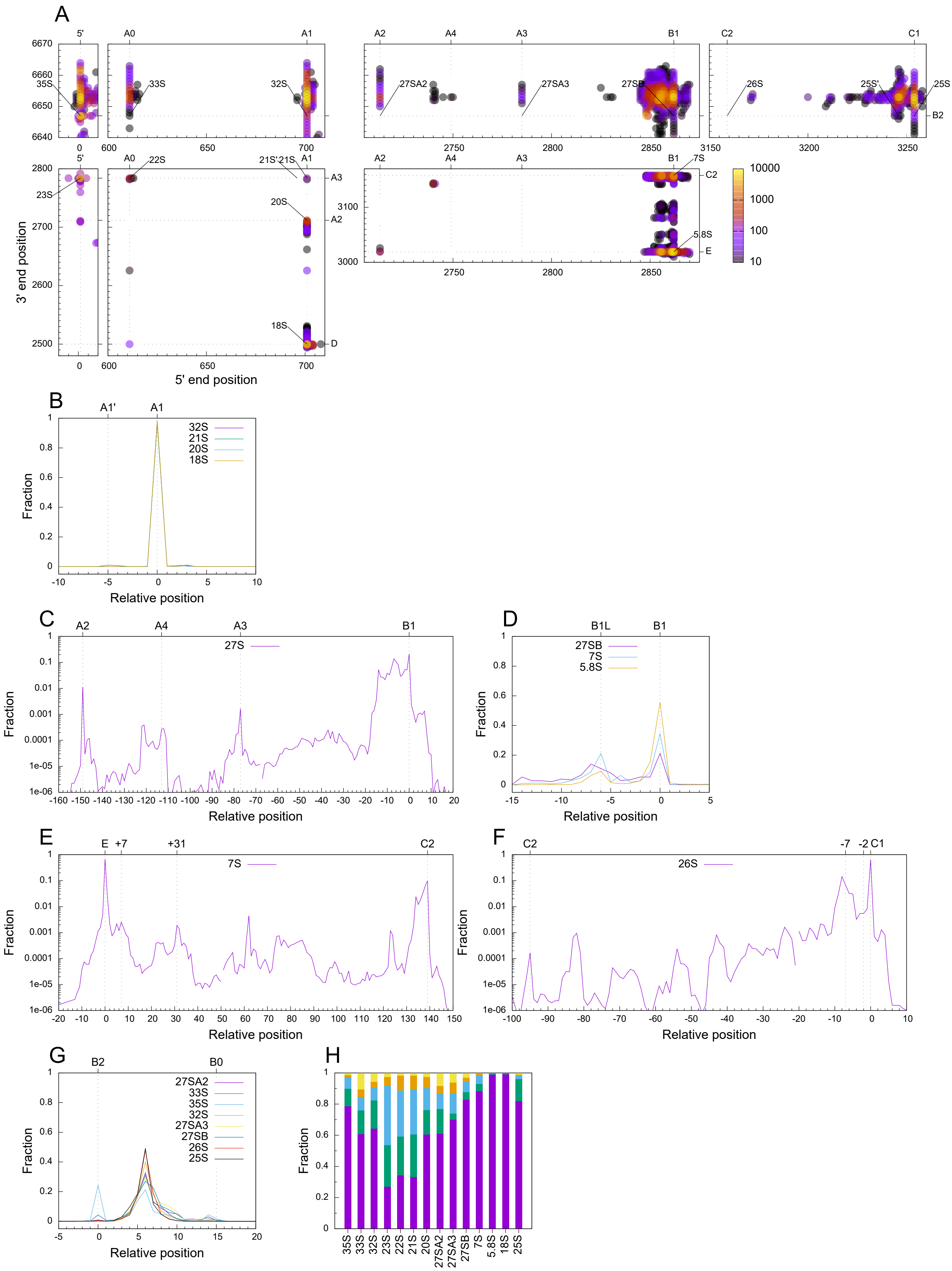

Fig S21. rex4 NOP7 rep2

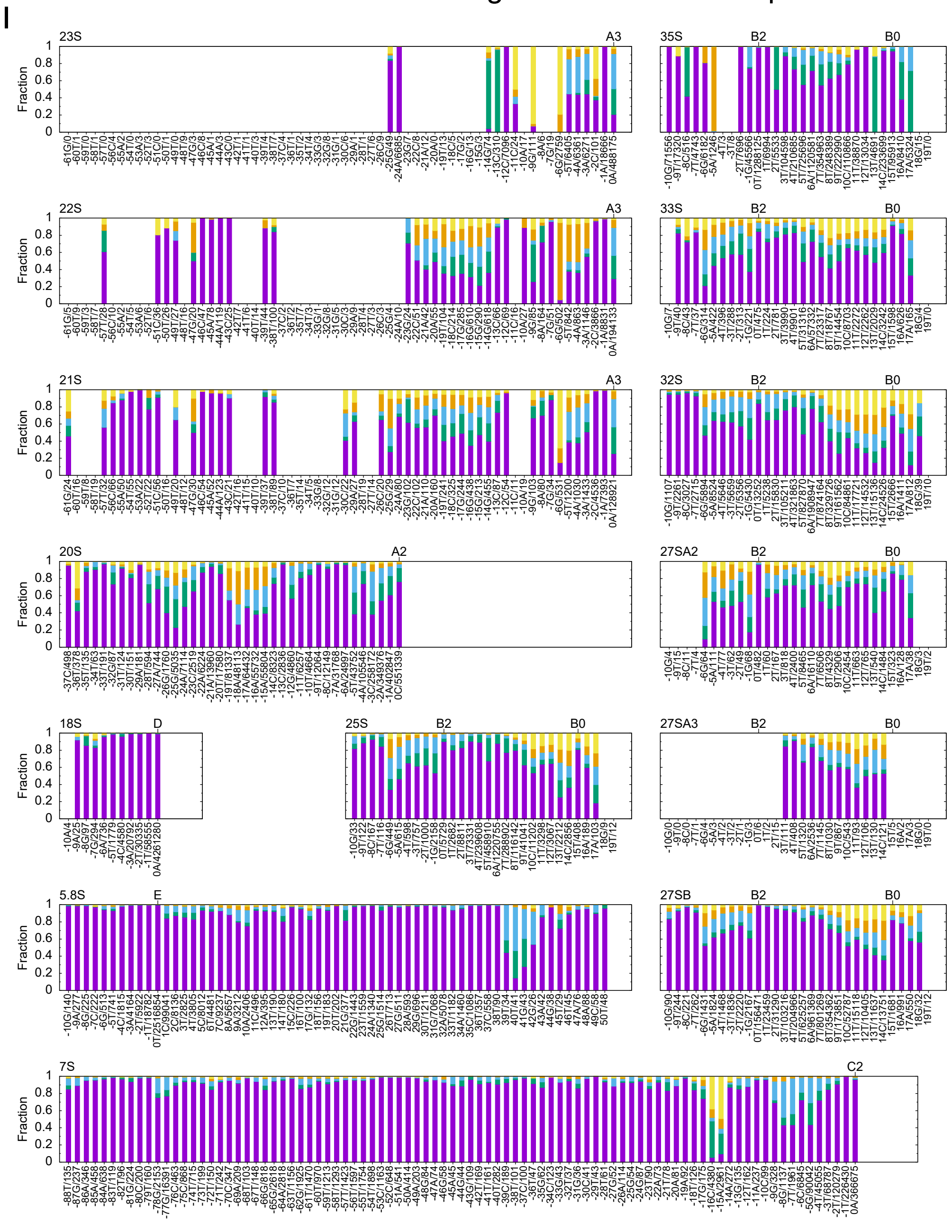

Fig S22. rnt1 NOP7

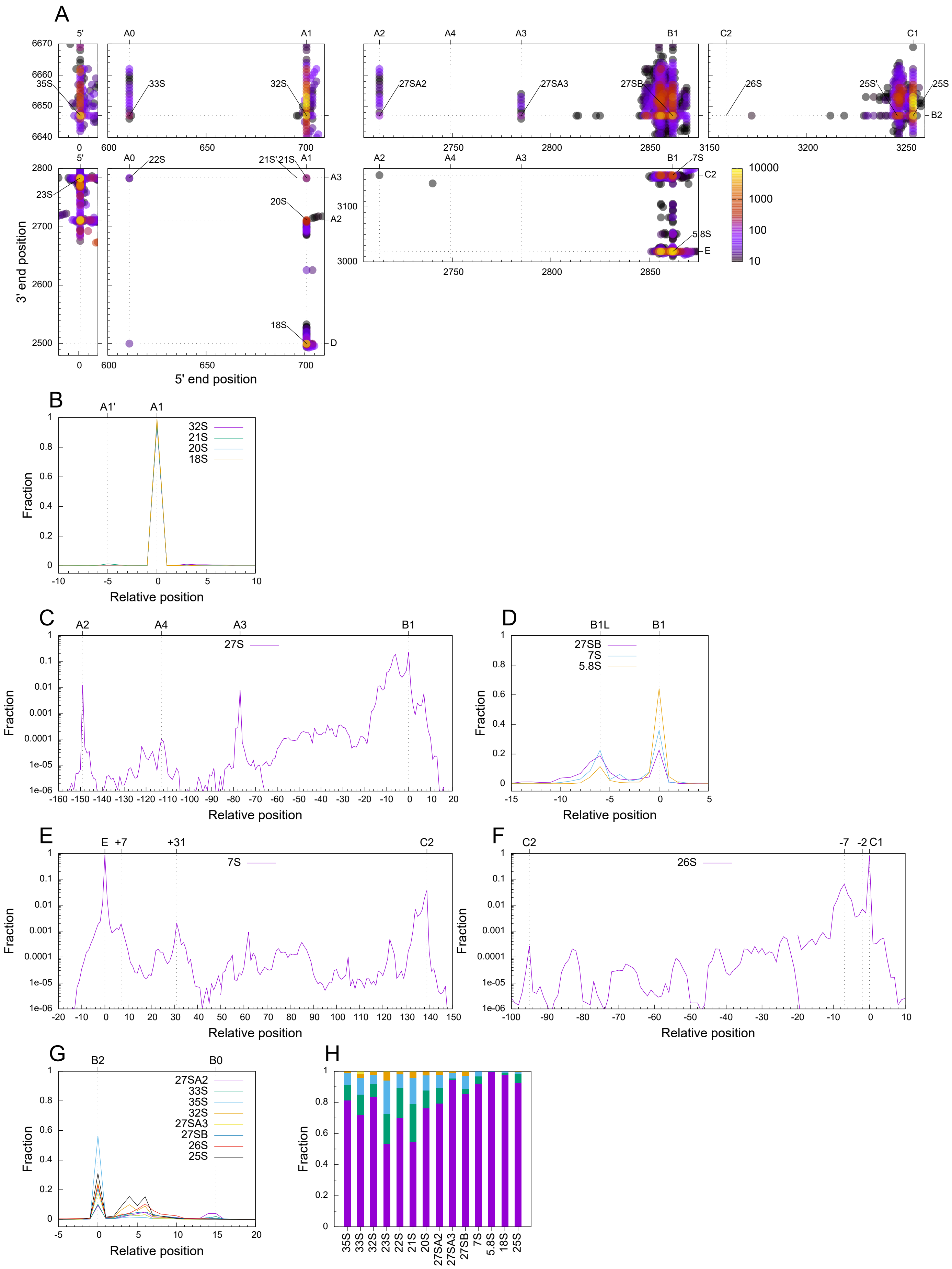

Fig S22. rnt1 NOP7

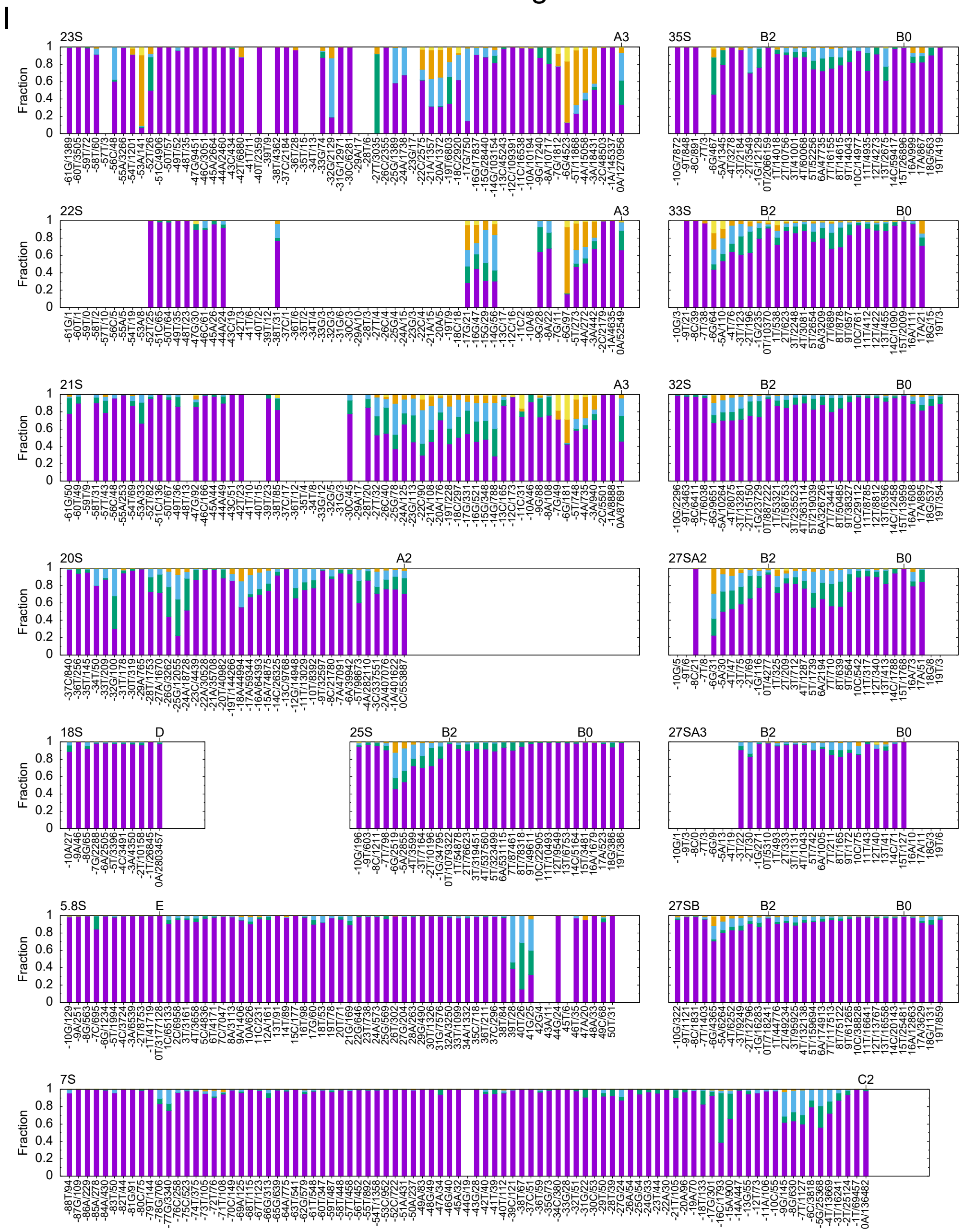

Fig S23. ngl2 NOP7

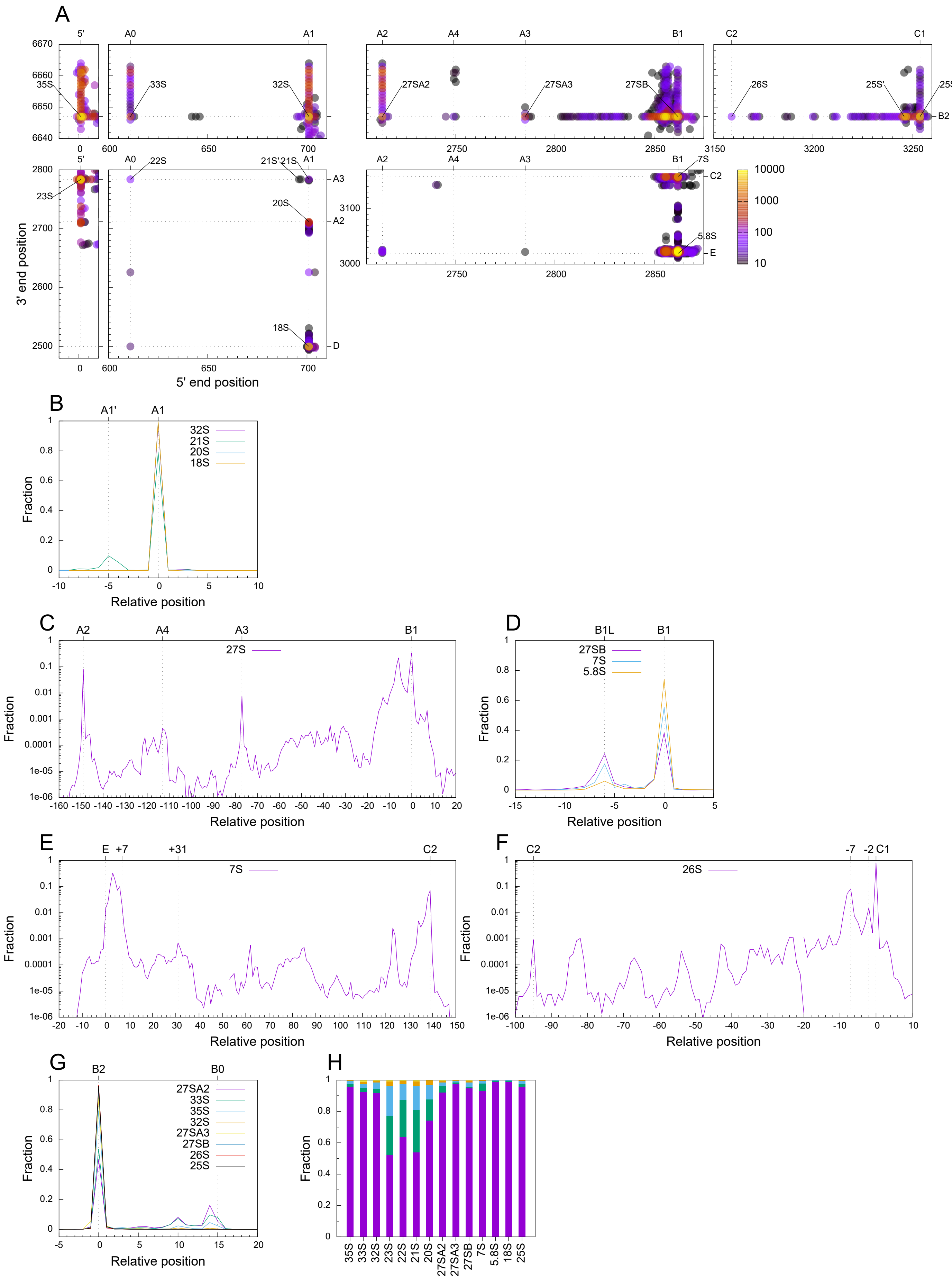

Fig S23. ngl2 NOP7

I

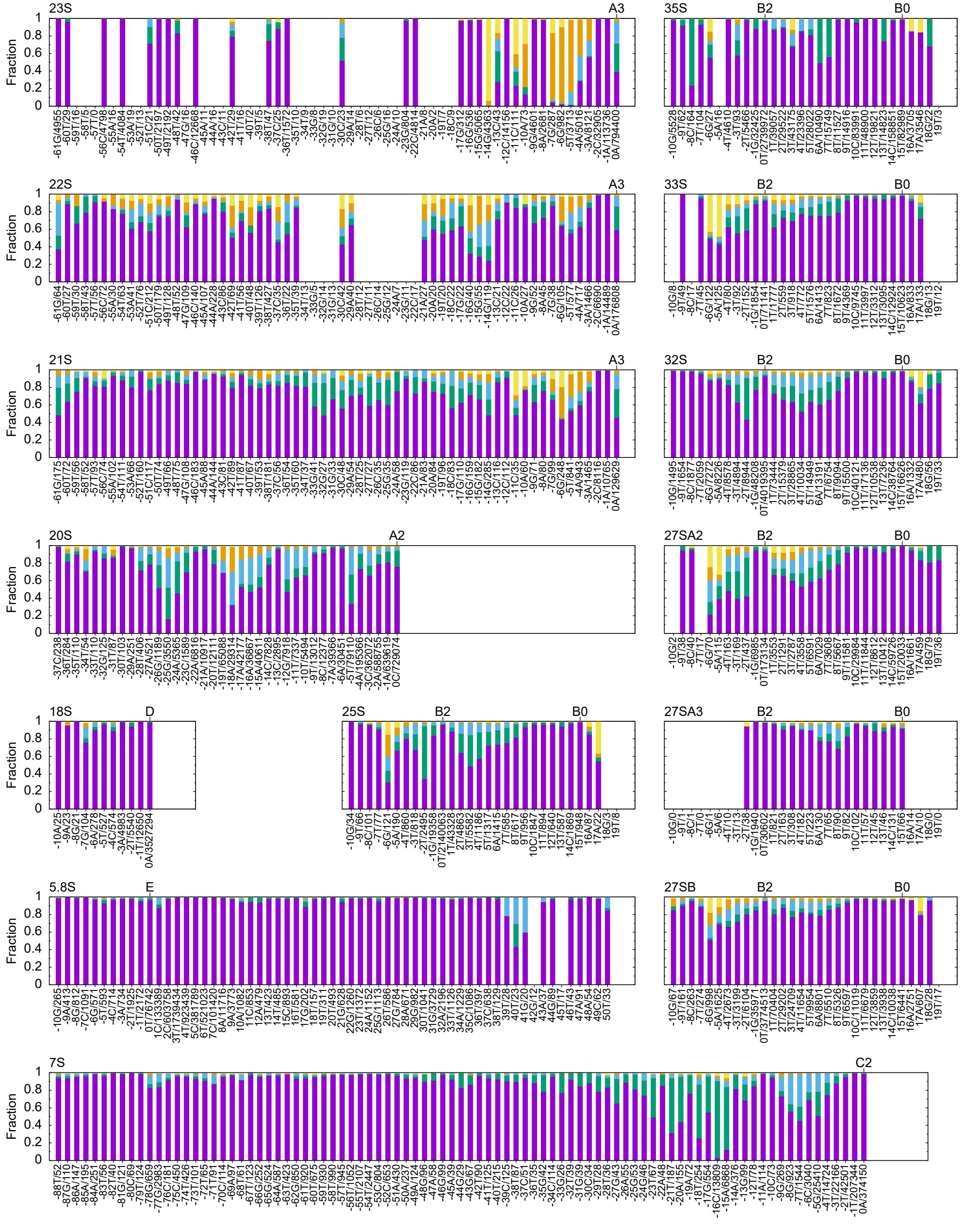

Fig S24. rat1 NOP7 rep1

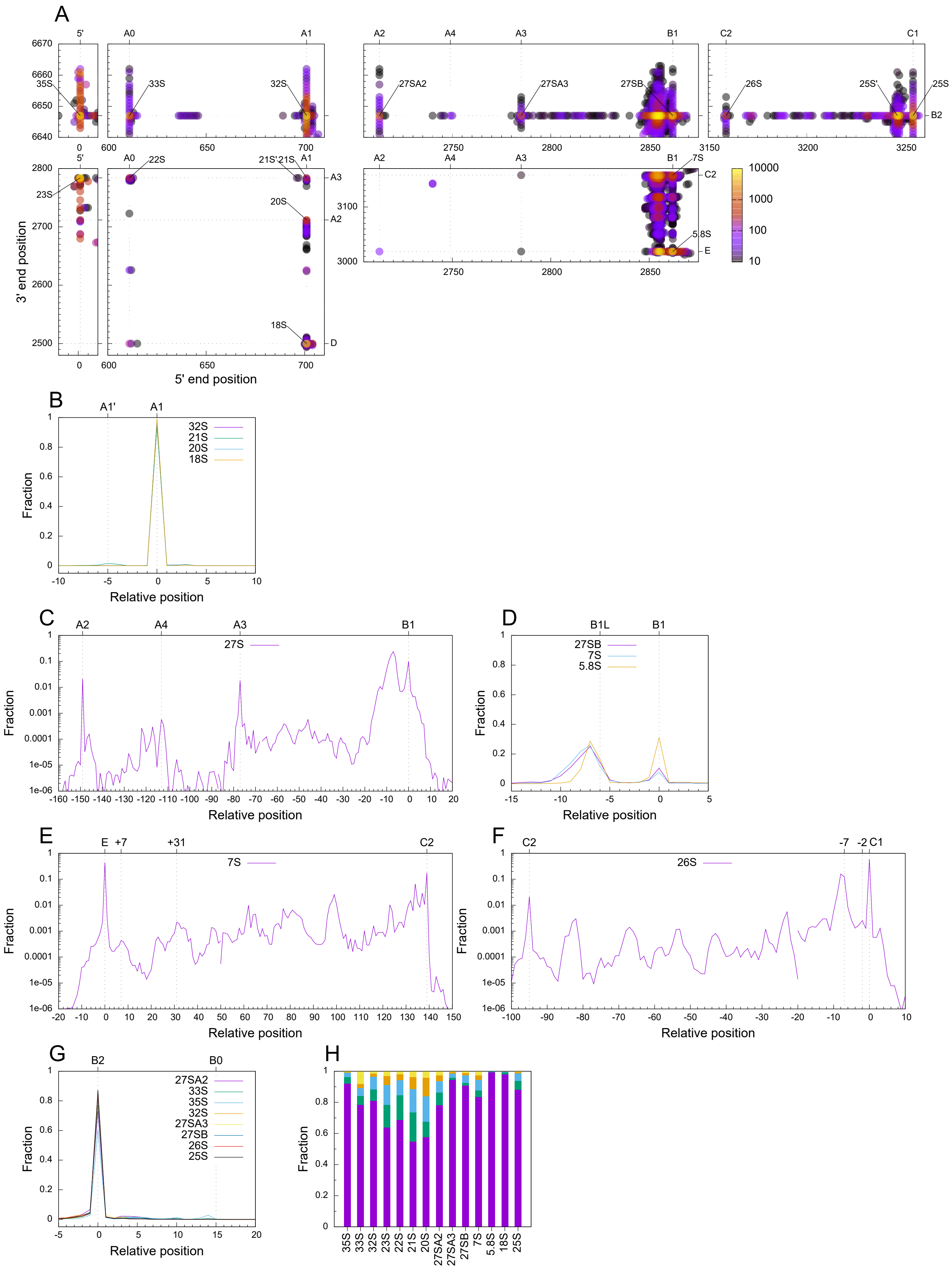

Fig S24. rat1 NOP7 rep1

I

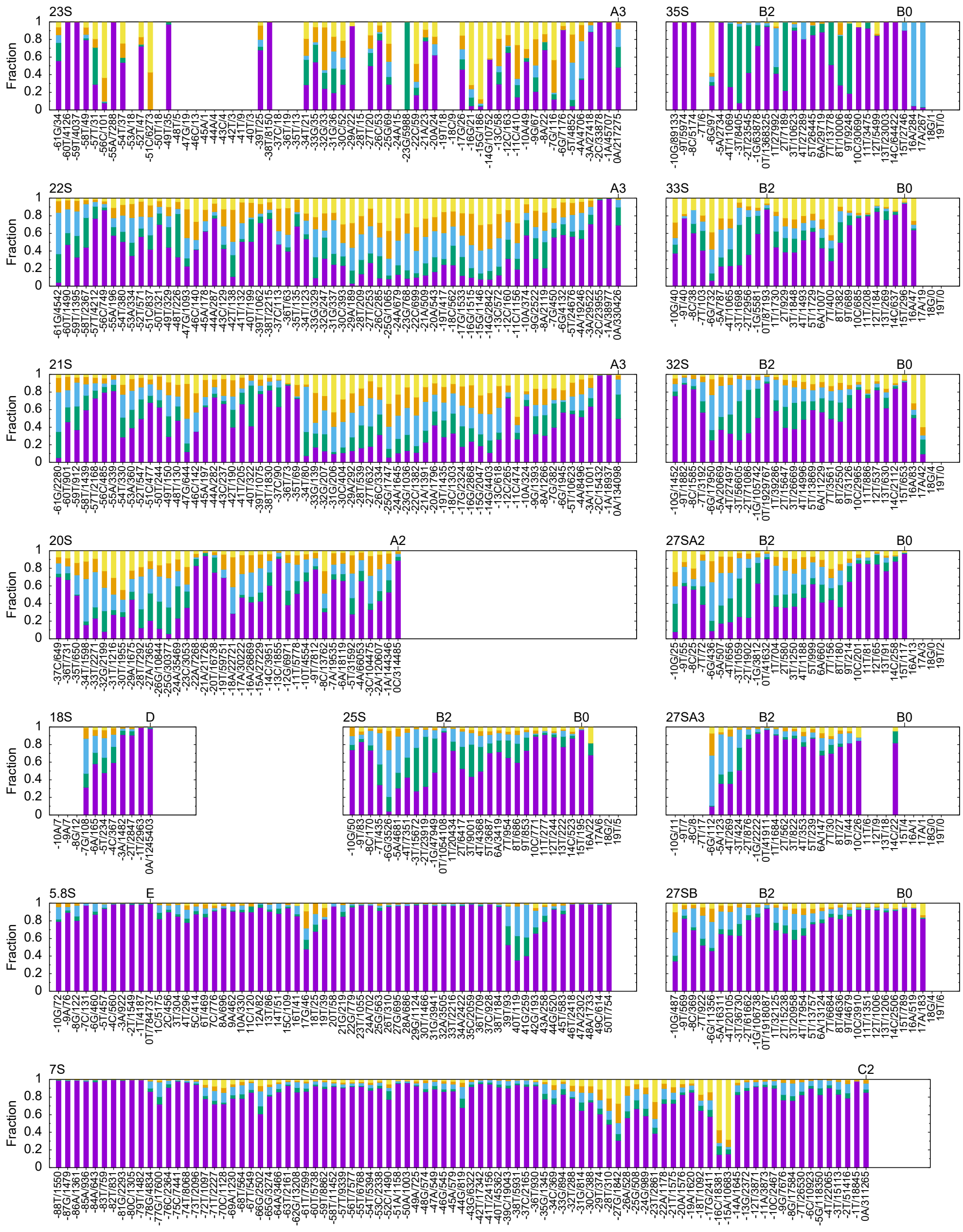

Fig S25. rat1 NOP7 rep2

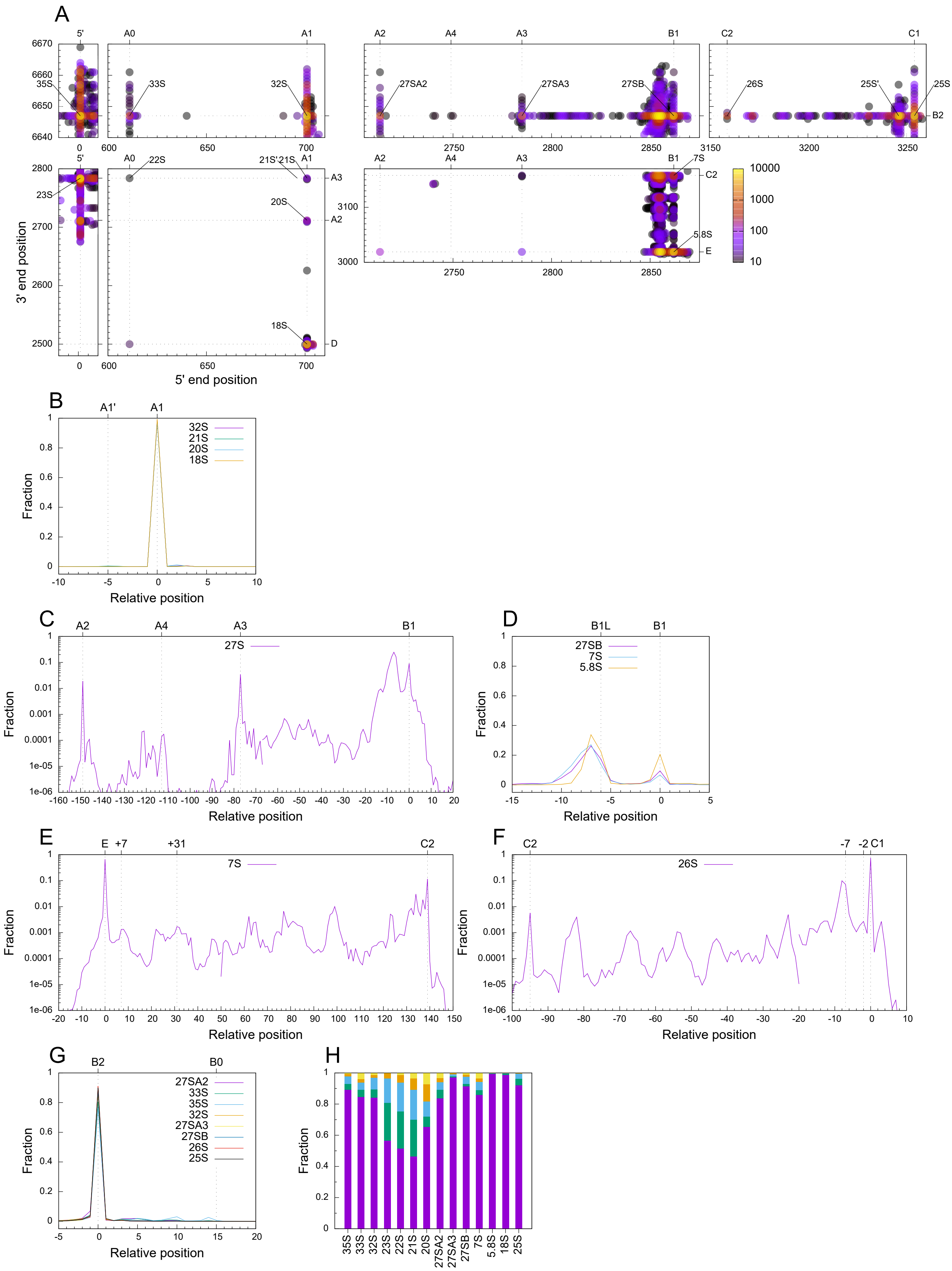

Fig S25. rat1 NOP7 rep2

I

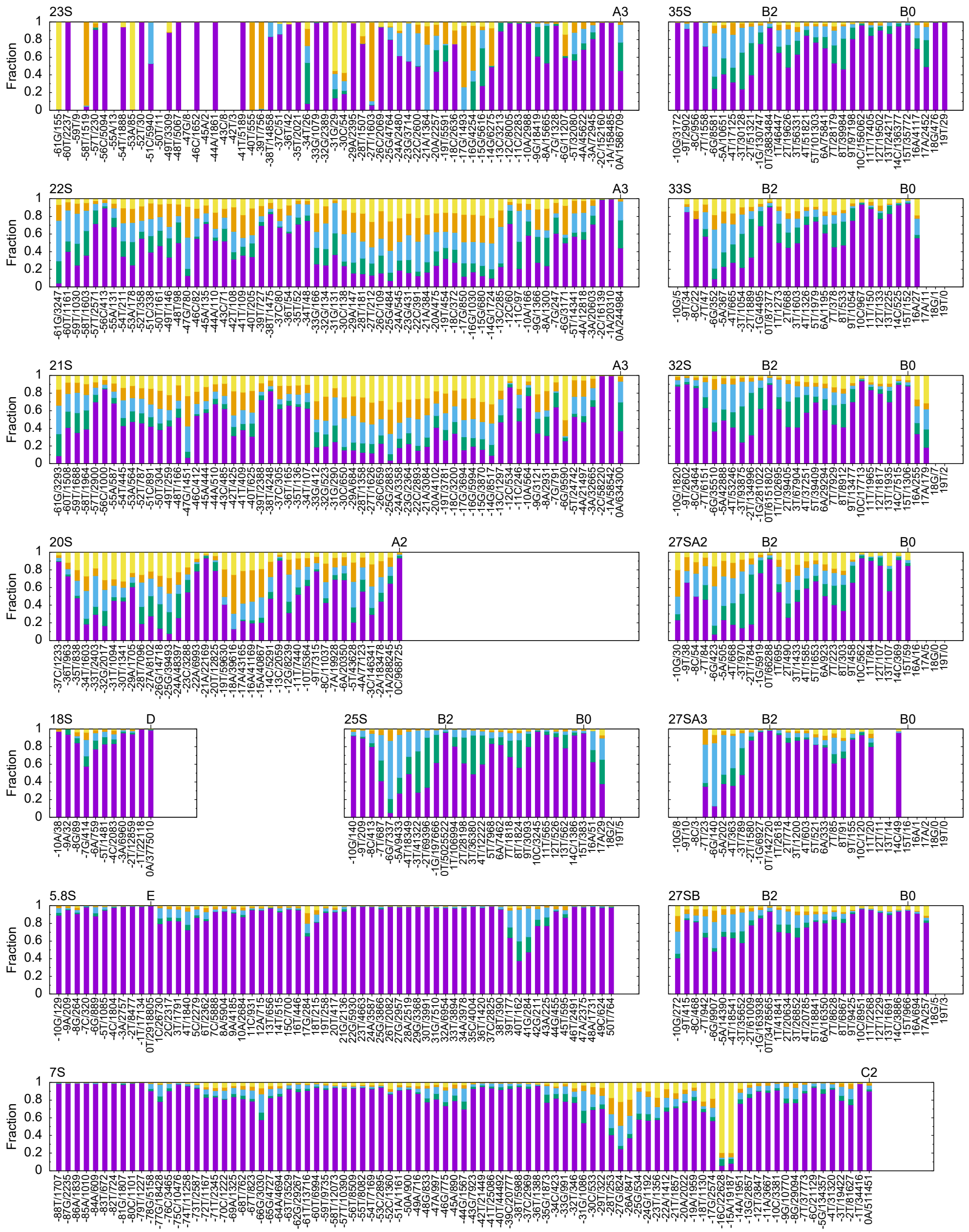

Fig S26. rrp17 NOP7 rep1

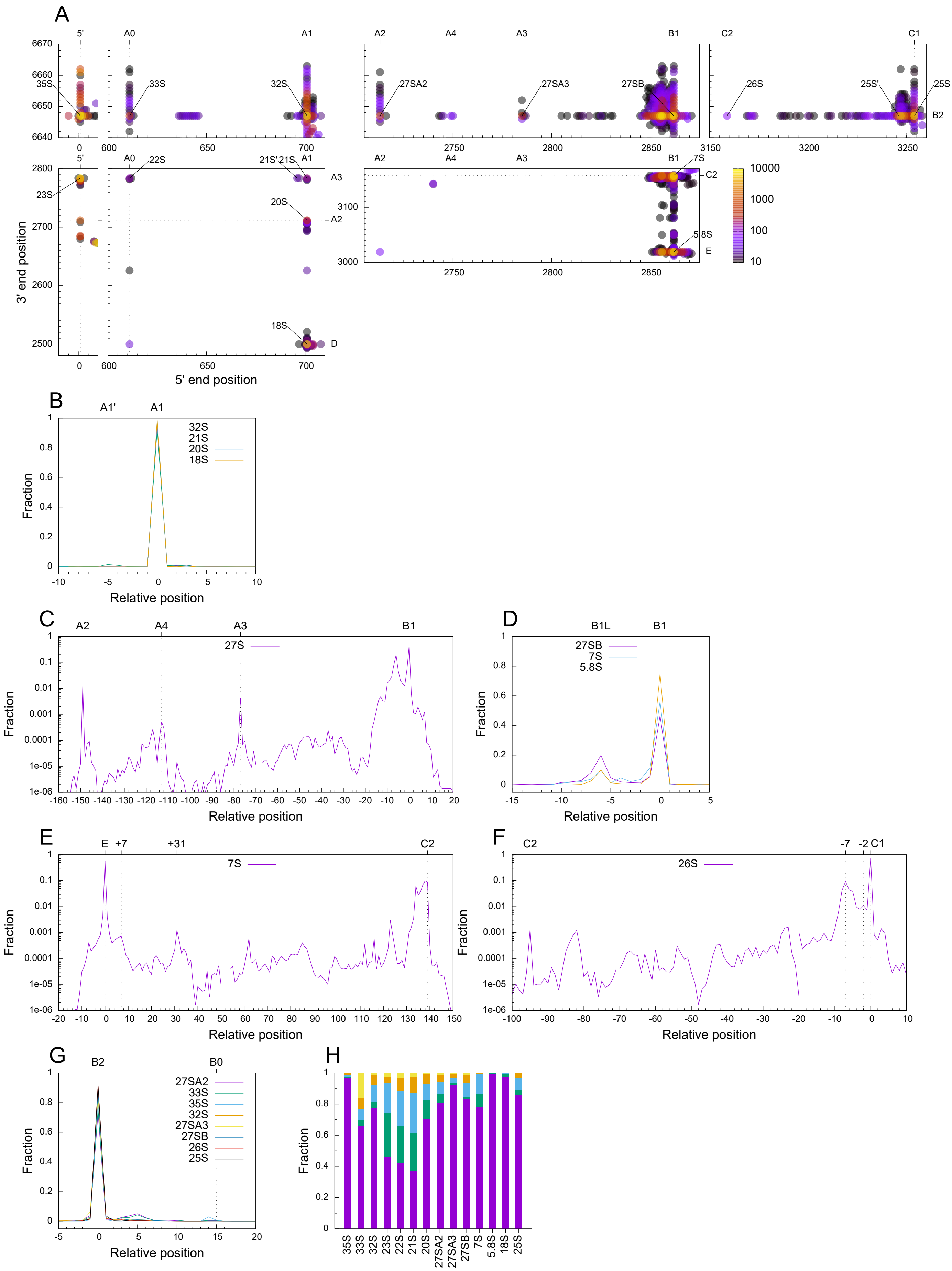

Fig S26. rrp17 NOP7 rep1

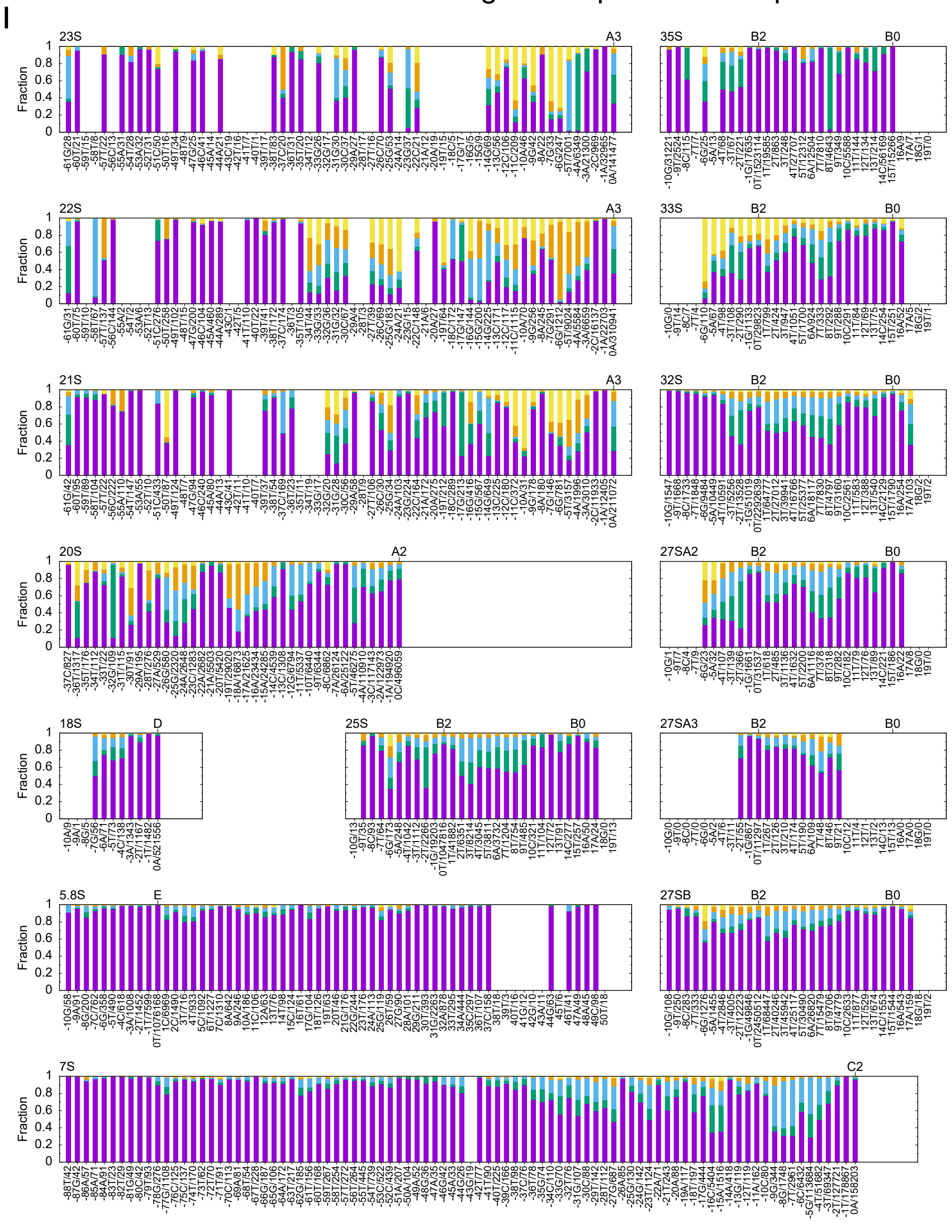

Fig S27. rrp17 NOP7 rep2

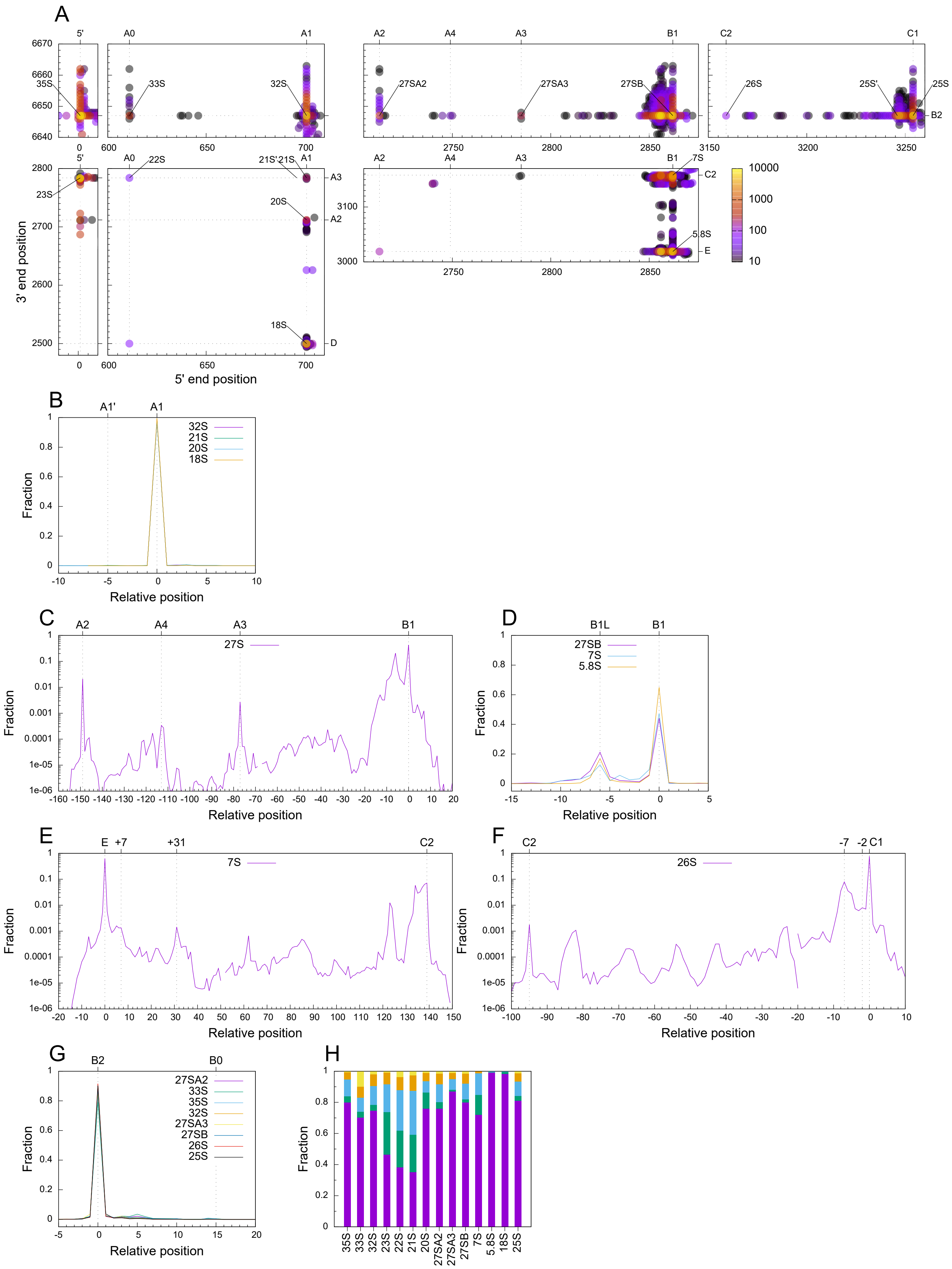

Fig S27. rrp17 NOP7 rep2

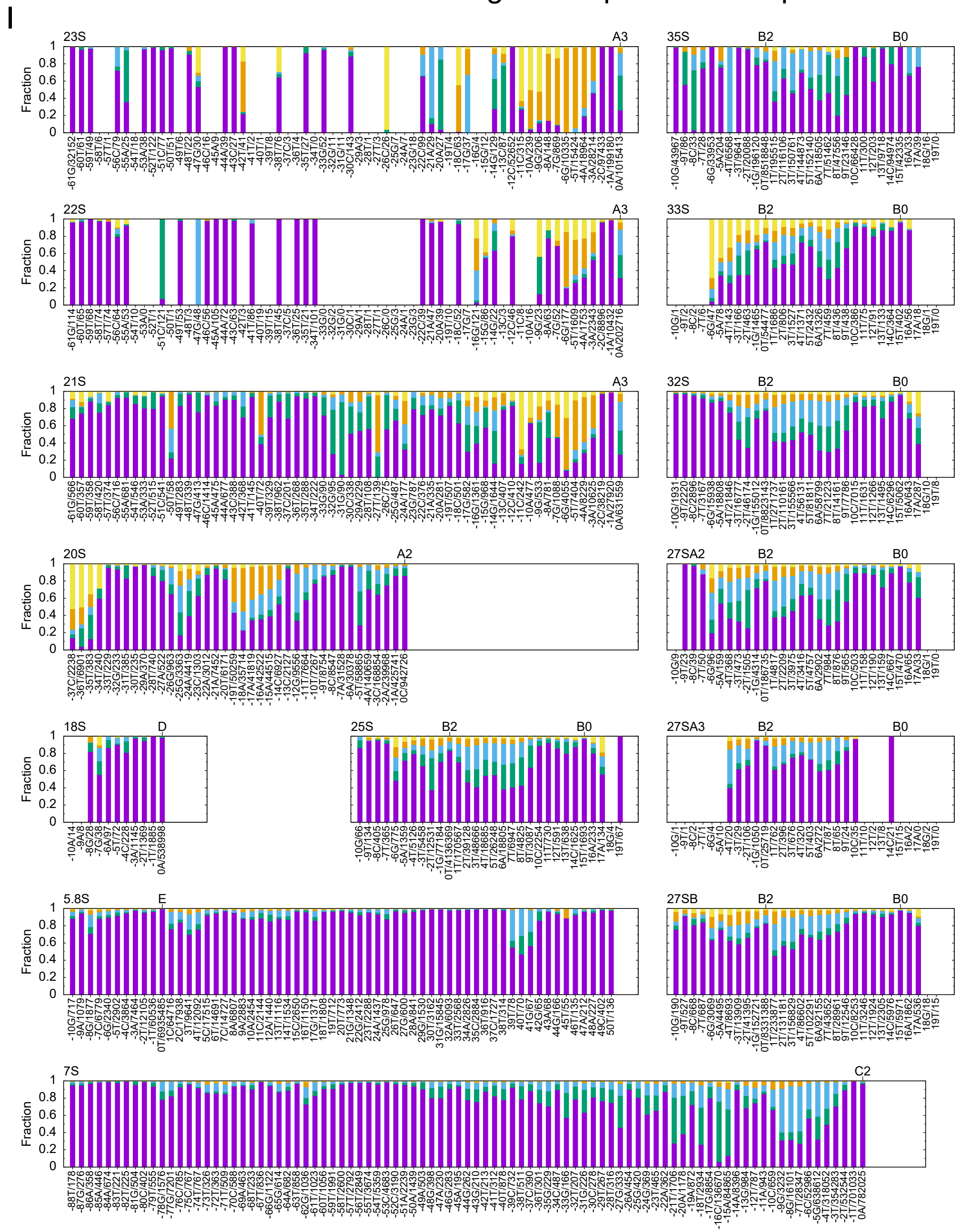

Fig S28. xrn1 NOP7

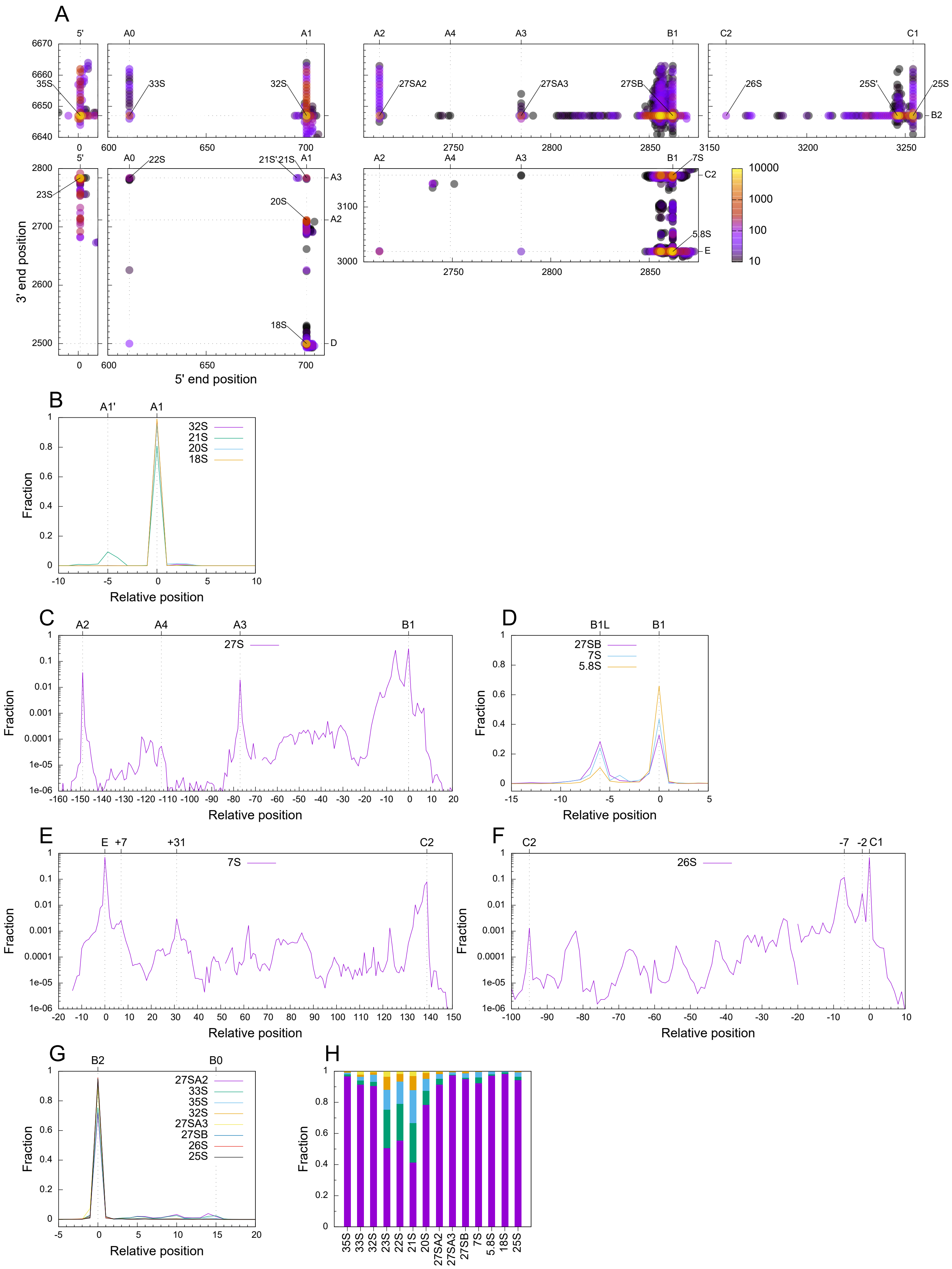

Fig S28. xrn1 NOP7

I

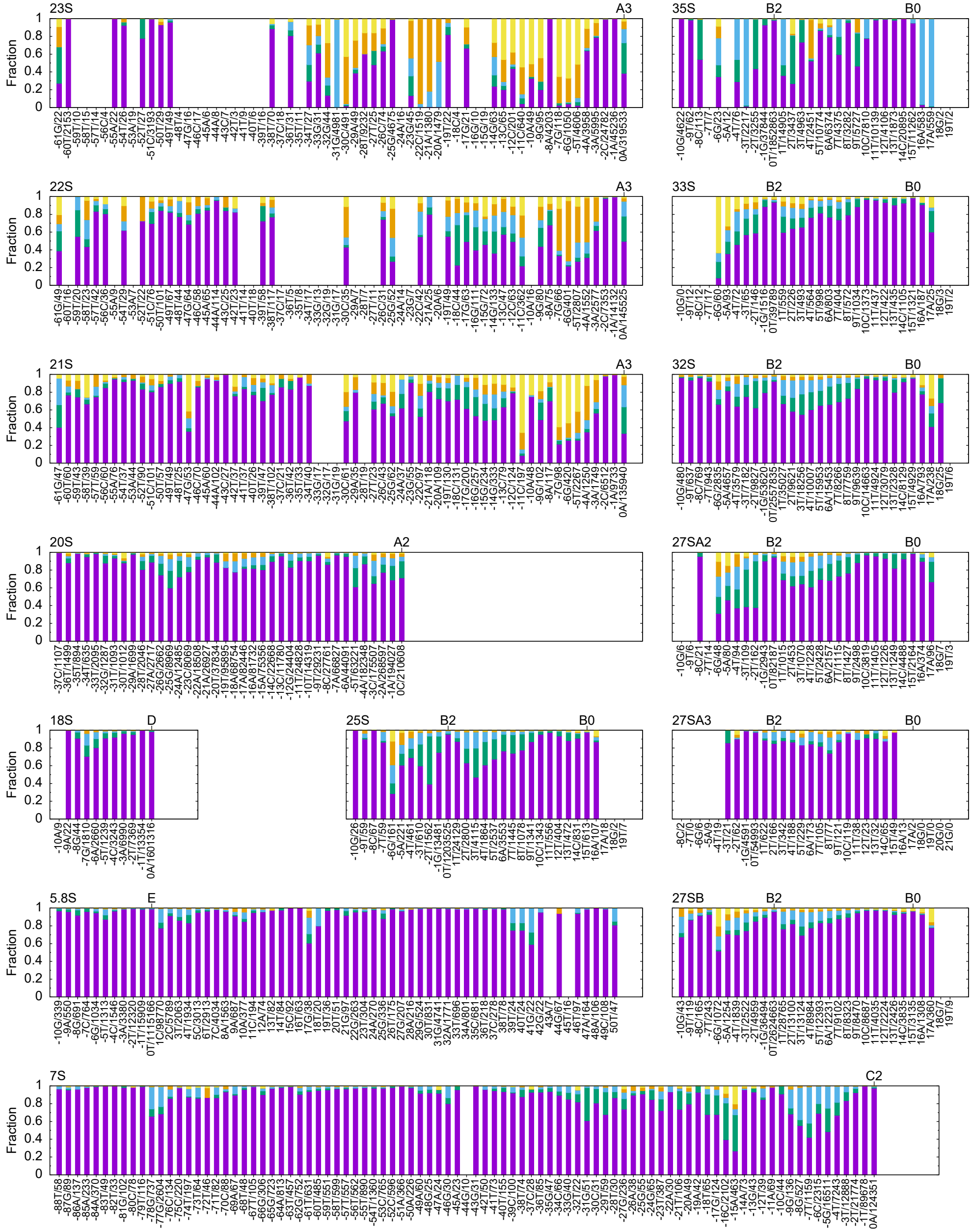

Fig S29. nme1 NOP7

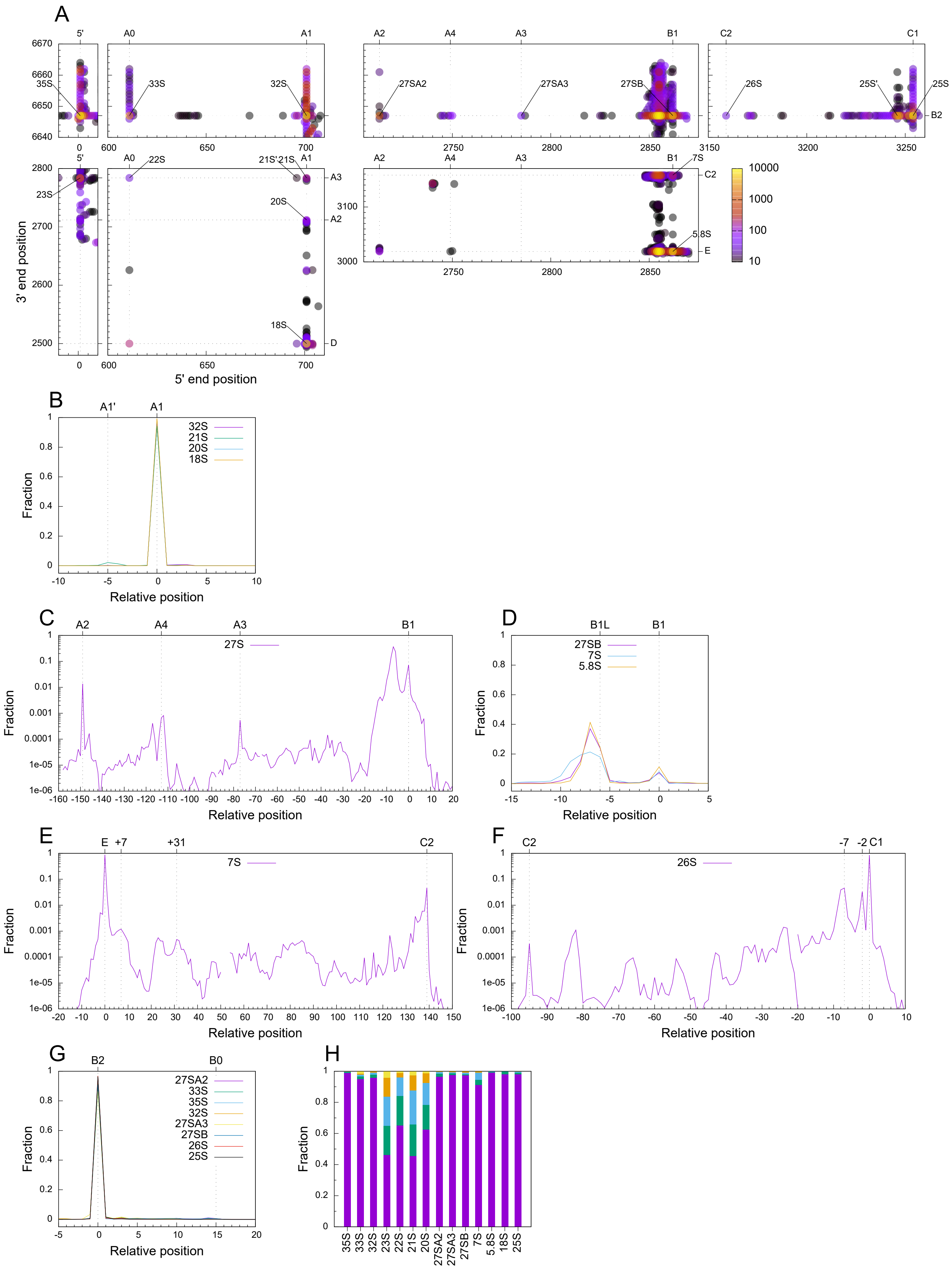

Fig S29. nme1 NOP7

I

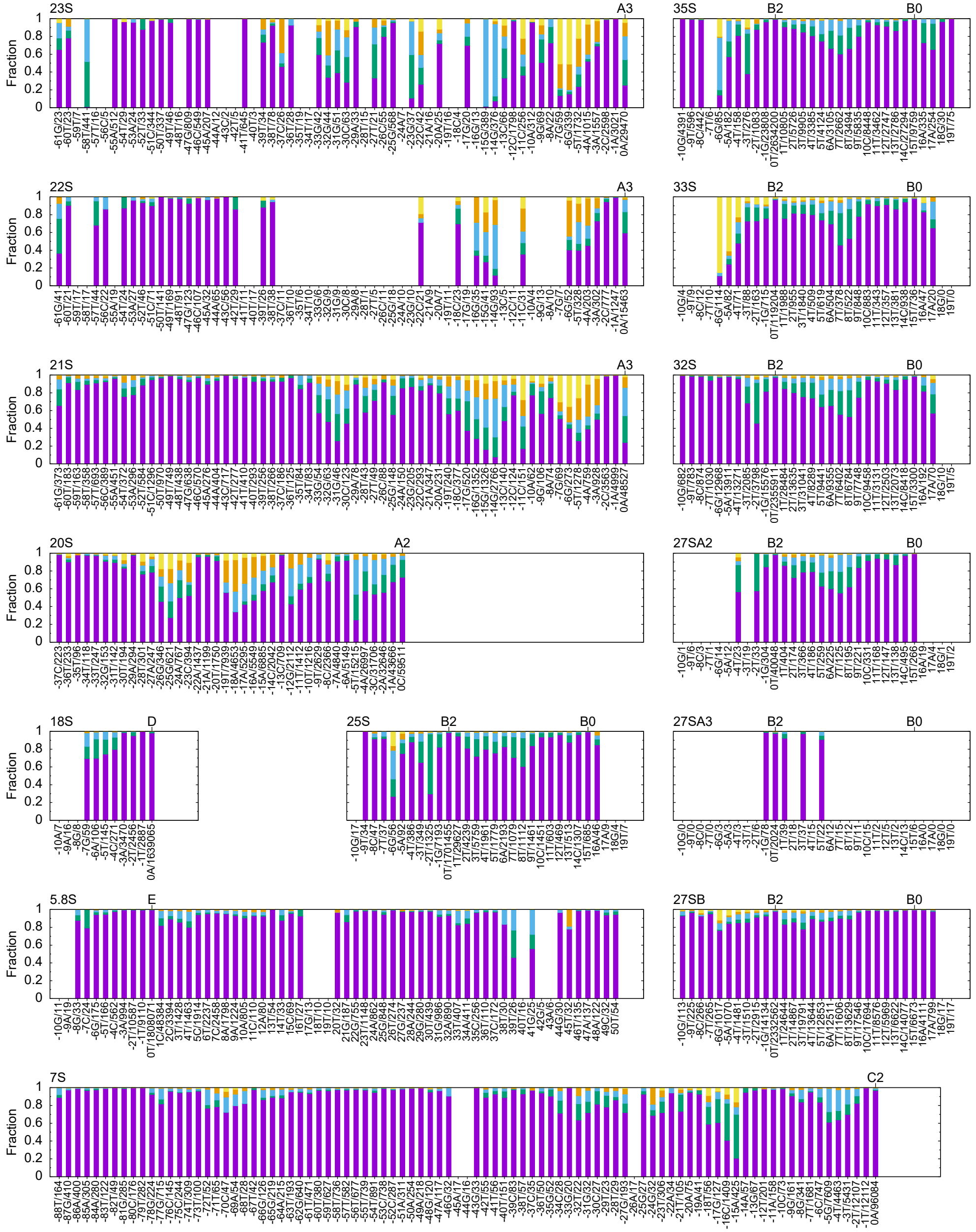

Fig S30. trf4 NOP7

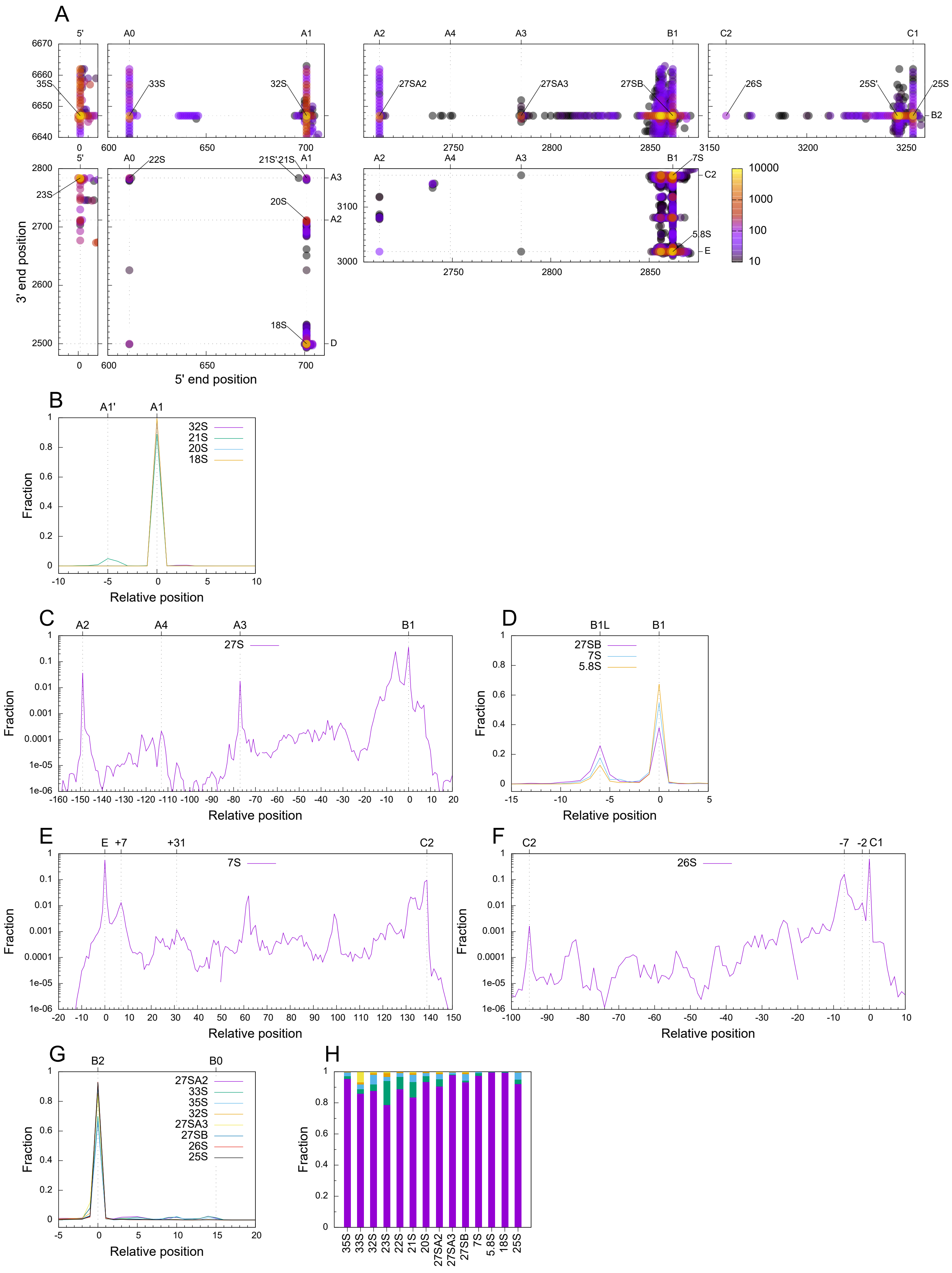

Fig S30. trf4 NOP7

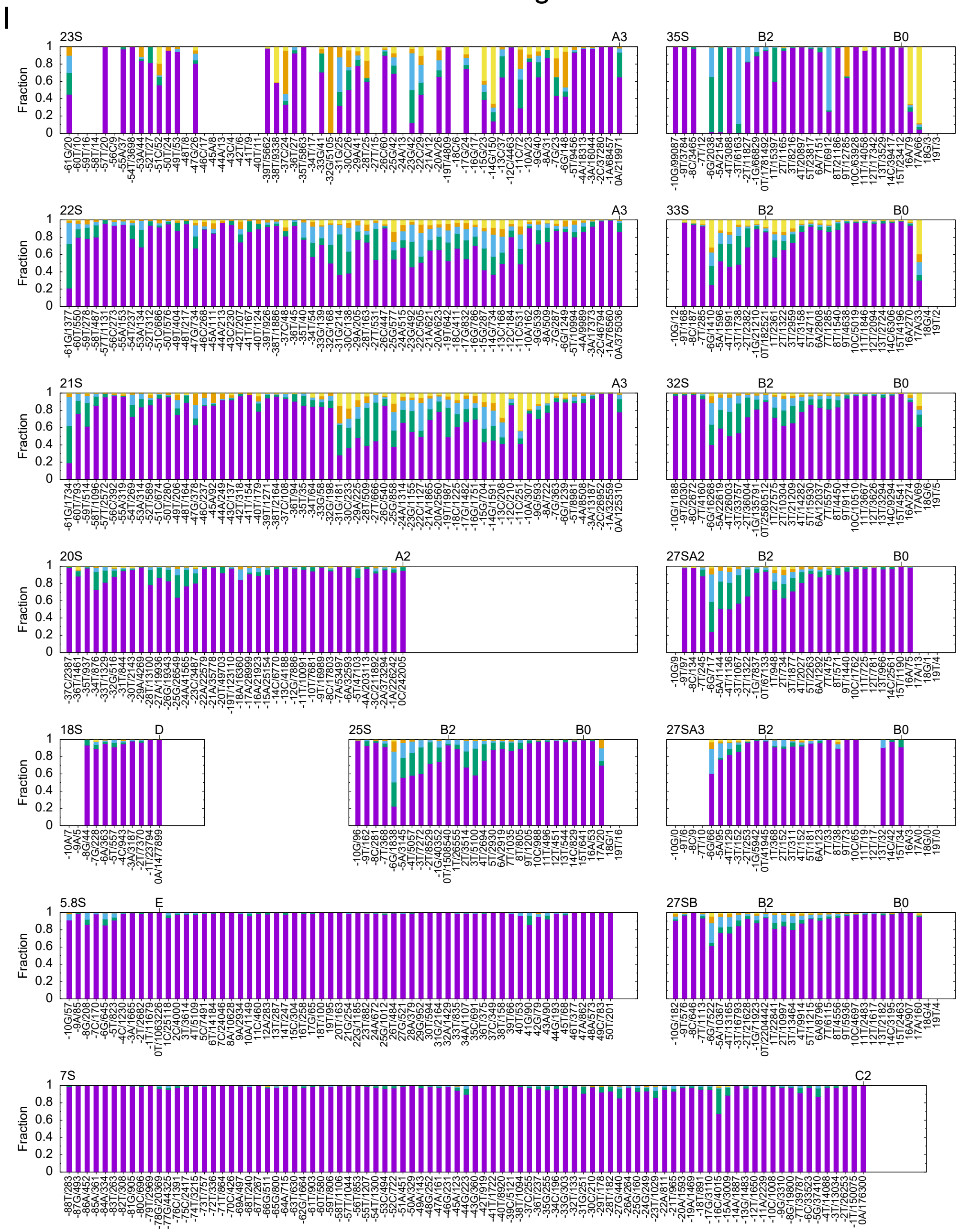

Fig S31. trf5 NOP7

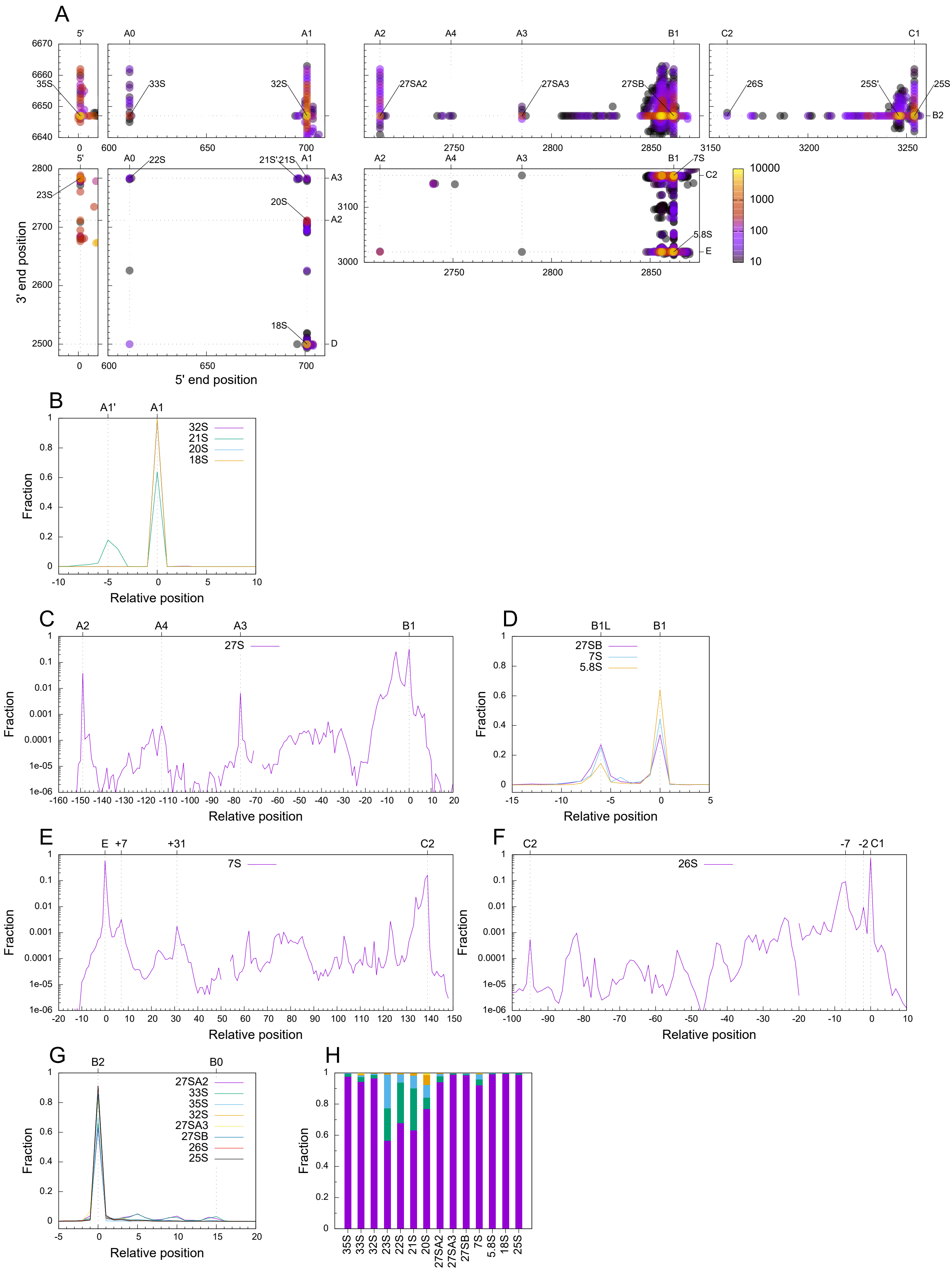

Fig S31. trf5 NOP7

I

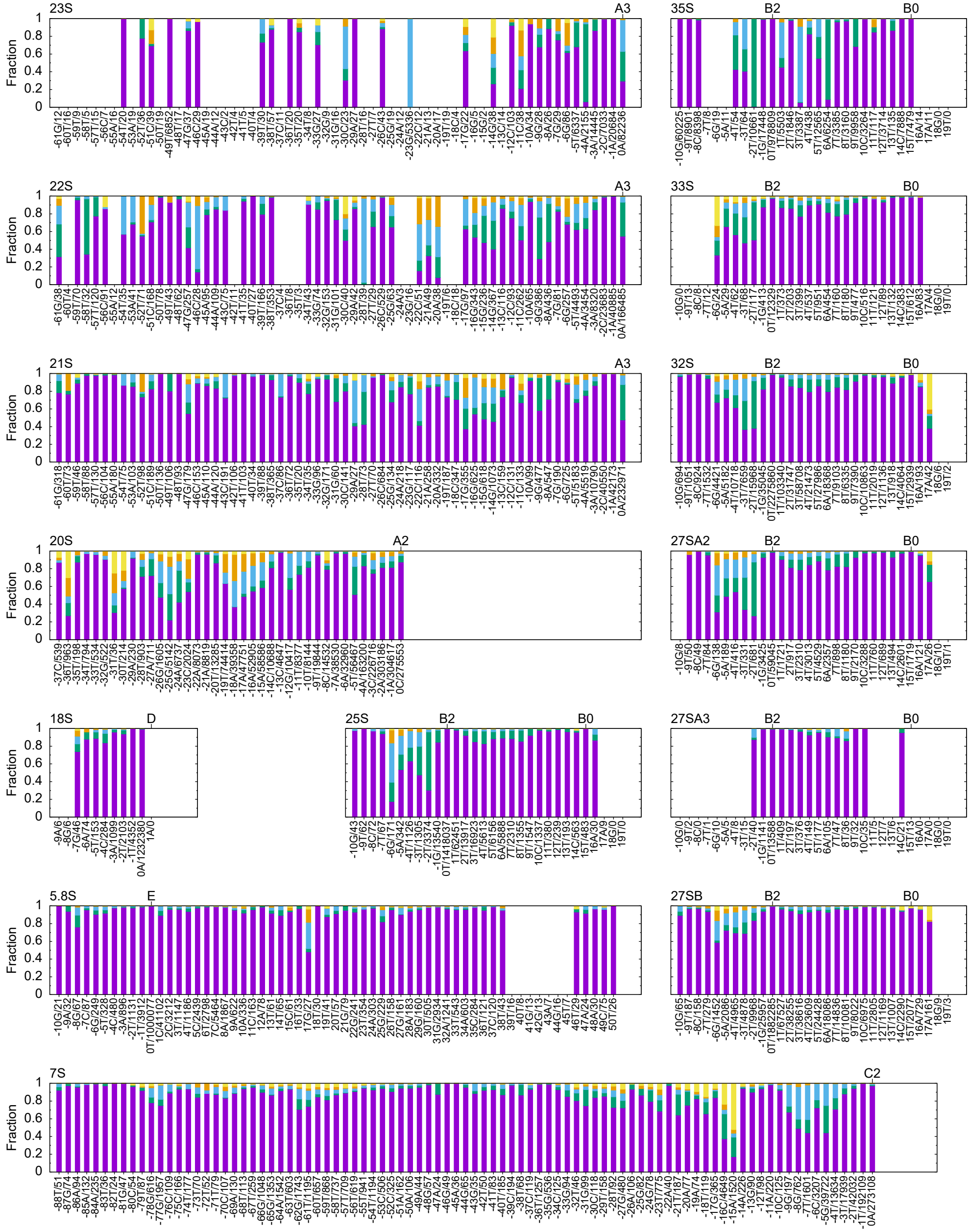

Supplement: gkae606_Supplemental_File [file gkae606_supplemental_file.pdf]
